# Regioselective Synthesis and Molecular Docking Studies of 1,5-Disubstituted 1,2,3-Triazole Derivatives of Pyrimidine Nucleobases

**DOI:** 10.3390/molecules27238467

**Published:** 2022-12-02

**Authors:** Vincenzo Algieri, Paola Costanzo, Matteo Antonio Tallarida, Fabrizio Olivito, Antonio Jiritano, Giulia Fiorani, Francesca Peccati, Gonzalo Jiménez-Osés, Loredana Maiuolo, Antonio De Nino

**Affiliations:** 1 Department of Chemistry and Chemical Technologies, University of Calabria, 87036 Rende, Italy; 2 Center for Cooperative Research in Biosciences (CIC bioGUNE), Basque Research and Technology Alliance (BRTA), Bizkaia Technology Park, 48160 Derio, Spain; 3 Department Molecular Sciences and Nanosystems, University Ca’ Foscari Venezia, 30172 Mestre (VE), Italy; 4 Ikerbasque, Basque Foundation for Science, 48013 Bilbao, Spain

**Keywords:** 1,2,3-triazoles, nucleobases, Lewis acid, click chemistry, molecular docking, ADME

## Abstract

1,2,3-triazoles are versatile building blocks with growing interest in medicinal chemistry. For this reason, organic chemistry focuses on the development of new synthetic pathways to obtain 1,2,3-triazole derivatives, especially with pyridine moieties. In this work, a novel series of 1,5-disubstituted-1,2,3-triazoles functionalized with pyrimidine nucleobases were prepared via 1,3-dipolar cycloaddition reaction in a regioselective manner for the first time. The *N*1-propargyl nucleobases, used as an alkyne intermediate, were obtained in high yields (87–92%) with a new two-step procedure that selectively led to the monoalkylated compounds. Then, FeCl_3_ was employed as an efficient Lewis acid catalyst for 1,3-dipolar cycloaddition between different aryl and benzyl azides and the *N*1-propargyl nucleobases previously synthesized. This new protocol allows the synthesis of a series of new 1,2,3-triazole derivatives with good to excellent yields (82–92%). The ADME (Absorption, Distribution, Metabolism, and Excretion) analysis showed good pharmacokinetic properties and no violations of Lipinsky’s rules, suggesting an appropriate drug likeness for these new compounds. Molecular docking simulations, conducted on different targets, revealed that two of these new hybrids could be potential ligands for viral and bacterial protein receptors such as human norovirus capsid protein, SARS-CoV-2 NSP13 helicase, and metallo-β-lactamase.

## 1. Introduction

The synthesis of triazole compounds, such as 1,2,3- and 1,2,4-triazoles, is of interest due to their versatility and usefulness in several fields including agriculture [1,2], material sciences [3,4], chemistry [5,6,7], as well as medicine [8,9,10]. In particular, the use of 1,2,3-triazoles as potential pharmacophores was intensively investigated. Thanks to their ability to form various non-covalent and dipole–dipole interactions, they were tested towards different biological targets [11]. From a synthetic point of view, 1,2,3-triazoles derivatives have attracted considerable attention after the independent discoveries of Meldal and Sharpless in the early 2000s, which allowed the regiospecific synthesis of 1,4-disubstituted 1,2,3-triazoles via click chemistry [12,13]. Furthermore, in 2005, Zhang and co-workers showed that it was also possible to attain the regioselective synthesis of 1,5-disubstituted 1,2,3-triazoles by changing copper to ruthenium [14].

**Figure 1:**
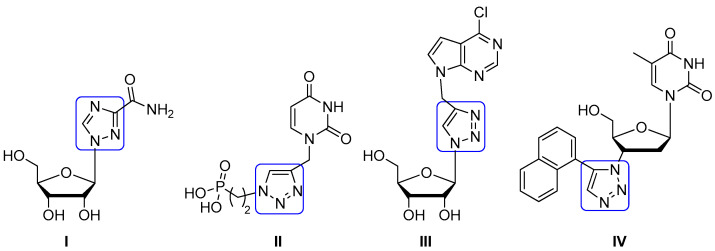
Some reported anticancer agents bearing triazole, nucleobase, and/or glycoside moieties.

Other transition metal catalysts (Au, Ir, Ni, Ag) were tested [15], but despite the huge efforts, the employment of heavy metals in the synthesis restricts their application due to their hazardous nature, toxicity, and high cost. In recent years, great attention was paid to the development of copper- and ruthenium-based alternative routes, and different protocols were developed [16]. Besides 1,2,3-triazoles, natural and modified nucleosides are another class of heterocyclic compounds with widespread applications as pharmaceutically active compounds [17,18] and diagnostic probes [19]. Considering the numerous advantages of using both triazoles and nucleosides as building blocks in drug discovery, many researchers synthesized nucleosides containing the triazole moiety [20,21]. Starting from the discovery of the broad-spectrum antiviral compound Ribavirin **I** (Figure 1), in which a 1,2,4-triazole was used as a nucleobase, different new hybrids were designed and tested, showing antiviral and/or antitumor activity. These nucleoside analogues were characterized by the introduction of a triazole group in place of the nucleobase **I** [22], or the sugar moiety **II** [23], as a linkage between them **III** [24], and as a modification group of the sugar **IV** [25] (Figure 1).

To date, only a few examples of 1,4-disubstituted 1,2,3-triazole nucleoside derivatives were synthesized. These compounds were tested as corrosion inhibitors for steel [26], against HCV (Hepatitis C virus) [23], and against influenza virus A (H3N2) [27]. However, to the best of our knowledge, there have been no reported synthetic efforts toward 1,5-disubstituted analogues.

In this work, considering our experience in the development of alternative synthetic routes catalyzed by Lewis acids [28,29] and cycloaddition reactions [30,31], we report the synthesis of new 1,5-disubstituted 1,2,3-triazole derivatives containing pyrimidine nucleobases in high yields and a regioselective way. All of the triazole derivatives were synthesized starting from different aryl azides and non-commercial *N*1-propargyl pyrimidine nucleobases using a common Lewis acid catalyst. In detail, iron(III) chloride was selected as a suitable catalyst because of its low price, easy availability, sustainability, nontoxicity, and environmentally friendly characteristic [32,33]. Moreover, the use of *N*1-propargyl pyrimidine nucleobases allows the introduction of a methylene bridge at C-5 of the triazole, which could mimic the behavior of “fleximers”, a kind of nucleoside analogue characterized by enhanced conformational freedom [34]. The pharmacokinetic properties of the synthesized derivatives were predicted through ADME (Absorption, Distribution, Metabolism, and Excretion) analysis to evaluate the medicinal chemistry friendliness. Furthermore, an in silico screening was performed towards selected receptors from the Protein Data Bank, suggesting biological potential for our products.

## 2. Results and Discussion

### 2.1. Chemistry

**Scheme 1:**
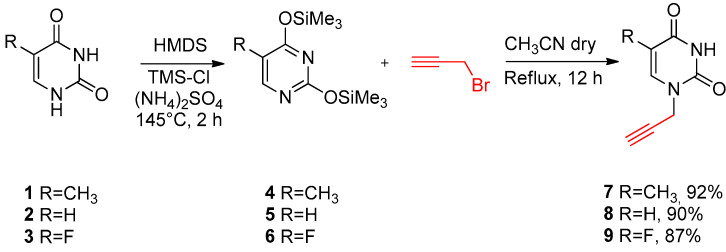
Synthesis of propargyl nucleobases with two reaction steps.

*N*1-propargyl nucleobases are important starting materials for the synthesis of nucleoside analogues with biological activity [35]. Usually, propargyl nucleobases are synthesized in a one-step reaction between the appropriate nucleobase and propargyl bromide under basic conditions [36] or employing an intermediate bis(trimethylsilyl)pyrimidine nucleobase using *N,O*-bis(trimethylsilyl)-acetamide (BSA) [37]. Unfortunately, the first methodology yields the products with low selectivity [38] because a mixture composed of *N*1-monoalkylated and *N*1,*N*3-dialkylated pyrimidines is obtained, while the second approach results in long reaction times and low yield [26]. With this in mind, selective nucleobase propargylation is necessary, and, due to the feasibility of performing selective alkylation at the *N*-1 position of pyrimidine nucleobases, the propargylation is performed in two-reaction steps via *O*-protection by a transient group, as shown in Scheme 1.

Here, we developed a methodology that involves the *N*1-selective propargylation of bis(trimethylsilyl)pyrimidine intermediates (**4**–**6**) to prepare compounds **7**–**9**. Thus, pyrimidine nucleobases **1**–**3** were treated under an inert atmosphere with hexamethyldisilazane (HMDS), trimethylsilyl chloride (TMS-Cl), and (NH_4_)_2_SO_4_ to give the silylated nucleobases **4**–**6**, which were used without further purification and isolated by under vacuum evaporation of HMDS. In situ propargylation of **4**–**6**, conducted in dry acetonitrile at reflux under an inert atmosphere, furnished the desired products **7**–**9** with excellent reaction yields and in a regioselective way because only *N*-1 monosubstituted nucleobases were observed in all cases.

With compounds **7**–**9** in hand, we then performed a Lewis acid-catalyzed azide-alkyne 1,3-dipolar cycloaddition reaction to generate a series of 1,5-disubstituted 1,2,3-triazole derivatives of nucleobases in a regioselective way.

**Scheme 2:**
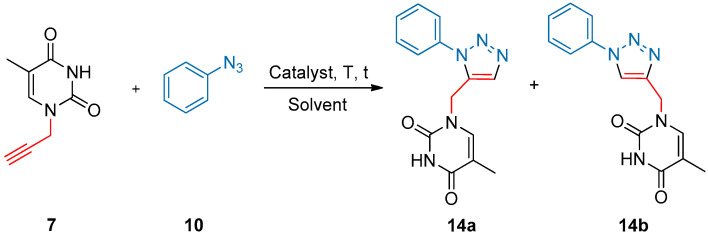
Azide-alkyne 1,3-dipolar cycloaddition to synthesize 1,5-disubstituted-1,2,3-triazoles functionalized with pyrimidine nucleobases.

To begin, we chose the cycloaddition reaction between 1-propargylthymine **7** and phenyl azide **10** in the presence of Lewis acid catalysts as the model system to optimize the reaction conditions (Scheme 2). The results are reported in Table 1.

Firstly, the reaction between 1-propargylthymine **7** and phenyl azide **10**, in a 1:2 ratio, was performed with 20 mol% Er(OTf)_3_ as the catalyst and heated at 60 °C both in CH_2_Cl_2_ (Table 1, entry 1) and THF (Table 1, entry 2). In both cases, no product formation was observed after 24 h of time reaction. An increase in temperature to 100 °C favored a slight formation of the product with a yield of 15% (Table 1, entry 3). Changing THF to CH_3_CN allowed a small yield increase in 24 h (Table 1, entry 4). Better results were obtained by raising the temperature to 120 °C (Table 1, entry 5), and also using nitromethane as a solvent (Table 1, entry 6). However, the use of DMF as a solvent and Er(OTf)_3_ as the catalyst at 120 °C provided good yields in only 8 h (Table 1, entry 7). At this point, a screening of different Lewis acids was performed (Table 1, entries 8–11) and the best reaction conditions were found when FeCl_3_ was used as the catalyst (Table 1, entry 11), leading to 88% yield in 8 h. The role of the catalyst was remarkable since it selectively led to one regioisomer, corresponding to the 1,5-disubstituted 1,2,3-triazole **14a**. However, the regioisomer **14b** was also isolated and characterized by ^1^H NMR and ^13^C NMR, and an 88:12 ratio was calculated by ^1^H NMR analysis of the reaction crude (see Supplementary Materials). In fact, without any catalyst, reagents **7** and **10** under the same reaction conditions gave the 1,5-disubstituted triazoles **14** in low yield after a long reaction time (Table 1, entry 12) and a mixture of the 1,5-disubstituted/1,4-disubstituted products was observed in a 63:37 ratio. This last result suggests that the Lewis acid catalyst accelerates the reaction by increasing the electrophilicity of the alkyne group through coordination. Although the iron(III) chloride efficacy as a catalyst in an eliminative azide–olefin cycloaddition was already demonstrated for the synthesis of 1,5-disubstituted triazoles [39], we were delighted to observe such regioselectivity. Moreover, a comparison with classical cycloaddition reaction conditions was made in toluene without any catalyst. A regioisomer mixture in a 60:40 ratio was obtained in low yield after a long reaction time (Table 1, entry 13). The addition of a catalyst slightly increased the yield without significantly improving regioselectivity (Table 1, entry 14). Finally, the 1,3-dipolar cycloaddition reaction was carried out in [mPy](OTf) ionic liquid (Table 1, entry 15), prepared as reported in the literature [40], with the purpose of hypothetically recycling the solvent. Unfortunately, due to the high solubility of the pyrimidine in the ionic liquid, it was not possible to use the latter as a solvent because it was impossible to purify the product by simple liquid–liquid extraction.

**Scheme 3:**
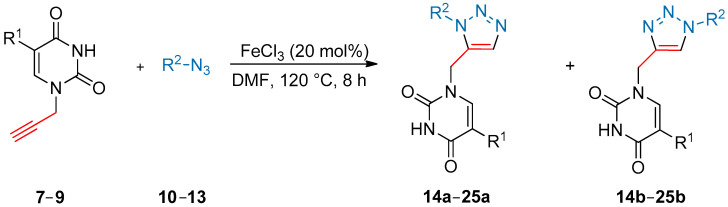
1,5-disubstituted-1,2,3-triazoles functionalized with pyrimidine nucleobases generated from various azides and propargyl nucleobases.

Once the reaction conditions were optimized, we extended the protocol to various *N*1-propargyl nucleobases **7**–**9** and different azides **10**–**13** (Scheme 3, Table 2).

As shown in Table 2, the *N*1-propargyl nucleobases **7**–**9** showed a high reactivity towards the iron(III)-catalyzed 1,3-dipolar cycloaddition, and high reaction yields were obtained. Conversely, the reactivity of the azides depended on the nature of the aryl or alkyl group. In fact, benzyl azide **11** provided products **17a**–**19a** (Table 2, entries 4–6) with slightly lower yields than phenyl azide **10** (Table 2, entries 1–3).

Aromatic azides **10**, **12**, and **13** exhibited different reactivities depending on the substitution of the aromatic ring. In fact, the presence of a strong electron-withdrawing group (-NO_2_) at the *para* position reduced the nucleophilicity of the azide **12** (Table 2, entries 7–9), while the presence of the electron-donor group (CH_3_O^−^) at the same position increased its nucleophilicity (azide **13**, Table 2, entries 10–12). Hence, the reaction yield for the regioisomer **a**, as an isolated compound, was excellent in all cases. In addition, only a single regioisomer was obtained for all synthesized compounds, proving the high regioselectivity of the iron(III)-catalyzed reaction.

**Scheme 4:**
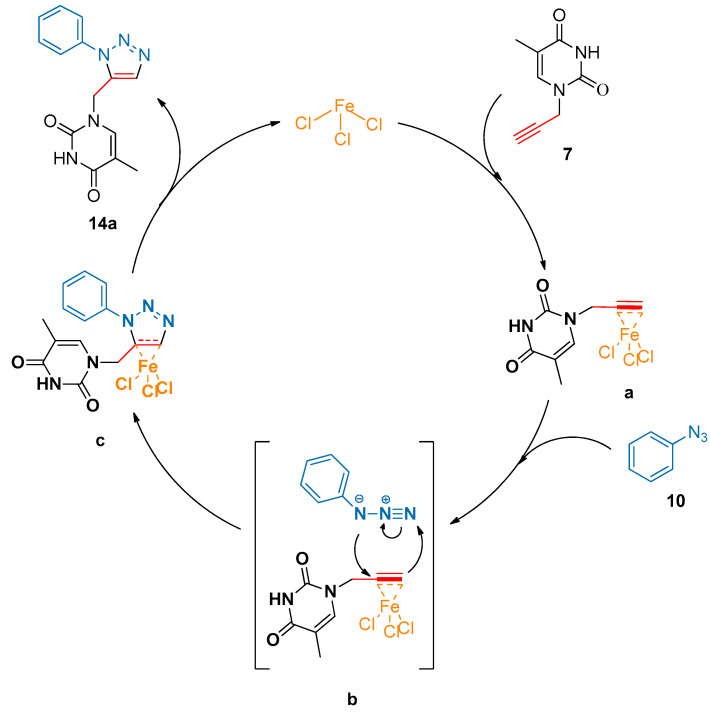
Proposed catalytic cycle for the regioselective iron(III)-catalyzed azide-alkyne 1,3-dipolar cycloaddition reaction.

Finally, we proposed a possible catalytic reaction mechanism for the reaction between **7** and **10** as illustrated in Scheme 4. The first reaction step is the coordination of iron(III) chloride to the alkyne group of *N*-propargylthymine **7**, generating intermediate **a** with increased electrophilicity. Activated dipolarophile **a** reacts with phenyl azide **10** through a concerted 1,3-dipolar cycloaddition reaction to give intermediate **c**. Subsequent heterocycle aromatization of **c** gives product **14** and allows catalyst turnover.

### 2.2. Molecular Docking

**Figure 2:**
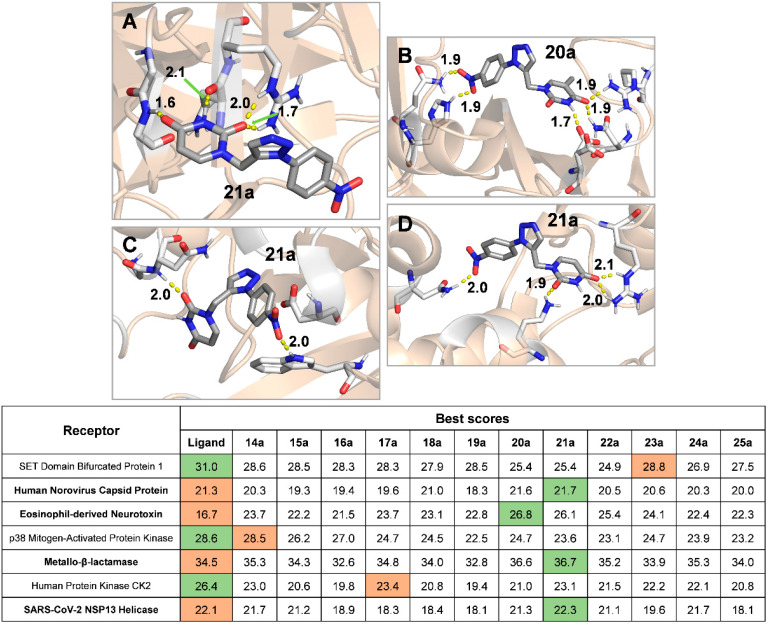
Best docking poses of compounds **14a**–**25a** vs. co-crystalized ligands on selected protein receptors: (**A**) Compound **21a** bound to human norovirus capsid protein (PDB 6GY9). (**B**) Compound **20a** bound to eosinophil-derived neurotoxin (PDB 5E13). (**C**) Compound **21a** bound to metallo-β-lactamase (PDB 7OVE). (**D**) Compound **21a** bound to SARS-CoV-2 NSP13 helicase (PDB 7NNG). Ligands and binding residues are shown as sticks. Non-polar hydrogens have been omitted for clarity. In the table, the docking scores of compounds **14a**–**25a** and literature ligands were compared. The green color refers to scores higher than the literature ligands. The orange color refers to scores lower than the literature ligands. For those cases in which the docking score was higher than literature ligands the receptors are reported in bold.

Finally, to evaluate the potential biological activity of our products, molecular docking simulations were carried out using GOLD (CCDC Discovery) [41] and the ChemScore scoring function [42]. An in silico study was performed over 16 targets for the full set of the compounds synthesized in this work (see Supplementary Materials for further details). Compounds **20a** and **21a** were the best ranked ones (Figure 2), matching or exceeding in some cases the score of the co-crystallized ligand in the original structure, suggesting that these compounds might be able to bind the selected targets. Such targets have been at the center of great attention in the last years due to their possible implication in different pathologies. Human norovirus is one of the major causes of nonbacterial gastroenteritis in humans, and targeting the protruding P domain dimer (P-dimer) of a GII.10 Norovirus strain (Figure 2A) could be a successful strategy in drug discovery [43]. Eosinophil-derived neurotoxin (EDN) (Figure 2B) is a member of the Ribonuclease A (RNase A) superfamily involved in inflammatory disorders and the immune response system [44]. Metallo β-lactamases (Figure 2C) are a family of enzymes employed by bacteria to hydrolyze β-lactam drugs as carbapenems, determining the resistance to antibiotics [45]. Hence, the discovery of new inhibitors capable of blocking such receptors could be of interest in combating bacterial infective diseases. As for the target reported in Figure 2D, SARS-CoV-2 NSP13 helicase was described by Newman et al. [46] in 2021 as a potential target for new antivirals due to its essential role in viral replication and its high sequence conservation.

Binding interactions mostly involve hydrogen bonds with the protein backbone or with charged residues, which are in general very much favored from the energetic point of view and capable of dictating the local structure [47].

### 2.3. Pharmacokinetics and ADMET Study

**Figure 3:**
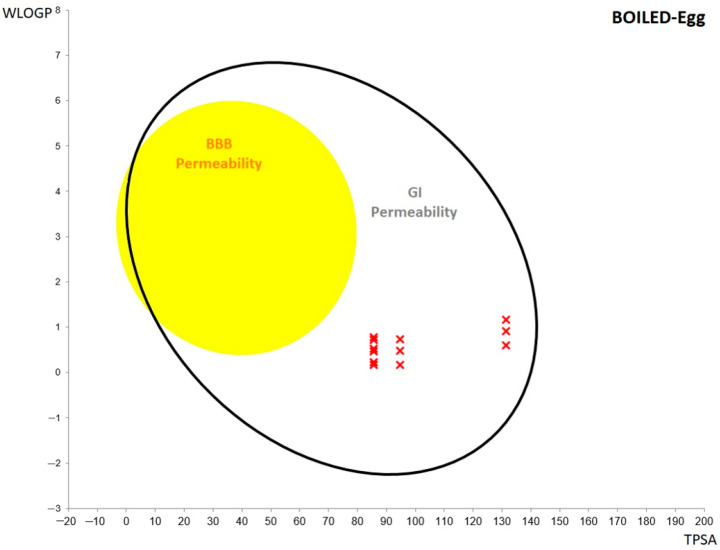
The BOILED-egg model of 1,5-disubstituted 1,2,3-triazole derivatives of pyrimidine nucleobases (**14a**–**25a**). BBB and GI permeability are indicated in yellow and colorless regions, respectively.

The drug-likeness of the newly synthesized hybrids was further evaluated. The analysis of absorption, distribution, metabolism, and excretion (ADME) was determined in silico by using the online database ADMETlab 2.0 [48]. As reported in Table 3, all hybrids expressed good ADME properties. They showed good water solubility and no violation of the Lipinsky rules of 5 [49]. Although the Caco-2 permeability was not excellent in some cases, effective intestinal absorption was found for all compounds, not only with the ADMETlab 2.0 software but also using the Brain Or IntestinaL EstimateD permeation method (BOILED-Egg) that evaluates the accessibility of compounds to the gastrointestinal (GI) tract and blood–brain barrier [50] (Figure 3). The boiled egg model revealed that all molecules had satisfactory GI absorption and no sufficient permeability across the blood–brain barrier (BBB), thus indicating good safety for the Central Nervous System (CNS). Moreover, all compounds had a good clearance rate (excretion rate) from the body. The encouraging results reported in Table 3 suggest that these compounds could be eligible as drug candidates, confirming the use of **20a** and **21a** as potential lead compounds for a new class of active pharmaceutical ingredients.

## 3. Materials and Methods

### 3.1. General Procedure for Nucleobase Propargylation ***7***–***9***

In a three-necked round-bottomed flask, equipped with a bubble condenser and magnetic stir bar, the opportune nucleobase **1**–**3** (39.6 mmol, 1 eq) in dry hexamethyldisilazane (HMDS, 139 mmol, 3.5 eq) was suspended under nitrogen atmosphere. Subsequently, trimethylsilyl chloride (8.71 mmol, 0.22 eq) and (NH_4_)_2_SO_4_ (1.98 mmol, 0.05 eq) were added, and the mixture was stirred at 145 °C for 2 h. After completion, the solution was cooled to room temperature, and the HMDS was evaporated under vacuum. Then, the obtained silylated nucleobase **4**–**6** was dissolved in dry acetonitrile (150 mL) without any further purification. Propargyl bromide (39.6 mmol, 1 eq) was added dropwise at 80 °C for 30 min, and the reaction was stirred under reflux for 12 h. After cooling to room temperature, acetonitrile was removed under vacuum, and the crude was purified by silica flash chromatography (eluent mixture CHCl_3_/CH_3_OH 8:2) to give a solid product **7**–**9**. For characterization data, see Supplementary Materials.

### 3.2. General Procedure for Nucleobase-Containing 1,5-Disubstituted 1,2,3-Triazoles ***14a***–***25a***

In a 50 mL two-necked round-bottomed flask equipped with a bubble condenser and magnetic stir bar, propargyl nucleobase **7**–**9** (1.52 mmol, 1 eq) was dissolved in DMF (8 mL). Subsequently, FeCl_3_ (0.304 mmol, 0.2 eq) and opportune azide **10**–**13** (3.05 mmol, 2 eq) were added, and the mixture was stirred at 120 °C for 8 h. DMF was removed under vacuum by generating an azeotrope with toluene, and the obtained crude solid was purified on a flash silica gel column (eluent mixture: CHCl_3_/acetone/CH_3_OH 8:1:1 *v*/*v*/*v*) to obtain the desired solid product **14a**–**25a**. The configuration of regioisomers was determined by spectroscopic data reported in Supplementary Materials. Furthermore, to calculate the regioisomeric ratio, the C6H proton on the nucleobases was chosen for both regioisomers (see Supplementary Materials).

### 3.3. Docking Studies

For each receptor, the docking cavity was centered on the binding site of the crystallographic ligand and allowed to extend in a spherical surrounding volume with a radius of 15 Å. In cases where a metal ion was present at the binding site, the docking cavity was centered on it, and metal parameters were set to maintain the same coordination number as that in the crystallographic structure. In the absence of metals, the XYZ coordinates that defined the center of the cavity were obtained from the position of the co-crystalized ligand, choosing an atom that was reasonably at the center of the ligand. The number of genetic algorithm runs was set to 20 for each analyzed ligand. Protein structures were prepared using UCSF Chimera [51], by reverting selenomethionine to methionine, eliminating alternate locations of side chains, adding hydrogen atoms, assigning appropriate protein atom types, and removing the co-crystalized ligand and solvent molecules. Crystallographic ligands were docked after adding hydrogen atoms with UCSF Chimera and without optimizing their geometries. Conversely, the geometries of the screened ligands **14a**–**25a** were optimized quantum mechanically. Geometry optimizations and frequency calculations for stationary point characterization were carried out with Gaussian16 [52] using the M06-2X hybrid functional [53], the 6-31G(d,p) basis set, and ultrafine integration grids. Bulk solvent effects in water were considered implicitly through the IEF-PCM polarizable continuum model [54]. As for the potential receptors, 26 targets were initially selected from the Protein Data Bank (Figure S1). The main selection criterion was the presence of a triazole (i.e., 1,2,3- and 1,2,4-triazoles) scaffold or structurally similar heterocycles (i.e., imidazoles, thiazoles) in the crystallographic structure of the ligand–receptor complex. Docking simulations were performed, keeping the coordinates of the protein fixed while allowing flexibilization of the ligands around their rotatable bonds.

### 3.4. Prediction of Pharmacokinetic Properties

The absorption, distribution, metabolism, and excretion (ADME) analysis and assessment of Lipinski parameters were carried out using the free software “ADMETlab 2.0” supported by the Xiangya School of Pharmaceutical Sciences, Central South University ADMETlab 2.0 (2021) https://admetmesh.scbdd.com (accessed on 12 September 2022) [48]. The main properties considered were physical and chemical properties such as molecular weight, oil/water partition coefficient, and water solubility. Medicinal chemistry parameters were evaluated: the synthetic accessibility score (designed to estimate the ease of synthesis of drug-like molecules) and the violation of Lipinsky’s rules. Absorption was studied mainly considering the permeability after oral administration (Caco-2 cell permeability) and human intestinal absorption (HIA). Among the distribution parameters, Plasma Protein Binding and blood brain–barrier (BBB) penetration were selected. CYP2D6 and CYP3A4 were selected as key enzymes in the metabolism of the compounds. Furthermore, clearance was evaluated as an excretion parameter. Finally, the Brain Or IntestinaL EstimateD permeation method (BOILED-Egg) was used to clarify the main distribution tissue for compounds **14a**–**25a [50]**.

## 4. Conclusions

We have developed a new synthetic approach towards the highly regioselective synthesis of 1,5-disubstituted 1,2,3-triazole derivatives containing pyrimidine nucleobases. *N*1-propargyl nucleobases were first prepared in excellent yields through an alternative route that led only to the *N*1-monoalkylated derivatives. These alkyne compounds were characterized and used in a 1,3-dipolar cycloaddition reaction to obtain the 1,2,3 triazole heterocycles. The 1,5-disubstituted-1,2,3-triazol moiety was obtained in a regioselective way by using FeCl_3_ as an inexpensive and non-toxic catalyst. It was demonstrated that this procedure worked well for different aryl and benzyl azides. Although some differences were found with aryl azides bearing strong electron-withdrawing and electron-donating groups, reaction yields ranged from good to excellent in all cases. Finally, compounds **20a** and **21a** exhibited the best docking scores against four of the selected protein targets, suggesting potential biologic activity for these scaffolds. In addition, ADMET analysis was also performed using an in silico online database, which predicted that all hybrids might have qualified pharmacokinetic parameters and Lipinski’s rule of five properties to claim their intestinal absorption and good clearance rate.

## Figures and Tables

**Table 1:** Optimization of reaction conditions of azide-alkyne 1,3-dipolar cycloaddition.

Entry ^a^	Solvent	Catalyst	T (°C)	Time (h)	Yield (%) ^b^	14a:14b Ratio ^c^
1	CH_2_Cl_2_	Er(OTf)_3_	60	24	-	-
2	THF	Er(OTf)_3_	60	24	-	-
3	THF	Er(OTf)_3_	100	24	15	n.c.
4	CH_3_CN	Er(OTf)_3_	100	24	30	n.c.
5	CH_3_CN	Er(OTf)_3_	120	24	53	80:20
6	CH_3_NO_2_	Er(OTf)_3_	120	24	50	75:25
7	DMF	Er(OTf)_3_	120	8	75	82:18
8	DMF	Yb(OTf)_3_	120	24	70	85:15
9	DMF	ZnCl_2_	120	24	71	77:23
10	DMF	CeCl_3_	120	24	72	80:20
11	DMF	FeCl_3_	120	8	88	88:12
12	DMF	-	120	24	56	63:37
13	Toluene	-	120	24	40	60:40
14	Toluene	FeCl_3_	120	24	48	70:30
15	[mPy](OTf)	FeCl_3_	120	24	-	-

**^a^** Reaction Conditions: **7** (1 eq), catalyst (0.2 eq), **10** (2 eq), in 8 mL of solvent for the appropriate time. **^b^** Isolated yield for regioisomer **14a**. **^c^** Regioisomeric ratio calculated from ^1^H NMR analysis of the reaction crude on the C6H proton of the nucleobase. n.c.= not calculated.

**Table 2:** Substrate scope for the synthesis of 1,5-disubstituted 1,2,3-triazoles **14**–**25**.

Entry ^a^	Nucleobase	R^1^	Azide	R^2^	Product	Yield ^b^(%)	a:b Ratio ^c^
1	**7**	CH_3_	**10**	Ph	**14**	88	88:12
2	**8**	H	**10**	Ph	**15**	90	88:12
3	**9**	F	**10**	Ph	**16**	88	87:13
4	**7**	CH_3_	**11**	Bn	**17**	85	87:13
5	**8**	H	**11**	Bn	**18**	87	88:12
6	**9**	F	**11**	Bn	**19**	84	86:14
7	**7**	CH_3_	**12**	(4-NO_2_)Ph	**20**	83	87:13
8	**8**	H	**12**	(4-NO_2_)Ph	**21**	86	86:14
9	**9**	F	**12**	(4-NO_2_)Ph	**22**	82	85:15
10	**7**	CH_3_	**13**	(4-CH_3_O)Ph	**23**	90	89:11
11	**8**	H	**13**	(4-CH_3_O)Ph	**24**	92	89:11
12	**9**	F	**13**	(4-CH_3_O)Ph	**25**	89	88:12

**^a^** Reaction Conditions: **7** (1 eq), catalyst (0.2 eq), **10** (2 eq), in DMF for the appropriate time. **^b^** Isolated yield for regioisomers 1,5-disubstituted **a**. **^c^** Regioisomeric ratio calculated from ^1^H NMR analysis of the crude on the C6H proton of the nucleobases.

**Table 3:** Pharmacokinetic properties as predicted in silico by the software ADMETlab 2.0.

ADMET	Parameters	14a	15a	16a	17a	18a	19a	20a	21a	22a	23a	24a	25a
**Physio-** **Chemical** **properties**	MW	283.29	269.26	287.25	297.31	283.29	301.28	328.28	314.26	332.25	313.31	299.28	317.28
	Log P **^a^**	0.39	0.044	0.174	0.691	0.368	0.5	0.672	0.286	0.449	0.269	−0.037	0.098
	Log S **^b^**	−2.011	−1.92	−1.97	−2.055	−2.057	−1.98	−2.968	−2.766	−2.845	−2.278	−2.242	−2.208
**Medicinal** **chemistry**	Lipinsky Violation **^c^**	No	No	No	No	No	No	No	No	No	No	No	No
	SAscore **^d^**	2.456	2.475	2.536	2.468	2.485	2.544	2.613	2.628	2.684	2.471	2.485	2.545
**Absorption**	Caco-2 permeability **^e^**	−5.142	−5.444	−5.129	−5.218	−5.573	−5.216	−5.132	−5.409	−5.139	−5.099	−5.287	−5.03
	Pgp- substrate **^f^**	0.003	0.001	0.001	0.002	0.001	0.001	0.005	0.001	0.001	0.006	0.002	0.002
	HIA **^g^**	0.02	0.071	0.021	0.017	0.065	0.15	0.016	0.027	0.023	0.056	0.236	0.053
**Distribution**	BBB permeability **^h^**	0.566	0.691	0.721	0.187	0.323	0.188	0.36	0.386	0.232	0.372	0.679	0.59
	Plasma Protein Binding **^i^**	59.29%	42.51%	49.03%	66.16%	49.75%	58.27%	75.29%	61.81%	68.17%	70.45%	58.58%	60.93%
**Metabolism**	CYP2D6 substrate **^l^**	0.106	0.102	0.088	0.118	0.111	0.101	0.117	0.114	0.097	0.156	0.152	0.123
	CYP3A4 substrate **^l^**	0.403	0.272	0.256	0.354	0.257	0.235	0.24	0.175	0.153	0.457	0.286	0.284
**Excretion**	Clearance **^m^**	7.411	6.579	8.095	8.509	7.408	9.215	7.473	6.571	8.108	7.412	6.665	8.128

**^a^** Log of the octanol/water partition coefficient. Optimal: 0–3. **^b^** Log of the aqueous solubility. Optimal: −4–0.5 log mol/L. **^c^** MW ≤ 500; logP ≤ 5; Hacc ≤ 10; Hdon ≤ 5. **^d^** The synthetic accessibility score is designed to estimate ease of synthesis of drug-like molecules. SAscore ≥ 6, difficult to synthesize; SAscore < 6, easy to synthesize. **^e^** Optimal: higher than −5.15 Log unit. **^f^** The output value is the probability of being the Pgp-substrate. **^g^** Human Intestinal Absorption. Category 1: HIA+(HIA < 30%); Category 0: HIA−(HIA < 30%); The output value is the probability of being HIA+. **^h^** Blood–Brain Barrier Penetration. Category 1: BBB+; Category 0: BBB−; The output value is the probability of being BBB+. **^i^** Optimal: <90%. Drugs with high protein binding may have a low therapeutic index. **^l^** The output value is the probability of being the substrate. **^m^** High: > 15 mL/min/kg; moderate: 5–15 mL/min/kg; low: <5 mL/min/kg.

## Data Availability

Not applicable.

## Supplementary Materials

The following supporting information can be downloaded at https://www.mdpi.com/article/10.3390/molecules27238467/s1, Characterization spectra of the *N*-propargyl nucleobases **7**–**9** (^1^H NMR–^13^C NMR–HRMS); Characterization spectra of nucleobase-containing disubstituted 1,2,3-triazoles **14a, 14b, 15a**–**25a** (^1^H NMR–^13^C NMR–COSY NMR–HRMS); Characterization spectra of derivative **21a** (HMBC and HSQC NMR); Figure S1. Workflow describing the approach used to validate the docking protocol and select the target receptors (first series) and to evaluate the binding capacity of compounds **14a**–**25a** to them.

## Sample Availability

Samples of the compounds are available from the authors.
