# Supplementary material for: Regioselective Synthesis and Molecular Docking Studies of 1,5-Disubstituted 1,2,3-Triazole Derivatives of Pyrimidine Nucleobases
Source: Molecules. 2022 Dec 2;27(23):8467. doi: 10.3390/molecules27238467 (PMC9735522; doi:10.3390/molecules27238467)
Supplement: Supplementary file 1 [file molecules-27-08467-s001.zip › molecules-2045585-supplementary.pdf]

Supplementary Materials

Regioselective Synthesis and Molecular Docking Studies of 1,5-Disubstituted 1,2,3-Triazole Derivatives of Pyrimidine Nucleobases

Vincenzo Algieri [1,*], Paola Costanzo [1,*], Matteo Antonio Tallarida [1,2], Fabrizio Olivito [1], Antonio Jiritano [1], Giulia Fiorani [3], Francesca Peccati [2], Gonzalo Jiménez-Osés [2,4], Loredana Maiuolo [1] and Antonio De Nino [1]

[1] Department of Chemistry and Chemical Technologies, University of Calabria, 87036, Rende, Italy.
[2] Center for Cooperative Research in Biosciences (CIC bioGUNE), Basque Research and Technology Alliance (BRTA), Bizkaia Technology Park, 48160 Derio, Spain
[3] Department Molecular Sciences and Nanosystems, University Ca' Foscari Venezia, Via Torino 155, 30172 Mestre (VE), Italy
[4] Ikerbasque, Basque Foundation for Science, 48013 Bilbao, Spain
* vincenzo.algieri@unical.it (V.A.); paola.costanzo@unical.it (P.C.)

Table of Contents

Materials and Methods	S3
General procedure for nucleobases propargylation and characterization	S3
General procedure for 1,5-disubstituted 1,2,3-triazoles and characterization	S4
Docking Studies	S6
Characterization spectra of the *N*1-propargyl nucleobases **7–9**	S7
Characterization spectra of the Disubstituted 1,2,3-Triazole Derivatives of Pyrimidine Nucleobases **14a, 14b, 15a–25a**	S16

Experimental

Materials and Methods

Commercial starting materials were purchased from Merck (Milano, Italy) or Alfa Aesar (Karlsruhe, Germany) and were used without further purification. Reactions were monitored by TLC using silica plates 60-F264, commercially available from Merck (Milano, Italy). ^{1}H and ^{13}C-NMR specta and two-dimensional NMR spectra were recorded at 300 and 500 MHz and 125.7 MHZ, respectively, in DMSO-d_6 using tetramethylsilane (TMS) as internal standard (Bruker ACP 300 Mhz and Bruker Avance 500 MHz with a 5 mm TBO probe, Rheinstetten, Germany). Chemical shifts are given in parts per million and coupling constants in Hertz. The purity and the regiochemistry were established by NMR spectra (^{1}H-NMR experiments). High-resolution mass spectra (HRMS) were recorded with a Bruker Compact QTOF instrument (Bruker, Billerica, MA, USA). HRMS spectra were acquired in positive ion mode, with a mass resolution of 30000. Mass calibration was performed with a solution of sodium formate clusters and processed in HPC mode. Spectra acquisition was performed in flow injection, with a full scan mode in the range of 50 to 500 m/z. N_2 was the source of dry gas (V = 4 L/min, T= 180 °C). The ion formula of each compound was calculated with the Smart Formula tool of the Bruker software platform, analyzing the isotopic pattern ratio with 4 mDa mass confidence.

General procedure for nucleobases propargylation.

In a three-necked round-bottomed flask, equipped with a bubble condenser and magnetic stir bar, the opportune nucleobase **1–3** (39.6 mmol, 1 eq) in dry hexamethyldisilazane (HMDS, 139 mmol, 3.5 eq) was suspended under nitrogen atmosphere. Subsequently, trimethylsilyl chloride (8.71 mmol, 0.22 eq) and $(NH_4)_2SO_4$ (1.98 mmol, 0.05 eq) were added and the mixture was stirred at 145 °C for 2 h. After completion, the solution was cooled to room temperature and the HMDS was evaporated under vacuum. Then, the obtained silylated nucleobase **4–6** was dissolved in dry acetonitrile (150 mL) without any further purification. Propargyl bromide (39.6 mmol, 1 eq) was added dropwise at 80 °C for 30 min and the reaction was stirred under reflux for 12 h. After cooling to room temperature, acetonitrile was removed under vacuum and the crude was purified by silica flash chromatography (eluent mixture $CHCl_3/CH_3OH$ 8:2) to give a solid product **7–9**.

1-propargylthymine (7). White solid, yield 92%. ($CHCl_3$/acetone/CH_3OH 8:1:1); ^{1}H NMR (300 MHz, DMSO-d_6): δ (ppm)= 1.76 (d, J=1.23 Hz, 3H; CH_3), 3.39 (t, J=2.50 Hz, 1H; C≡CH), 4.46 (d, J= 2.50 Hz, 2H; CH_2), 7.56 (d, J=1.23 Hz, 1H; CH), 11.38 (s, 1H; NH). ^{13}C APT NMR (75 MHz, DMSO-d_6): δ (ppm)= 12.37, 36.77, 76.09, 79.12, 109.86, 140.57, 150.82, 164.59. HRMS (ESI): m/z calcd for $C_8H_9N_2O_2$: 165.0659 $[M+H]^+$, found 165.0653; m/z calcd for $C_8H_8N_2O_2Na$: 187.0483 $[M+Na]^+$, found 187.0473.

1-propargyluracil (8). White solid, yield 90%. ($CHCl_3$/acetone/CH_3OH 8:1:1); ^{1}H NMR (300 MHz, DMSO-d_6): δ (ppm)= 3.43 (t, J=2.50 Hz, 1H; C≡CH), 4.50 (d, J= 2.50 Hz, 2H; CH_2), 5.62 (d, J= 7.89 Hz, 1H; CH), 7.70 (d, J=7.89 Hz, 1H; CH), 11.40 (s, 1H; NH). ^{13}C APT NMR (75 MHz, DMSO-d_6): δ (ppm)= 37.08, 76.29, 78.92, 102.16, 144.24, 150.85, 164.01. HRMS (ESI): m/z calcd for $C_7H_6N_2O_2Na$: 173.0321 $[M+Na]^+$, found 173.0318.

5-fluoro-1-propargyluracil (9). White solid, yield 87%. ($CHCl_3$/acetone/CH_3OH 8:1:1); ^{1}H NMR (300 MHz, DMSO-d_6): δ (ppm)= 3.45 (t, J=2.50 Hz, 1H; C≡CH), 4.45 (d, J= 2.50 Hz, 2H; CH_2), 8.13 (d, J=6.67 Hz, 1H; CH), 11.93 (bd, ^4J=4.39 Hz, 1H; NH). ^{13}C APT NMR (75 MHz, DMSO-d_6): δ (ppm)= 37.46, 76.58, 78.58, 129.38 (d), 140.27 (d), 149.51, 157.80 (d). HRMS (ESI): m/z calcd for $C_7H_5FN_2O_2Na$: 191.0227$[M+Na]^+$, found 191.0223.

*General procedure for 1,5-disubstituted 1,2,3-triazoles **14a-25a**.*

In a 50 ml two-necked round-bottomed flask, equipped with a bubble condenser and magnetic stir bar, propargyl nucleobase **7–9** (1.52 mmol, 1 eq) was dissolved in DMF (8 mL). Subsequently, $FeCl_3$ (0.304 mmol, 0.2 eq) and opportune azide **10–13** (3.05 mmol, 2 eq) were added and the mixture was stirred at 120°C for 8 h. DMF was removed under vacuum by generating an azeotrope with toluene, and the obtained crude solid was purified on a flash silica gel column (eluent mixture: $CHCl_3$/acetone/CH_3OH 8:1:1 v/v/v) to obtain the desired solid products **14a–25a**. The configuration of the regioisomers was attributed by spectroscopic data.

1-[1-phenyl-1,2,3-triazol-5-yl-methyl]-thymine (14a). White solid, yield 88%. ($CHCl_3$/acetone/CH_3OH 8:1:1); ^1H NMR (300 MHz, DMSO-d$_6$): δ (ppm)= 1.77 (s, 3H; CH_3), 5.00 (s, 2H; CH_2), 7.43-7.53 (m, 1H; Ar), 7.58 (t, *J*=7.83 Hz, 2H; Ar), 7.67 (s, 1H; CH), 7.89 (d, *J*=7.83 Hz, 2H; Ar), 8.79 (s, 1H, CH), 11.37 (s, 1H; NH). ^{13}C APT NMR (75 MHz, DMSO-d$_6$): δ (ppm)= 12.16, 42.35, 109.21, 120.23, 121.87, 128.89, 130.04, 136.70, 141.28, 144.03, 150.96, 164,51. HRMS (ESI): *m/z* calcd for $C_{14}H_{14}N_5O_2$: 284.1142 $[M+H]^+$, found 284.1142; *m/z* calcd for $C_{14}H_{13}N_5O_2Na$: 306.0967 $[M+Na]^+$, found 306.0960; *m/z* calcd for $C_{14}H_{13}N_5O_2K$: 322.0706 $[M+K]^+$, found 322.0699.

1-[(1-phenyl-1,2,3-triazol-4-yl)-methyl]-thymine (14b). White solid. ($CHCl_3$/acetone/CH_3OH 8:1:1); ^1H-NMR (300 MHz, DMSO-*d$_6$*,): *δ* (ppm)= 1.66 (s, 3H, CH_3), 5.00 (s, 2H, CH_2), 7.32 (s, 1H, CH), 7.41-7.75 (m, 5H, Ar), 7.80 (s, 1H, CH), 11.32 (s, 1H, NH). ^{13}C-APT NMR (75 MHz, DMSO-*d$_6$*): *δ* (ppm) 11.92, 40.91, 109.10, 125.30, 129.72, 129.96, 133.16, 134.49, 135.59, 140.54, 150.55, 164.13.

1-[1-phenyl-1,2,3-triazol-5-yl-methyl]-uracil (15a). White solid, yield 90%. ($CHCl_3$/acetone/CH_3OH 8:1:1); ^1H NMR (300 MHz, DMSO-*d$_6$*): *δ* (ppm)= 5.04 (s, 2H; CH_2), 5.62 (d, *J*=7.80 Hz, 1H; CH), 7.43-7.54 (m, 1H; Ar), 7.54-7.67 (m, 2H; Ar), 7.80 (d, *J*=7.80 Hz, 1H; CH), 7.85-7.96 (m, 2H; Ar), 8.80, (s, 1H; CH), 11.36 (s, 1H; NH). ^{13}C APT NMR (75 MHz, DMSO-*d$_6$*): *δ* (ppm)= 42.54, 101.59, 120.24, 121.88, 128.90, 130.04, 136.68, 143.85, 145.63, 150.96, 163.90. HRMS (ESI): *m/z* calcd for $C_{13}H_{12}N_5O_2$: 270.0986 $[M+H]^+$, found 270.0986; *m/z* calcd for $C_{13}H_{11}N_5O_2Na$: 292.0810 $[M+Na]^+$, found 292.0805; *m/z* calcd for $C_{13}H_{11}N_5O_2K$: 308.0550 $[M+K]^+$, found 308.0544.

5-fluoro-1-[1-phenyl-1,2,3-triazol-5-yl-methyl]-uracil (16a). White solid, yield 88%. ($CHCl_3$/acetone/CH_3OH 8:1:1); ^1H NMR (300 MHz, DMSO-*d$_6$*): *δ* (ppm)= 5.01 (s, 2H; CH_2), 7.43-7.54 (m, 1H; Ar), 7.59 (t, *J*=7.97 Hz, 2H; Ar), 7.89 (d, *J*=7.97 Hz, 2H; Ar), 8.24 (d, *J*=6.70 Hz, 1H; CH), 8.80 (s, 1H; CH), 11.91 (s, 1H; NH). ^{13}C APT NMR (75 MHz, DMSO-*d$_6$*): *δ* (ppm)= 43.24, 120.51, 122.17, 129.22, 130.35, 130.36 (d, *J*=33.70 Hz), 136.99, 140.31 (d, *J*=229.50 Hz), 144.05, 149.94, 158.02 (d, 25.84 Hz). HRMS (ESI): *m/z* calcd for $C_{13}H_{11}FN_5O_2$: 288.0891 $[M+H]^+$, found 288.0888; *m/z* calcd for $C_{13}H_{10}FN_5O_2Na$: 310.0716 $[M+Na]^+$, found 310.0706; *m/z* calcd for $C_{13}H_{10}FN_5O_2K$: 326.0456 $[M+K]^+$, found 326.0445.

1-[1-benzyl-1,2,3-triazol-5-yl-methyl]-thymine (17a). White solid, yield 85%. ($CHCl_3$/acetone/CH_3OH 8:1:1); ^1H NMR (300 MHz, DMSO-*d$_6$*): *δ* (ppm)= 1.75 (s, 3H; CH_3), 4.89 (s, 2H; CH_2), 5.57 (s, 2H; CH_2), 7.24-7.44 (m, 5H; Ar), 7.63 (s, 1H; CH), 8.14 (s, 1H; CH), 11.33 (s, 1H; NH). ^{13}C APT NMR (75 MHz, DMSO-*d$_6$*): *δ* (ppm)= 17.01, 47.39, 57.94, 113.96, 128.71, 133.10, 133.26, 133.85, 141.02, 146.27, 147.93, 155.81, 169.36. HRMS (ESI): *m/z* calcd for $C_{15}H_{16}N_5O_2$: 298.1299 $[M+H]^+$, found 298.1293.

1-[1-benzyl-1,2,3-triazol-5-yl-methyl]-uracil (18a). White solid, yield 87%. ($CHCl_3$/acetone/CH_3OH 8:1:1); ^1H NMR (300 MHz, DMSO-*d$_6$*): *δ* (ppm)= 4.92 (s, 2H; CH_2), 5.58 (d, 1H, *J*=7.5 Hz; CH), 7.27-7.40 (m, 5H; Ar), 7.75 (d, *J*=7.5 Hz, 1H; CH), 8.14 (s, 1H; CH), 11.33 (s, 1H; NH). ^{13}C APT NMR (75 MHz, DMSO-*d$_6$*): *δ* (ppm)= 42.90, 53.28, 101.70, 124.10, 128.46, 128.62, 129.21, 136.38, 143.14, 145.96, 151.19, 164.13. HRMS (ESI): *m/z* calcd for $C_{14}H_{14}N_5O_2$: 284.1142 $[M+H]^+$, found 284.1138; *m/z* calcd for $C_{14}H_{13}N_5O_2Na$: 306.0967 $[M+Na]^+$, found 306.0957.

5-fluoro-1-[1-benzyl-1,2,3-triazol-5-yl-methyl]-uracil (19a). White solid, yield 84%. ($CHCl_3$/acetone/CH_3OH 8:1:1); ^1H NMR (300 MHz, DMSO-*d$_6$*): *δ* (ppm)= 4.90 (s, 2H; CH_2), 5.58 (s, 2H; CH_2), 7.29-7.34 (m, 3H; Ar), 7.34-7.39 (m, 2H; Ar), 8.16 (s, 1H; CH), 8.18 (d, *J*=6.75 Hz, 1H; CH), 11.63 (s, 1H; NH). ^{13}C NMR (75 MHz, DMSO-*d$_6$*): *δ* (ppm)= 43.30, 53.35, 124.16, 128.49, 128.64, 129.23, 136.37, 139.24, 141.06, 142.96, 149.87, 157.93. HRMS

(ESI): *m/z* calcd for $C_{14}H_{13}FN_5O_2$: 302.1048 $[M+H]^+$, found 302.1038; *m/z* calcd for $C_{14}H_{12}FN_5O_2Na$: 324.0873 $[M+Na]^+$, found 324.0856.

1-[1-(4-nitrophenyl)-1,2,3-triazol-5-yl-methyl]-thymine (20a). Pale-yellow solid, yield 83%. ($CHCl_3$/acetone/CH_3OH 8:1:1); ^1H NMR (300 MHz, DMSO-*d6*): δ (ppm)= 1.77 (s, 3H; CH_3), 5.03 (s, 2H; CH_2), 7.68 (s, 1H; CH), 8.22 (d, *J*=9.01 Hz, 2H; Ar), 8.44 (d, 2H, *J*=9.01 Hz; Ar), 9.00 (s, 1H; CH), 11.39 (s, 1H; NH). ^{13}C NMR (125 MHz, DMSO-*d6*): δ (ppm)= 12.55, 42.77, 109.74, 121.25, 122.75, 126.12, 141.68, 141.72, 145.11, 147.35, 151.37, 164.98. HRMS (ESI): *m/z* calcd for $C_{14}H_{12}N_6$ O_4Na: 351.0812 $[M+Na]^+$, found 351.0812.

1-[1-(4-nitrophenyl)-1,2,3-triazol-5-yl-methyl]-uracil (21a). Pale-yellow solid, yield 86%. ($CHCl_3$/acetone/CH_3OH 8:1:1); ^1H NMR (300 MHz, DMSO-*d6*): δ (ppm)= 5.09 (s, 2H; CH_2), 5.66 (d, 1H, J=7.84 Hz, CH), 7.83 (d, 1H, J=7.84 Hz, CH), 8.24 (d, 2H, J=8.78 Hz, Ar), 8.45 (d, 2H, J=8.78 Hz, Ar), 9.02 (s, 1H, CH), 11.39 (s, 1H, NH). ^{13}C NMR (125 MHz, DMSO-*d6*): δ (ppm)= 42.94, 102.04, 121.17, 122.72, 126.06, 141.25, 144.92, 146.01, 147.29, 151.34, 164.34. HRMS (ESI): *m/z* calcd for $C_{13}H_{10}N_6O_4Na$: 337.0656 $[M+Na]^+$, found 337.0653.

5-fluoro-1-[1-(4-nitrophenyl)-1,2,3-triazol-5-yl-methyl]-uracil (22a). Pale-yellow solid, yield 82%. ($CHCl_3$/acetone/CH_3OH 8:1:1); ^1H NMR (300 MHz, DMSO-*d6*): δ (ppm)= 5.07 (s, 2H; CH_2), 7.98 (m, 2H; Ar), 8.06 (d, *J*= 6.75 Hz, 1H; CH), 8.47 (m, 2H; Ar), 9.02 (s, 1H; CH), 11.90 (s, 1H; NH). ^{13}C NMR (125 MHz, DMSO-*d6*): δ (ppm)= 43.36, 121.24, 126.92, 135.31, 141.07, 141.31, 144.85, 147.39, 148.38, 150.03, 158.20. HRMS (ESI): *m/z* calcd for $C_{13}H_{10}FN_6O_4$: 333.0742 $[M+H]^+$, found 333.0733; *m/z* calcd for $C_{13}H_9FN_6O_4Na$: 355.0567 $[M+Na]^+$, found 355.0554; *m/z* $[M+K]^+$ calcd for $C_{13}H_9FN_6O_4K$: 371.0306 $[M+K]^+$, found 371.0293.

1-[1-(4-methoxyphenyl)-1,2,3-triazol-5-yl-methyl]-thymine (23a). White solid, yield 89%. ($CHCl_3$/acetone/CH_3OH 8:1:1); ^1H NMR (300 MHz, DMSO-*d6*): δ (ppm)= 1.77 (s, 3H; CH_3), 3.82 (s, 3H; CH_3), 4.98 (s, 2H; CH_2), 7.12 (d, 2H, *J*=9.05 Hz; Ar), 7.66 (s, 1H; CH), 7.79 (d, 2H, *J*=9.05 Hz; Ar), 8.67 (s, 1H; CH), 11.35 (s, 1H; NH). ^{13}C NMR (125 MHz, DMSO-*d6*): δ (ppm)= 12.45, 42.63, 56.04, 109.47, 115.32, 122.12, 122.21, 130.43, 141.58, 144.04, 151.24, 159.77, 164.80. HRMS (ESI): *m/z* calcd for $C_{15}H_{16}N_5O_3$: 314.1248 $[M+H]^+$, found 314.1245; *m/z* calcd for $C_{15}H_{15}N_5O_3Na$: 336.1073 $[M+Na]^+$, found 336.1062; *m/z* calcd for $C_{15}H_{15}N_5O_3K$: 352.0812 $[M+K]^+$, found 352.0805.

1-[1-(4-methoxyphenyl)-1,2,3-triazol-5-yl-methyl]-uracil (24a). White solid, yield 90%. ($CHCl_3$/acetone/CH_3OH 8:1:1); ^1H NMR (300 MHz, DMSO-*d6*): δ (ppm)=3.82 (s, 3H; CH_3), 5.02 (s, 2H; CH_2), 5.62 (d, 1H, *J*=7.89 Hz; CH), 7.12 (m, 2H; Ar), 7.79 (m, 2H; Ar), 7.81 (d, 1H, *J*=7.89 Hz, CH), 8.69 (s, 1H; CH), 11.37 (s, 1H; NH). ^{13}C NMR (125 MHz, DMSO-*d6*): δ (ppm)= 42.83, 56.02, 101.86, 115.31, 122.07, 122.20, 130.41, 143.87, 145.93, 151.26, 159.77, 164.21. HRMS (ESI): *m/z* calcd for $C_{14}H_{14}N_5O_3$: 300.1091 $[M+H]^+$, found 300.1086; *m/z* calcd for $C_{14}H_{13}N_5O_3Na$: 322.0916 $[M+Na]^+$, found 322.0903; calcd for $C_{14}H_{13}N_5O_3K$: 338.0655 *m/z* $[M+K]^+$, found 338.0642.

5-fluoro-1-[1-(4-methoxyphenyl)-1,2,3-triazol-5-yl-methyl]-uracil (25a). White solid, yield 88%. ($CHCl_3$/acetone/CH_3OH 8:1:1); ^1H NMR (300 MHz, DMSO-*d6*): δ (ppm)=3.82 (s, 3H; CH_3), 4.99 (s, 2H; CH_2), 7.12 (m, 2H; Ar), 7.79 (m, 2H; Ar), 8.24 (d, *J*= 6.70 Hz, 1H; CH), 8.70 (s, 1H; CH), 11.91 (d, *J*= 6.67 Hz, 1H; NH). ^{13}C NMR (125 MHz, DMSO-*d6*): δ (ppm)= 43.24, 56.04, 115.33, 122.17, 130.38, 130.43, 139.38, 141.21, 143.77,149.94, 158.03, 159.79. HRMS (ESI): *m/z* calcd for $C_{14}H_{12}FN_5$ O_3Na: 340.0816$[M+Na]^+$, found 340.0809.

Docking studies

For each receptor, the docking cavity was centered on the binding site of the crystalographic ligand and allowed to extend in a spherical surrounding volume with a radius of 15 Å radius. In cases where a metal ion was present at the binding site, the docking cavity was centered on it and metal parameters were set to maintain the same coordination number as in the crystallographic structure. In absence of metals, the XYZ coordinates that defined the center of the cavity were obtained from the position of the cocrystallized ligand, choosing an atom which was reasonably at the center of the ligand. The number of genetic algorithm runs was set to 20 for each analyzed ligand. Protein structures were prepared using UCSF Chimera,[1] by reverting selenomethionine to methionine, eliminating alternate locations of side chains, adding hydrogen atoms, assinging appropiate protein atom types, and removing the co-crystalyzed ligand and solvent molecules. Crystallographic ligands were docked after adding hydrogen atoms with UCSF Chimera and without optimizing their geometries. Conversely, the geometries of the screened ligands **14a–25a** were optimized quantum mechanically. Geometry optimizations and frequency calculations for stationary point characterization were carried out with Gaussian16[2] using the M06-2X hybrid functional,[3] the 6-31G(d,p) basis set and ultrafine integration grids. Bulk solvent effects in water were considered implicitly through the IEF-PCM polarizable continuum model.[4] As for the potential receptors, 26 targets were initially selected from the Protein Data Bank (Figure S1). The main selection criterion was the presence of a triazole (i.e. 1,2,3- and 1,2,4-triazoles) scaffold or structurally similar heterocycles (i.e. imidazoles, thiazoles) in the crystallographic structure of the ligand-receptor complex. Docking simulations were performed and keeping the coordinates of the protein fixed while allowing flexibilization of the ligands around their rotatable bonds.

[1] Pettersen, E.F.; Goddard, T.D.; Huang, C.C.; Couch, G.S.; Greenblatt, D.M.; Meng, E.C.; Ferrin. T.E. UCSF Chimera—A Visualization System for Exploratory Research and Analysis. *J. Comput. Chem*. **2004**, *25*, 1605–1612.

[2] Frisch, M.J.; Trucks, G.W.; Schlegel, H.B.; Scuseria, G.E.; Robb, M.A.; Cheeseman, J.R.; Scalmani, G.; Barone, V.; Mennucci, B.; Petersson, G.A.; et al. *Gaussian 16, Revision C.01*; Gaussian, Inc.: Wallingford, CT, USA, 2016.

[3] Zhao, Y.; Truhlar, D.G. The M06 suite of density functionals for main group thermochemistry, thermochemical kinetics, noncovalent interactions, excited states, and transition elements: Two new functionals and systematic testing of four M06-class functionals and 12 other functionals. *Theor. Chem. Acc*. **2008**, *120*, 215–241.

[4] Scalmani, G.; Frisch. M.J. Continuous surface charge polarizable continuum models of solvation. I. General formalism. *J. Chem. Phys.* **2010**, *132*, 114110.

First series

PDB	
7OVE	7AOP
3SFY	7NNG
6ECL	6GY9
7FD0	7LAF
7B7B	7MLS
6TLU	7NQ5
6LP6	6TKS
6X2J	6JUT
5UI1	5WNF
5OKT	6AU3
5XYY	5LJT
5L7I	5E13
5A3U	4URA

Natural ligand docking

Second series[a]

PDB	
7OVE	7AOP
3SFY	7NNG
7LAF	6GY9
7FD0	5E13
6TLU	6LP6
6TKS	6JUT
6X2J	5UI1
6AU3	5XYY

[a]RMSD values lower than 1.5 Å

Final results[b]

PDB	Natural ligand		Best compound score
	Score	RMSD (Å)	
7OVE	34.5	1.241	36.7 (**21**)
7NNG	22.1	0.685	22.3 (**21**)
6GY9	21.3	1.360	21.7 (**21**)
5E13	16.7	0.768	26.8 (**20**)
6TLU	26.4	0.665	23.1 (**17**)
6AU3	31.0	0.625	28.8 (**23**)
5XYY	28.6	0.450	28.5 (**14**)

[b]Of all the docking calculations performed in the second series, only those cases where our compounds showed a docking score better or similar to the docking score of the natural ligand were considered.

Figure S1. Workflow describing the approach used to validate the docking protocol and select the target receptors (first series) and to evaluate the binding capacity of compounds **14a**-**25a** to them.

Characterization spectra of the *N*-propargyl nucleobases

1-propargylthymine (7).

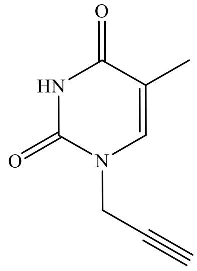

^{1}H NMR

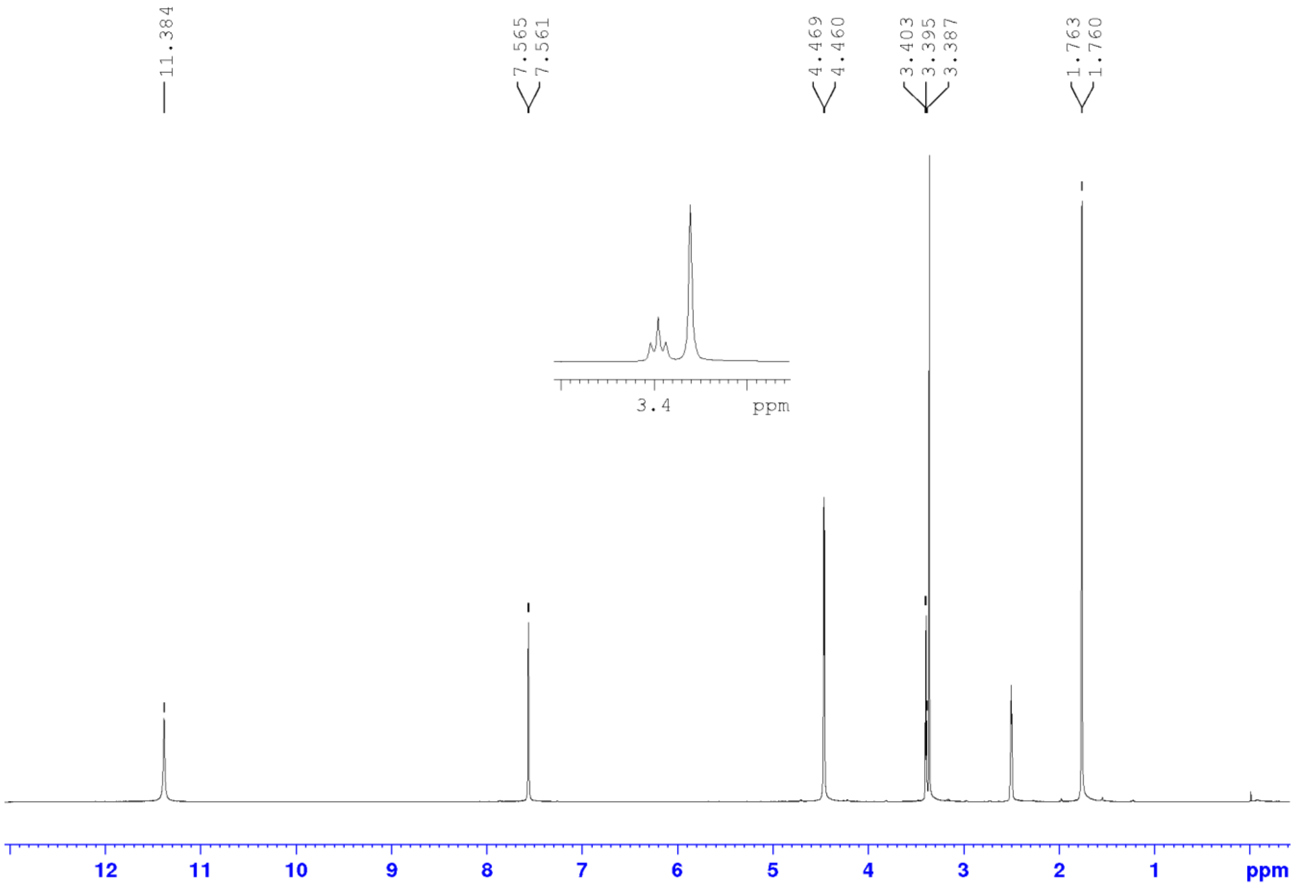

^{13}C NMR

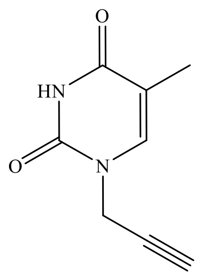

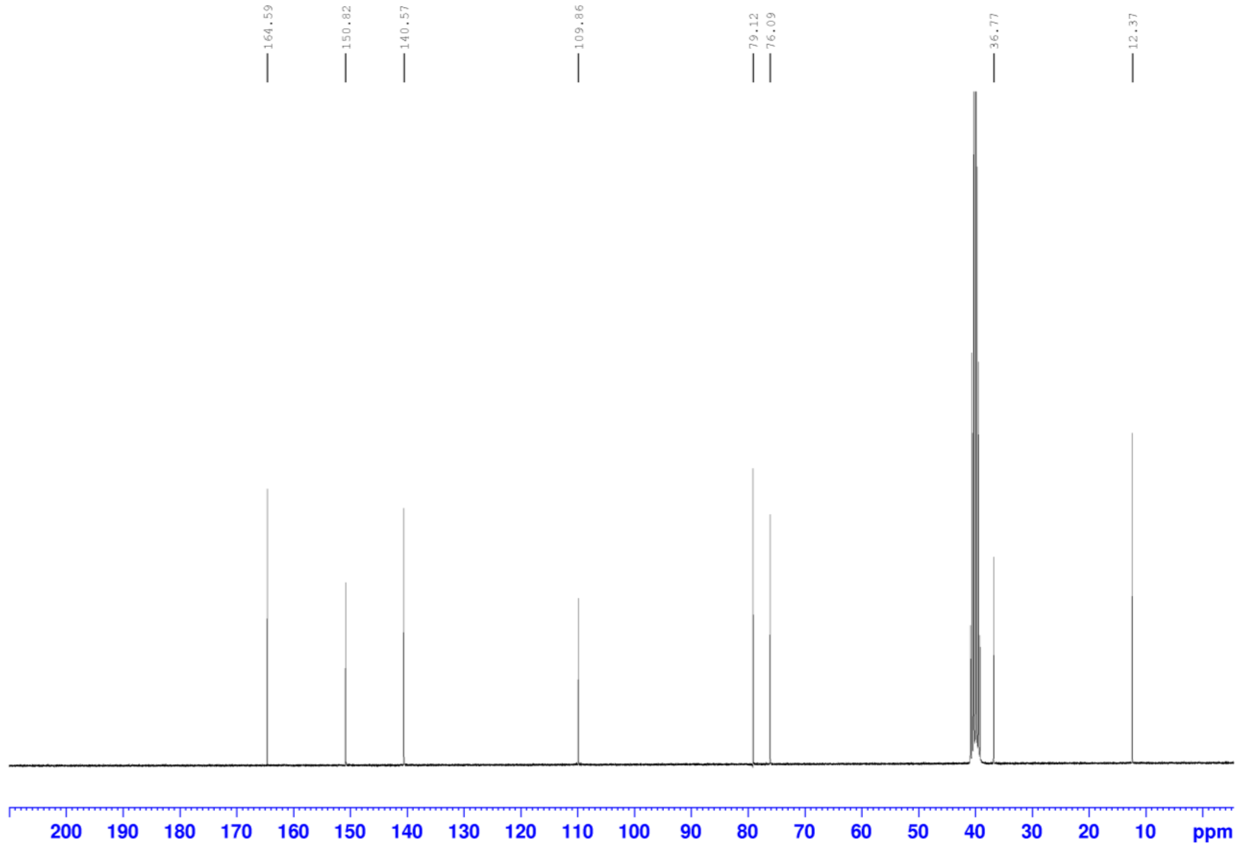
164.59
150.82
140.57
109.86
79.12
76.09
36.77
12.37
200 190 180 170 160 150 140 130 120 110 100 90 80 70 60 50 40 30 20 10 ppm

HRMS

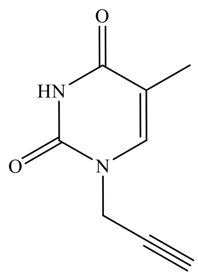
O
HN
O
N

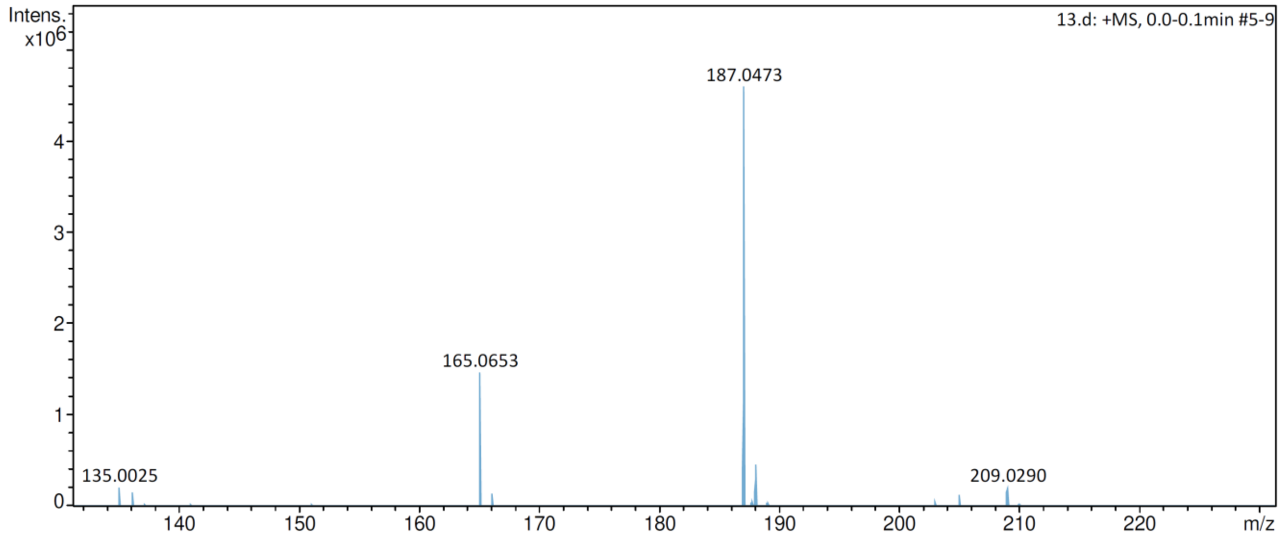
Intens.
x10^6
13.d: +MS, 0.0-0.1min #5-9
187.0473
165.0653
135.0025
209.0290
4
3
2
1
0
140
150
160
170
180
190
200
210
220
m/z

1-propargyluracil (8)

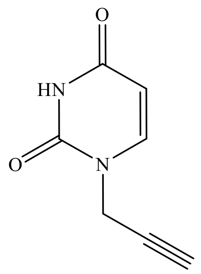

^{1}H NMR

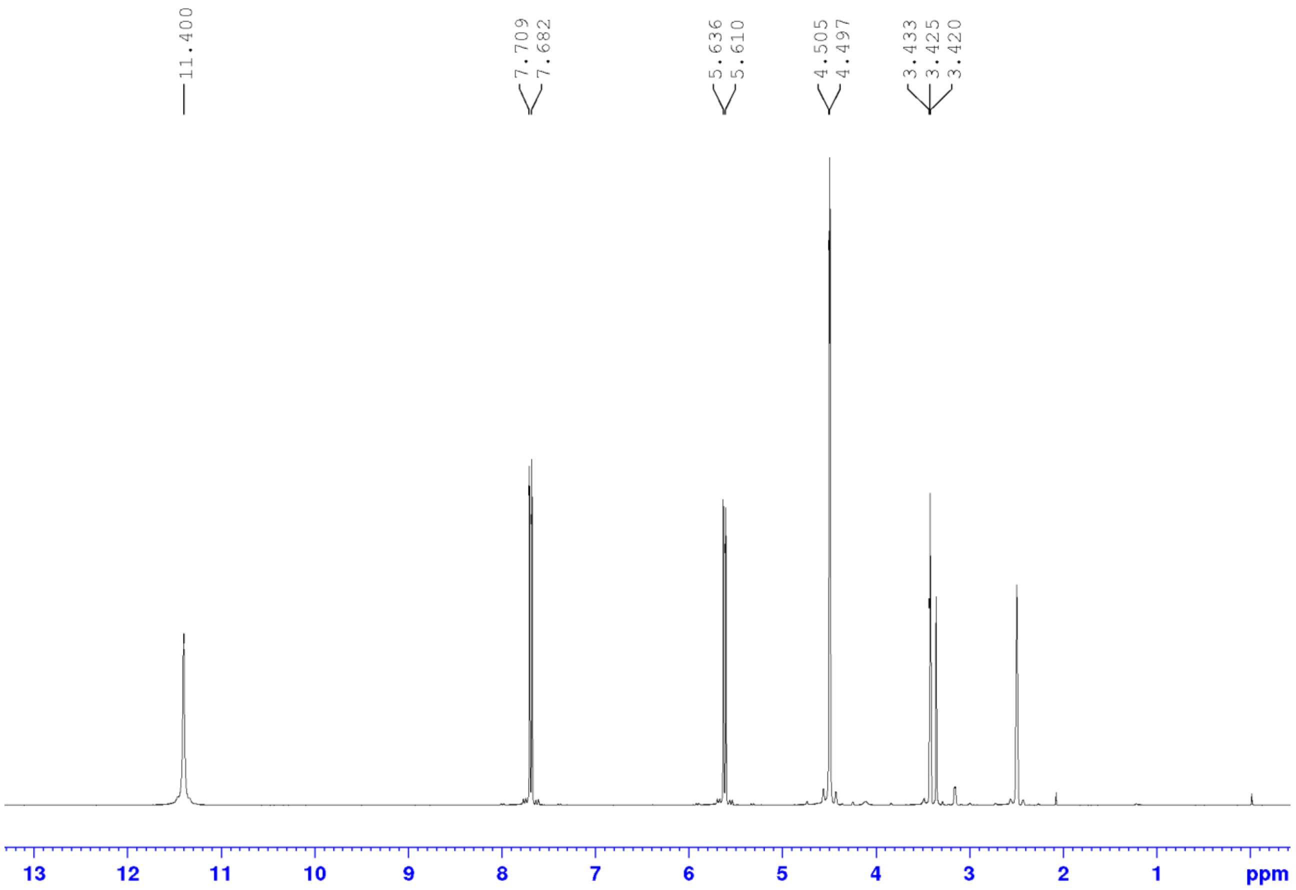

^{13}C-APT NMR

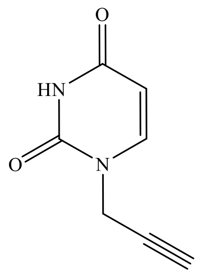

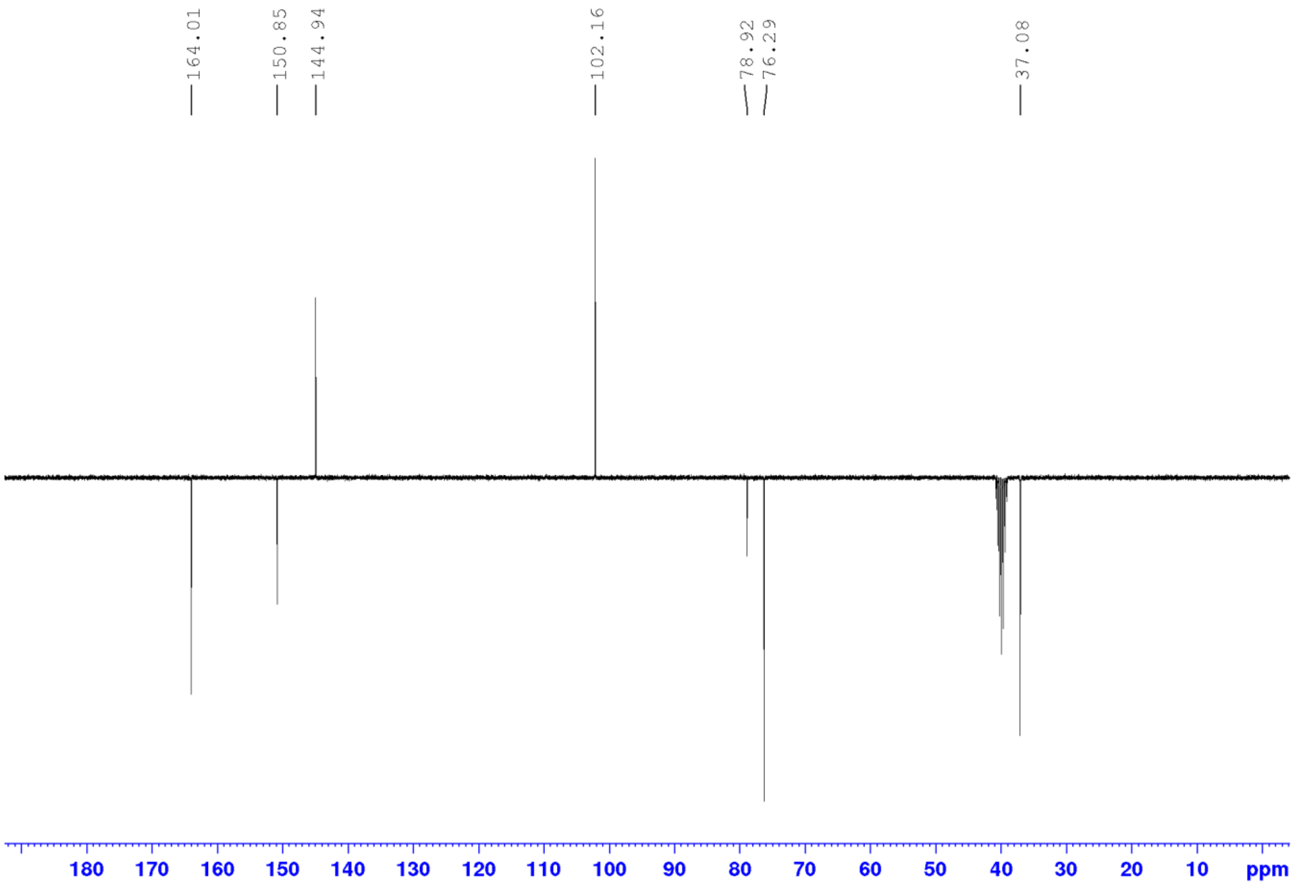
164.01
150.85
144.94
102.16
78.92
76.29
37.08
180 170 160 150 140 130 120 110 100 90 80 70 60 50 40 30 20 10 ppm

HRMS

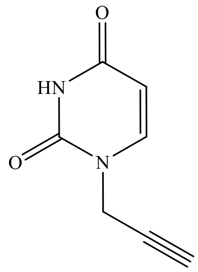
O
HN
O
N

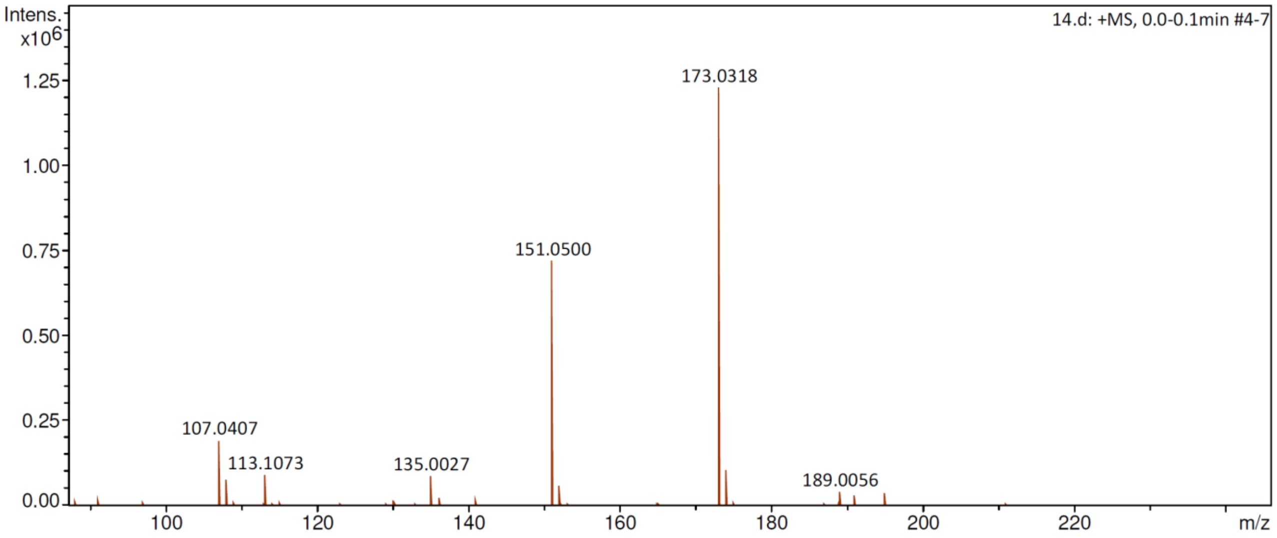
Intens.
x10^6
14.d: +MS, 0.0-0.1min #4-7
173.0318
151.0500
107.0407
113.1073
135.0027
189.0056
1.25
1.00
0.75
0.50
0.25
0.00
100
120
140
160
180
200
220
m/z

5-fluoro-1-propargyluracil (9).

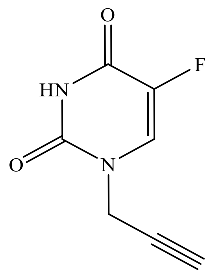

^{1}H NMR

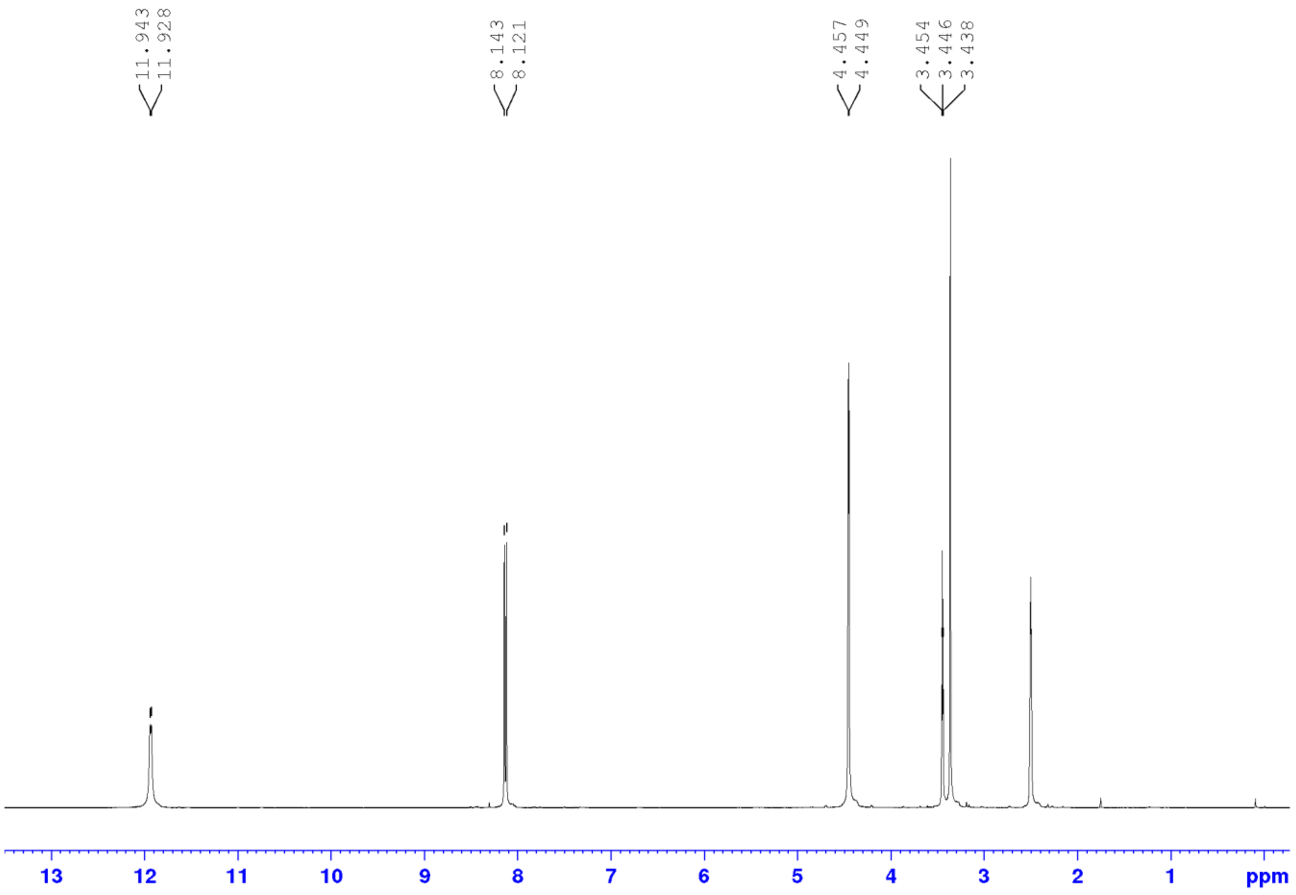

13C-APT NMR

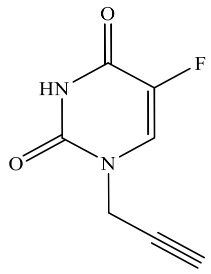
O
F
HN
O
N

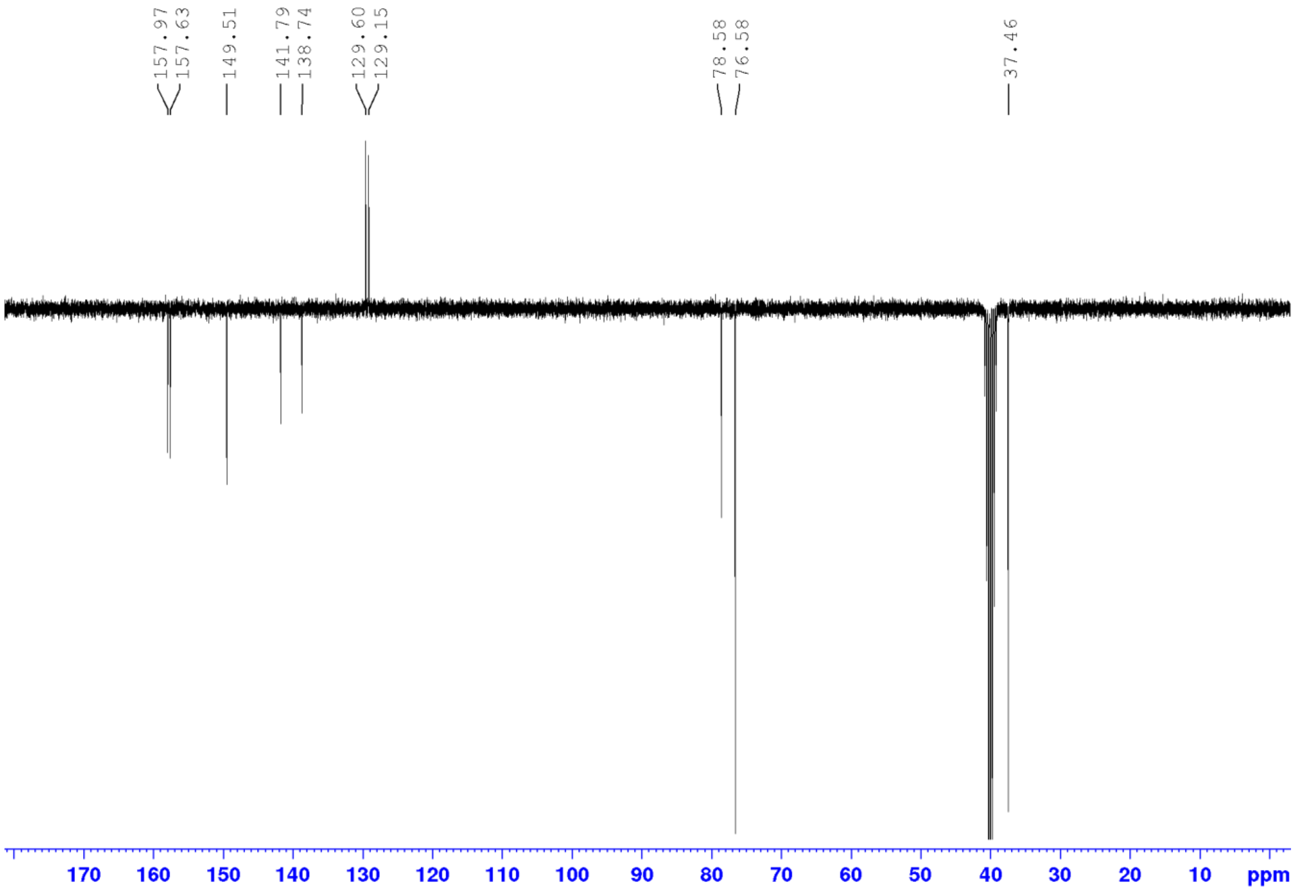
157.97
157.63
149.51
141.79
138.74
129.60
129.15
78.58
76.58
37.46
170
160
150
140
130
120
110
100
90
80
70
60
50
40
30
20
10
ppm

HRMS

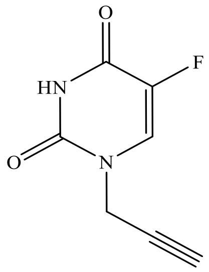
O
F
HN
O
N

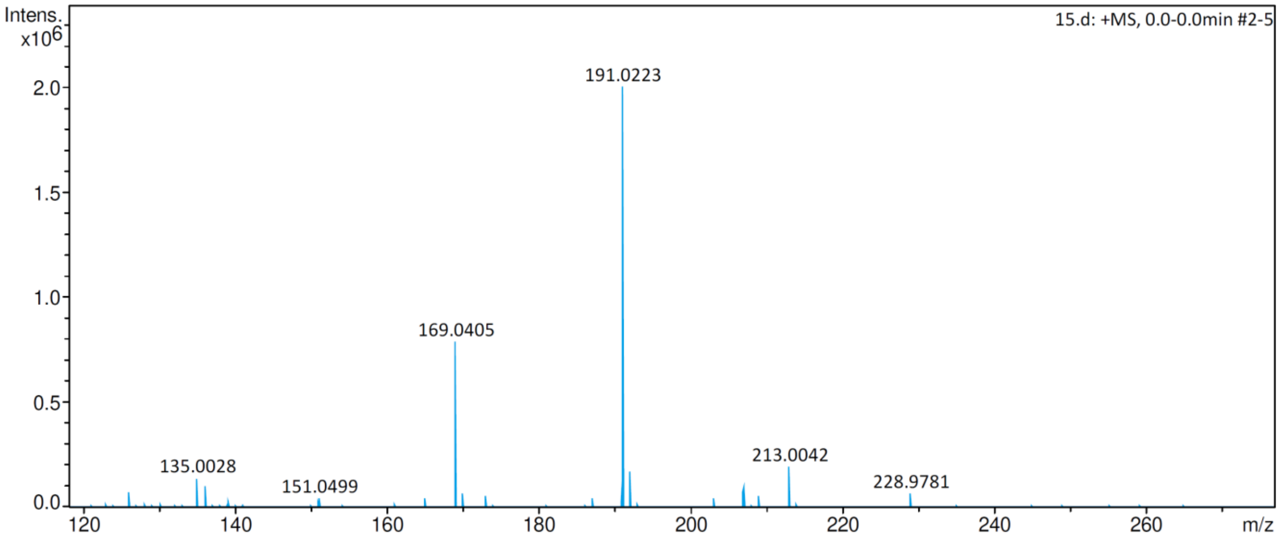
Intens.
x10^6
15.d: +MS, 0.0-0.0min #2-5
191.0223
169.0405
213.0042
135.0028
151.0499
228.9781
2.0
1.5
1.0
0.5
0.0
120
140
160
180
200
220
240
260
m/z

1,5-Disubstituted 1,2,3-Triazole Derivatives of Pyrimidine Nucleobases

1-[1-phenyl-1,2,3-triazol-5-yl-methyl]-thymine (14a).

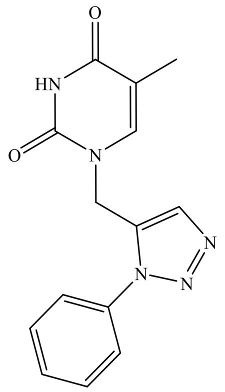

1H NMR

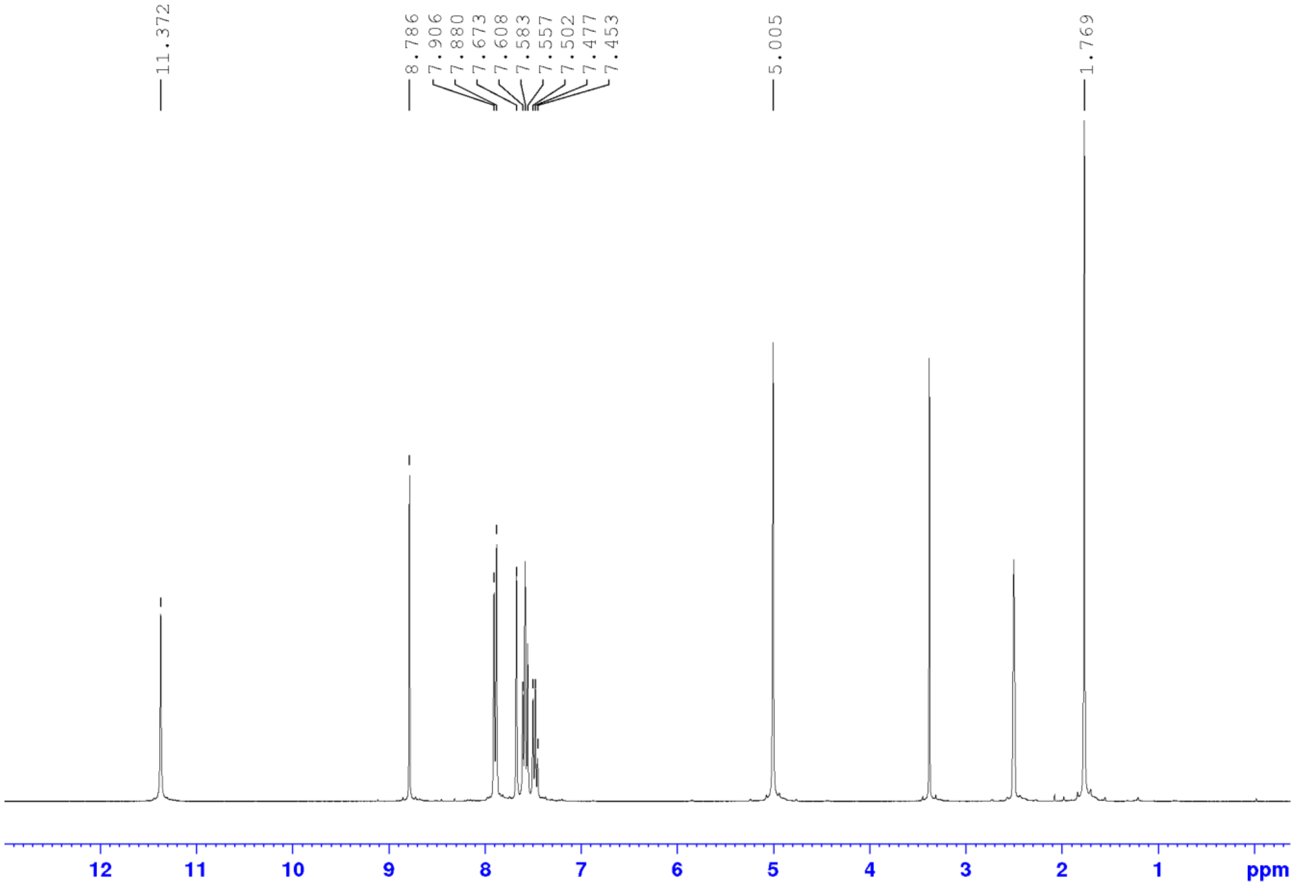

^{13}C-APT NMR

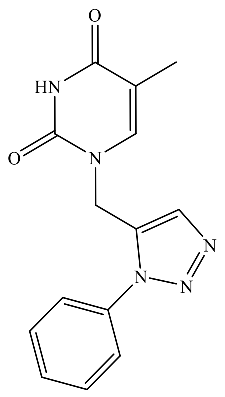
O
HN
O
N
N
N
N

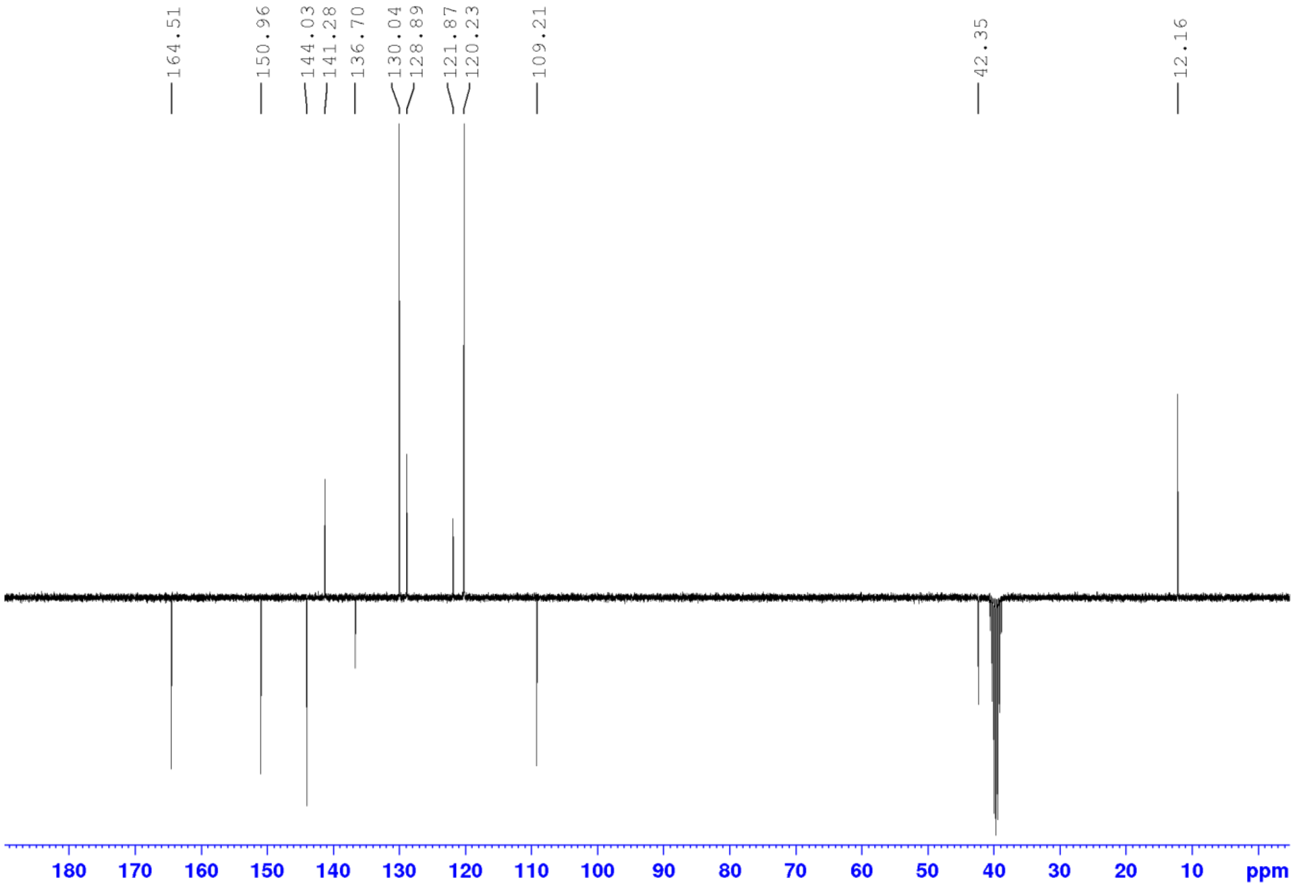
164.51
150.96
144.03
141.28
136.70
130.04
128.89
121.87
120.23
109.21
42.35
12.16
180
170
160
150
140
130
120
110
100
90
80
70
60
50
40
30
20
10
ppm

COSY NMR

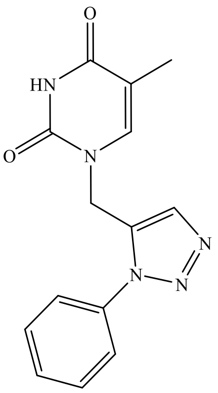

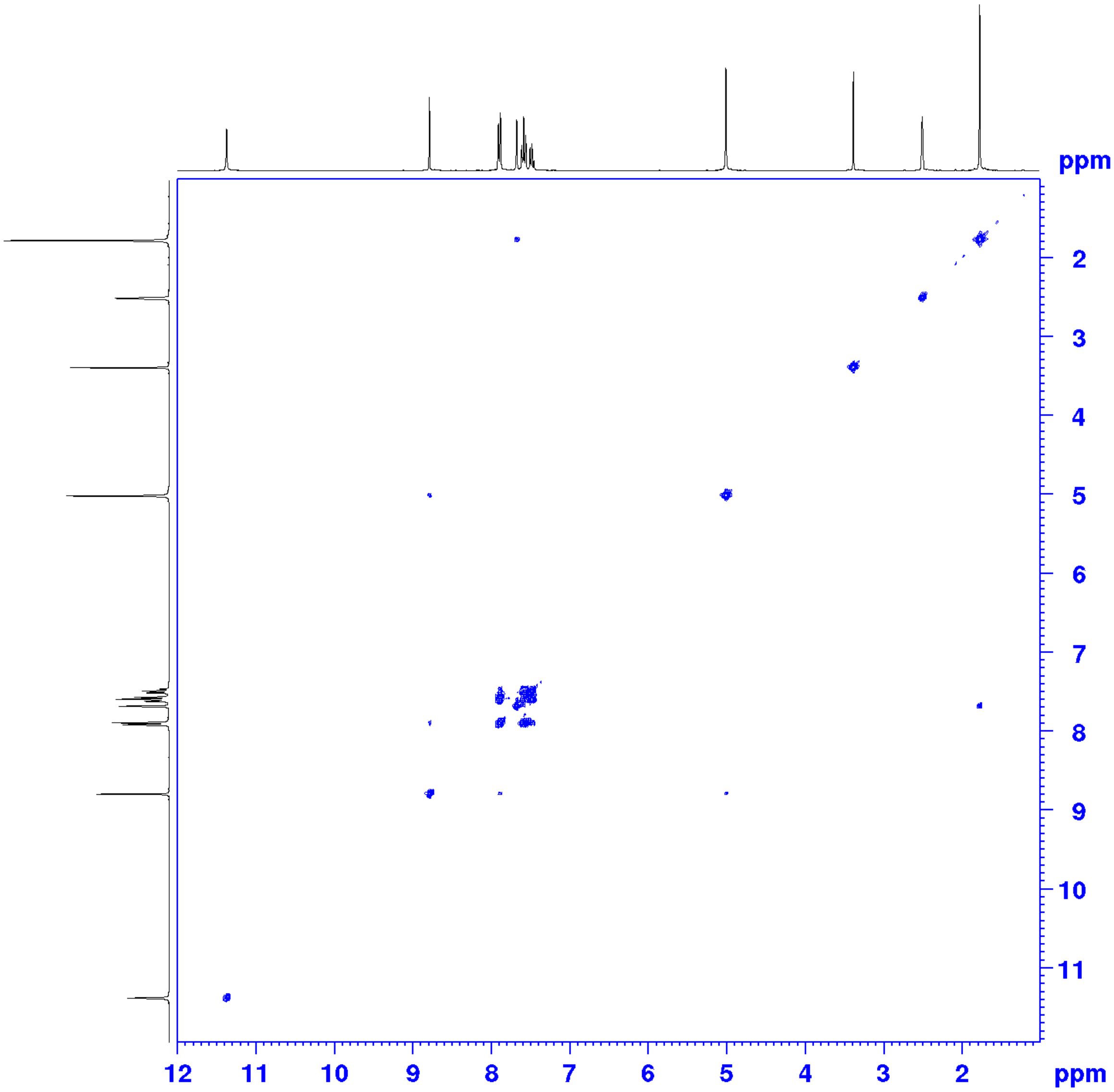

HRMS

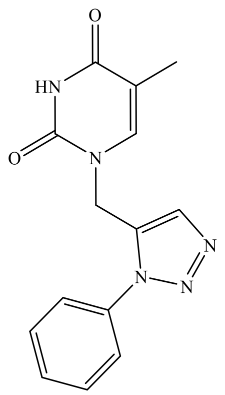

O
HN
O
N
N
N
N

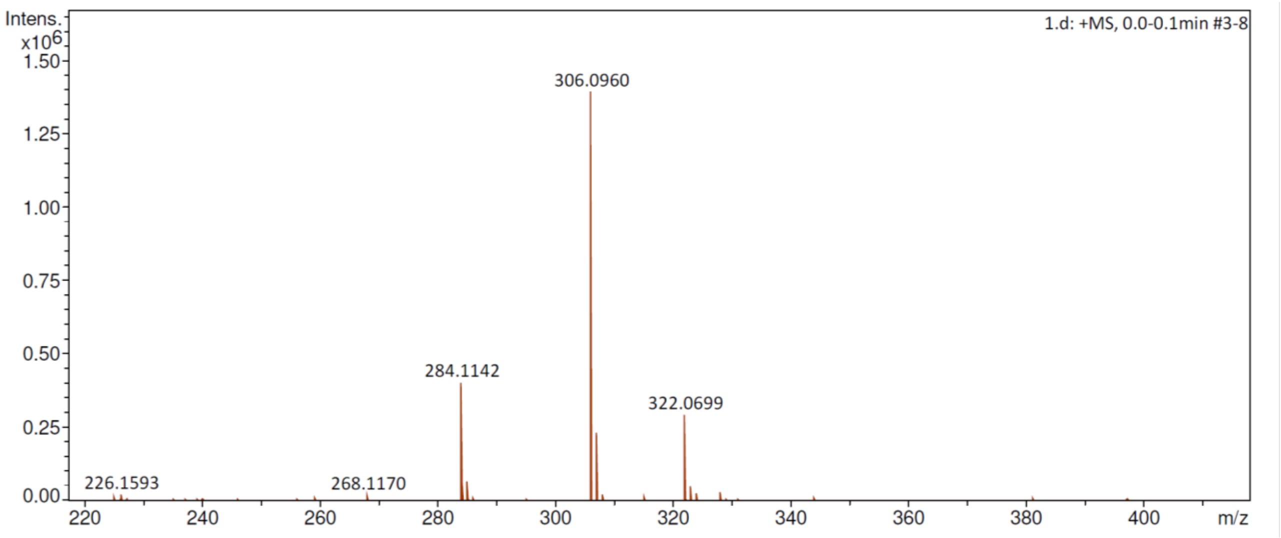

Intens.
x10^6
1.d: +MS, 0.0-0.1min #3-8
306.0960
284.1142
322.0699
226.1593
268.1170
m/z

^{1}H NMR of reaction crude for the synthesis of 1-[1-phenyl-1,2,3-triazol-5-yl-methyl]-thymine (14a)

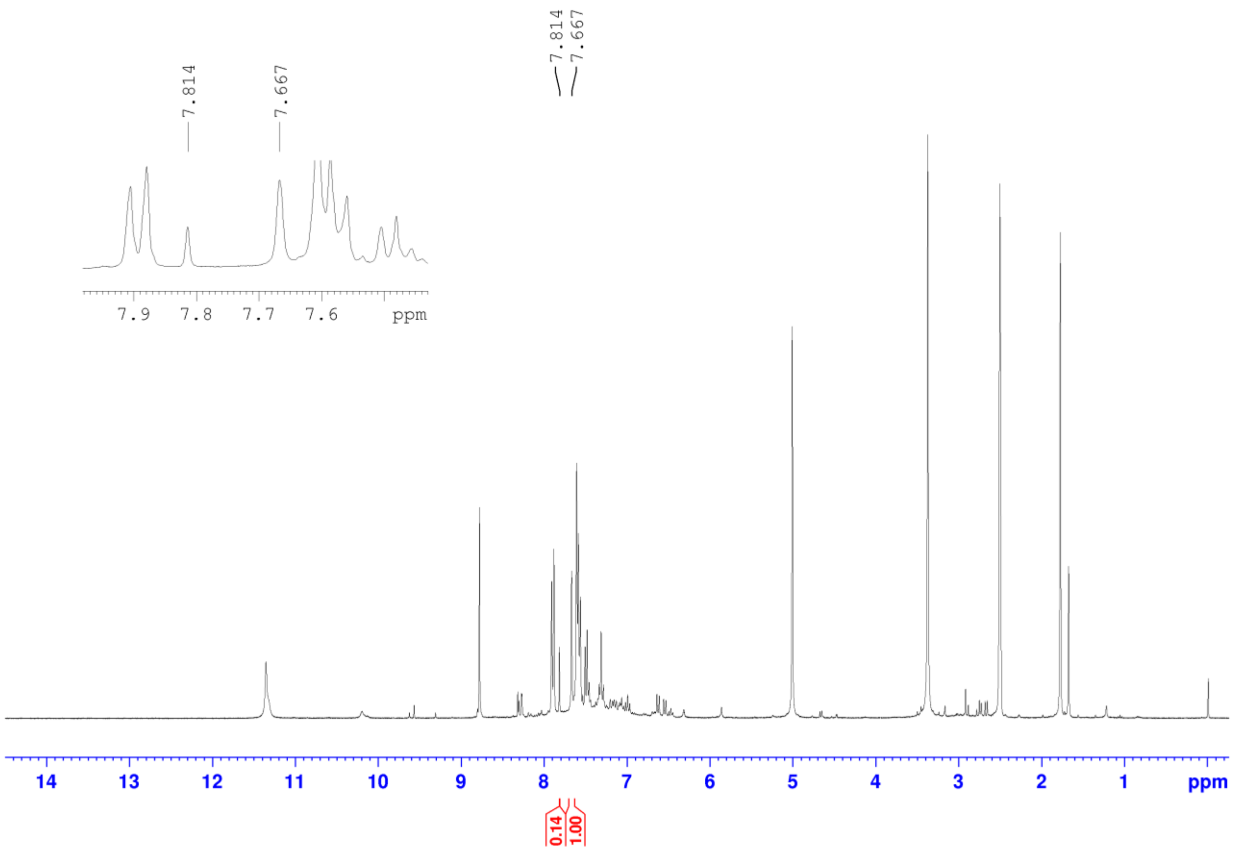

1-[(1-phenyl-1,2,3-triazol-4-yl)-methyl]-thymine (14b).

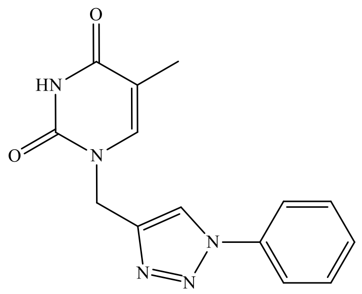

^{1}H-NMR

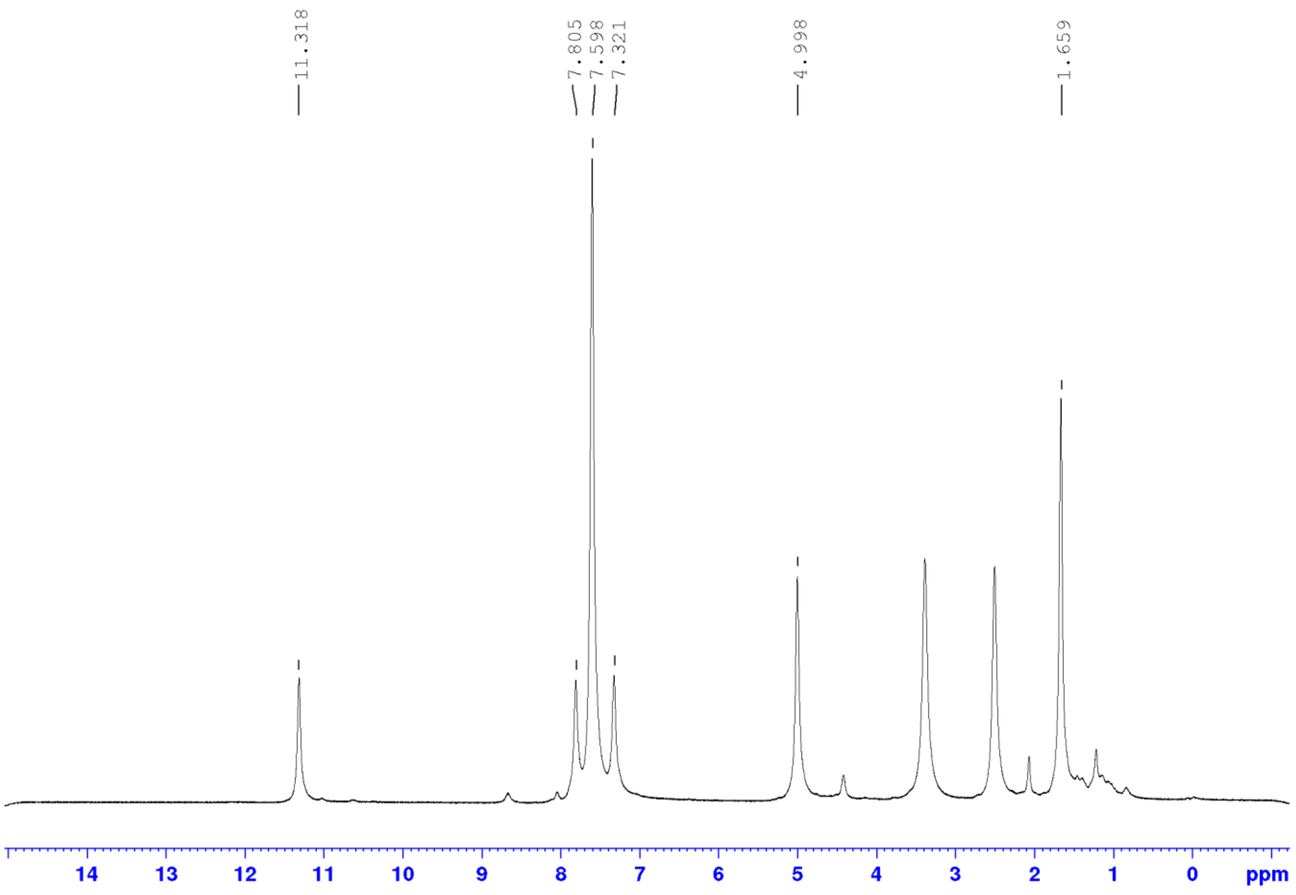

^{13}C-APT NMR

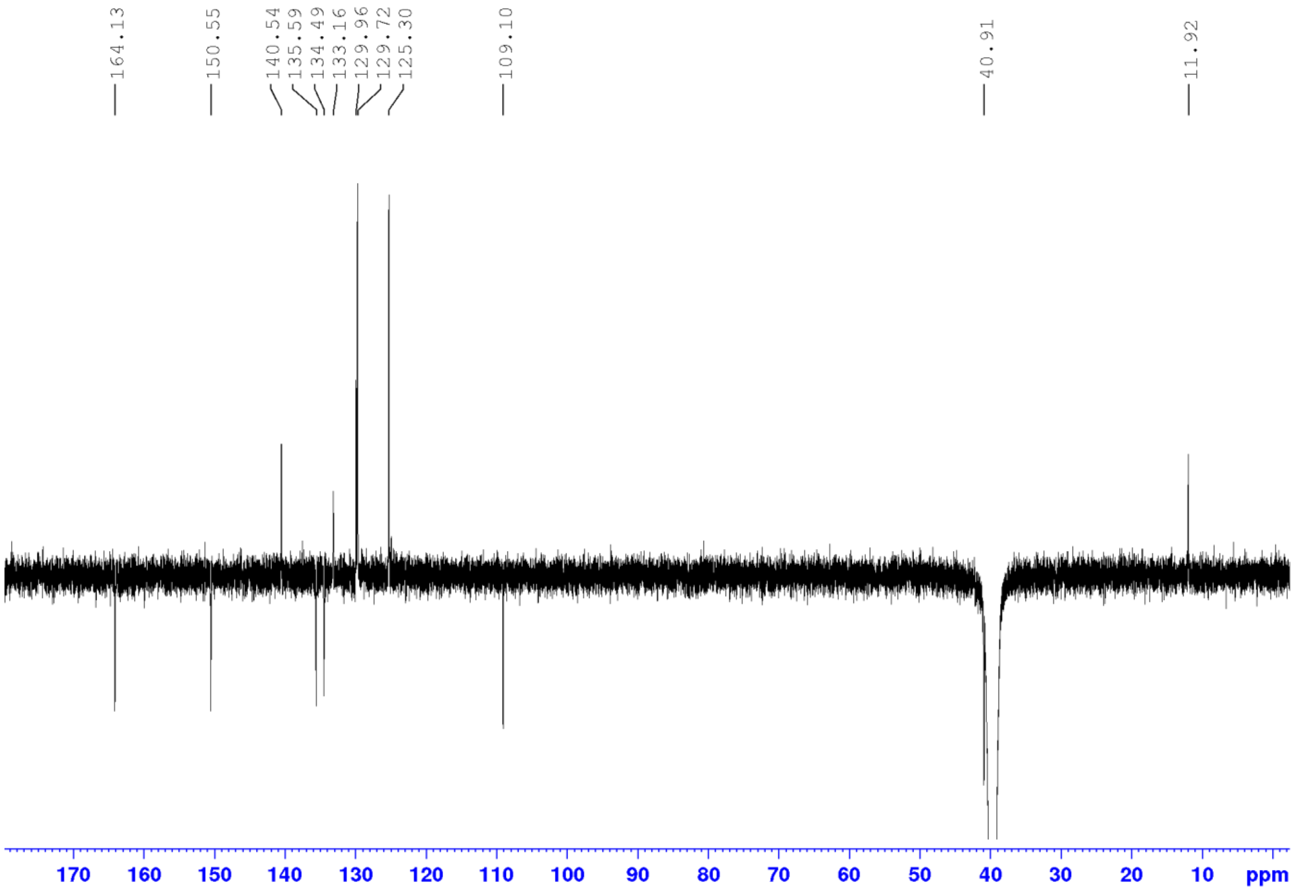
164.13
150.55
140.54
135.59
134.49
133.16
129.96
129.72
125.30
109.10
40.91
11.92
170
160
150
140
130
120
110
100
90
80
70
60
50
40
30
20
10
ppm

1-[1-phenyl-1,2,3-triazol-5-yl-methyl]-uracil (15a)

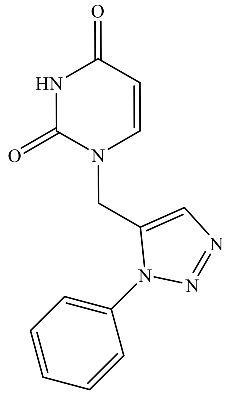

^{1}H NMR

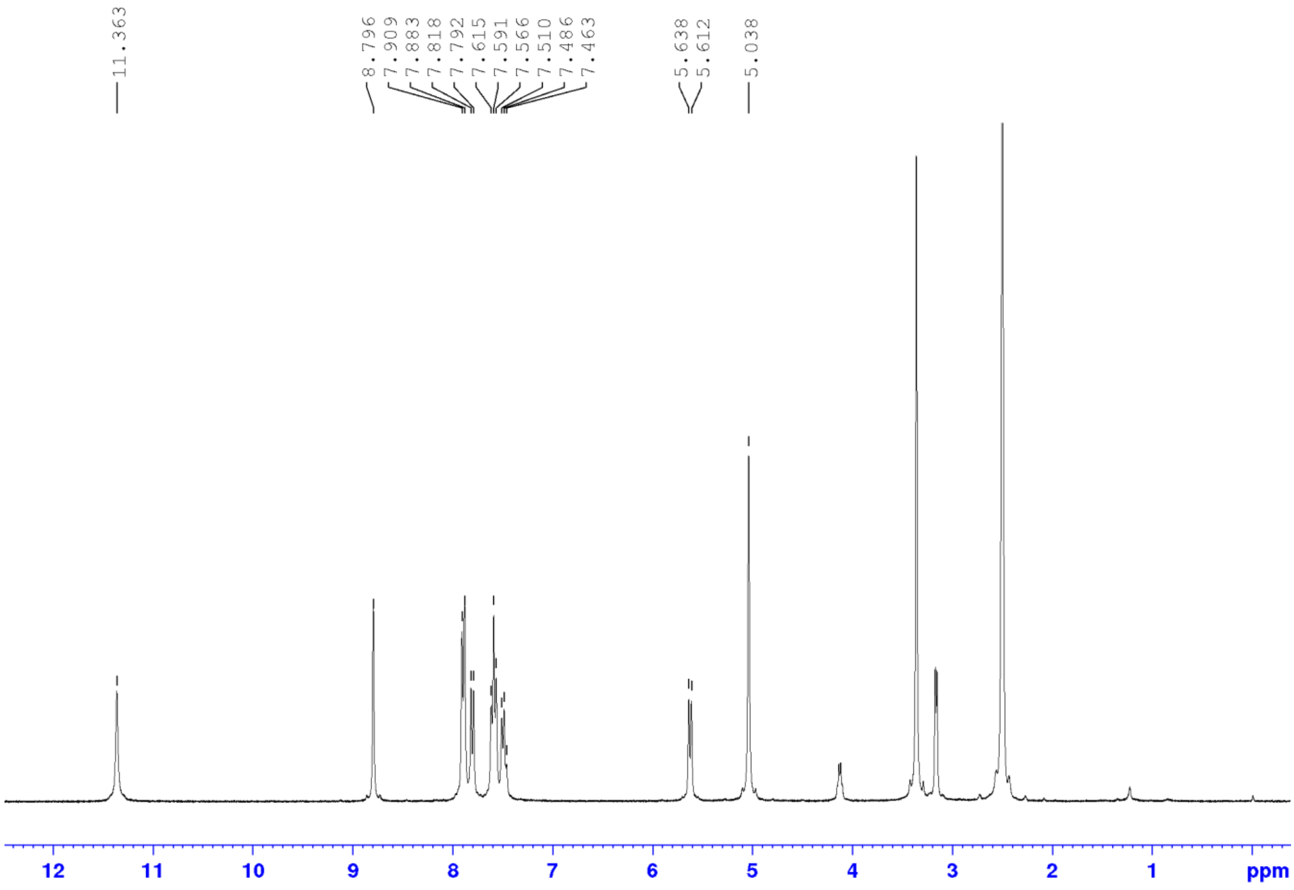

^{13}C-APT NMR

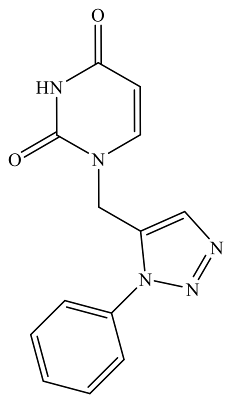
O
HN
O
N
N
N
N

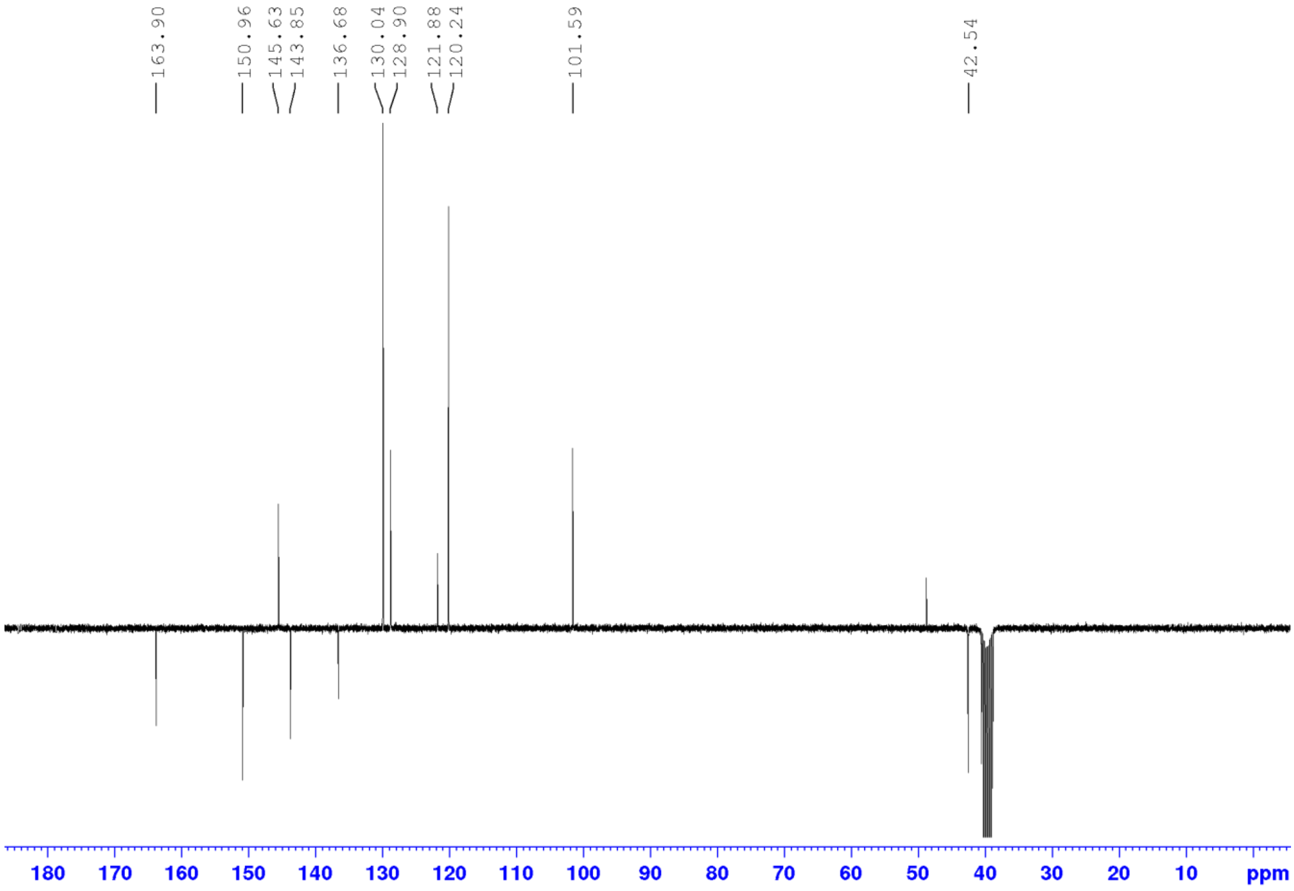
163.90
150.96
145.63
143.85
136.68
130.04
128.90
121.88
120.24
101.59
42.54
180
170
160
150
140
130
120
110
100
90
80
70
60
50
40
30
20
10
ppm

COSY NMR

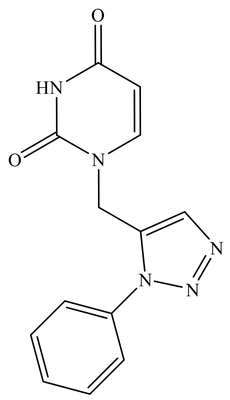

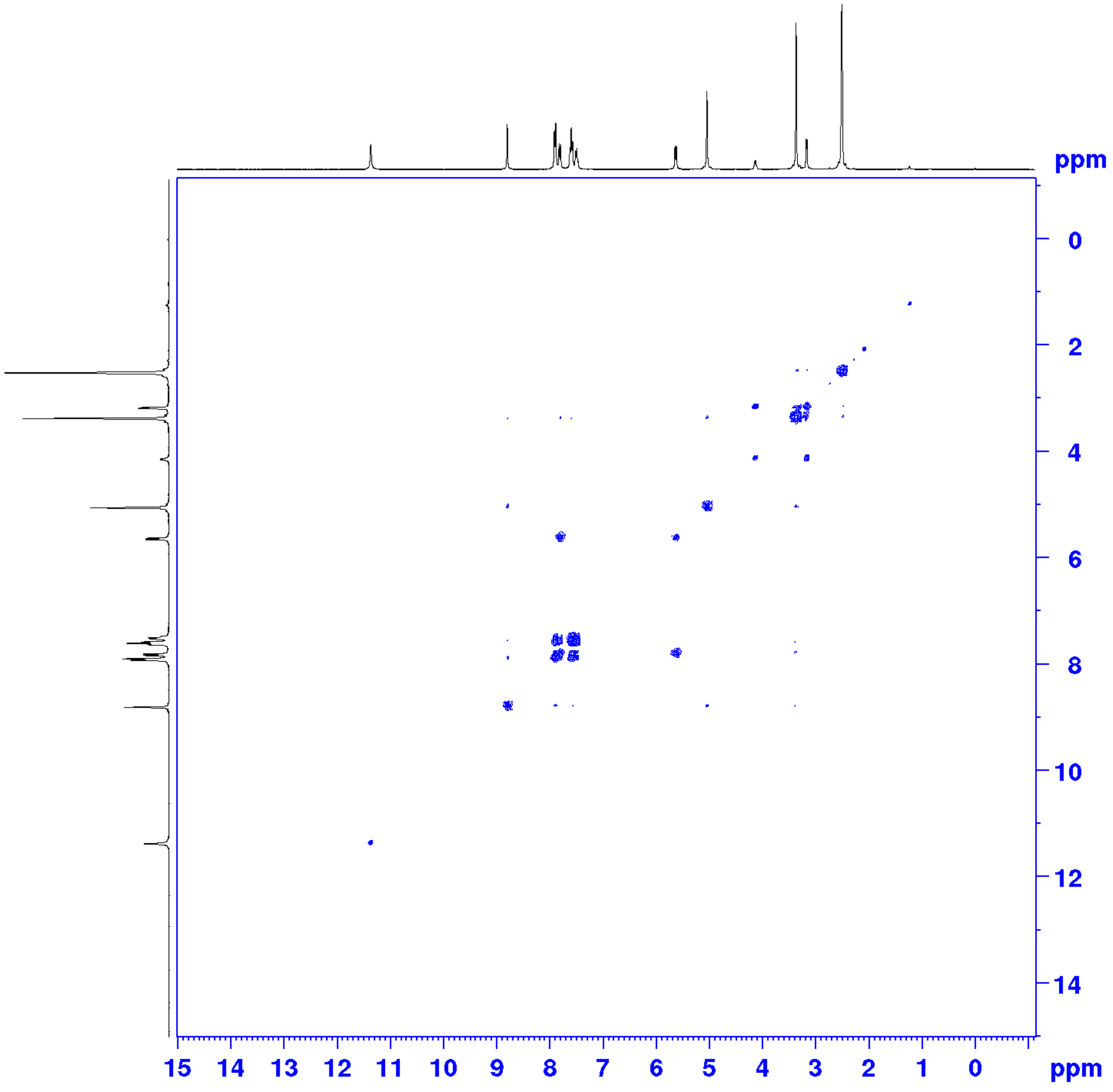

HRMS

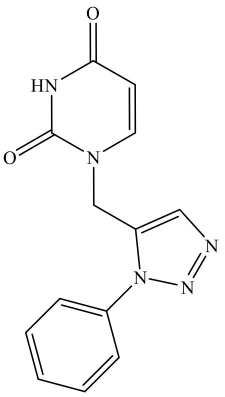
O
HN
O
N
N
N
N

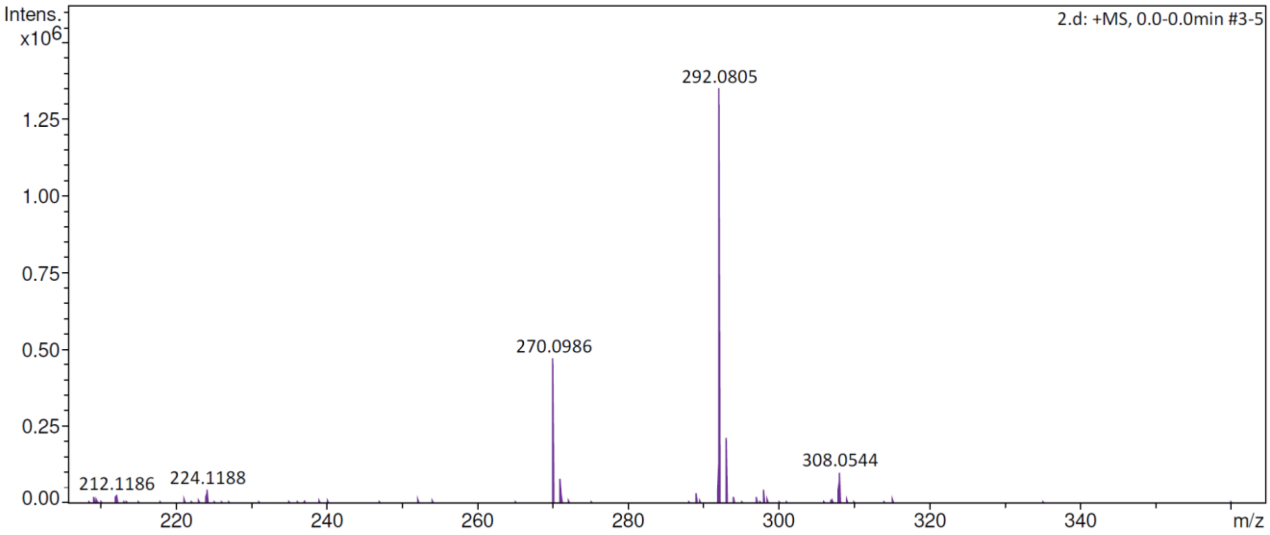
Intens.
x10^6
2.d: +MS, 0.0-0.0min #3-5
292.0805
270.0986
308.0544
212.1186
224.1188
1.25
1.00
0.75
0.50
0.25
0.00
220
240
260
280
300
320
340
m/z

5-fluoro-1-[1-phenyl-1,2,3-triazol-5-yl-methyl]-uracil (16a).

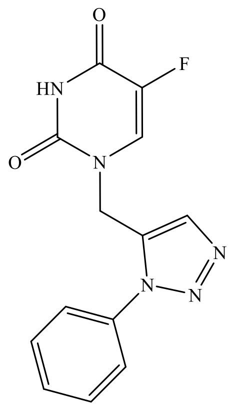

^{1}H NMR

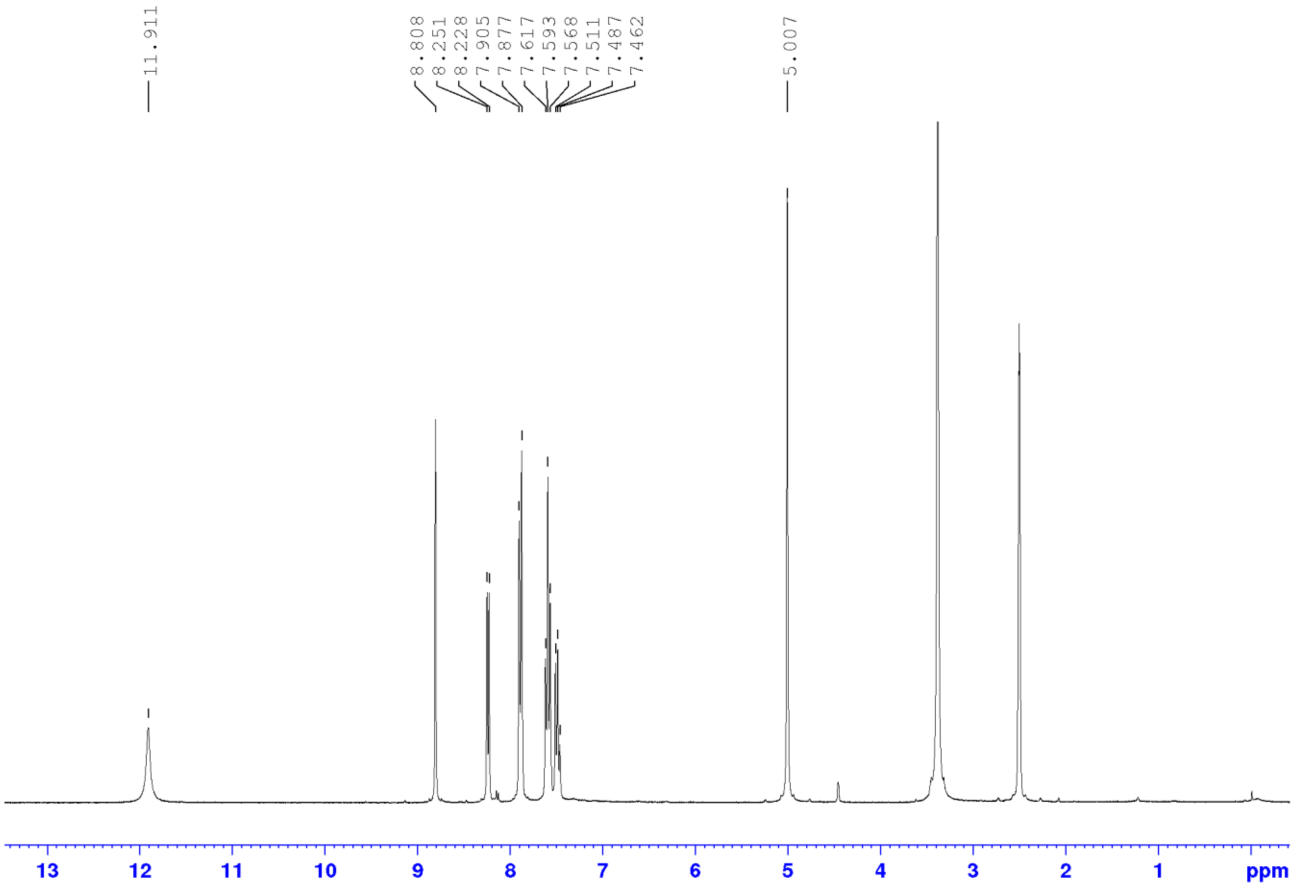

^{13}C-APT NMR

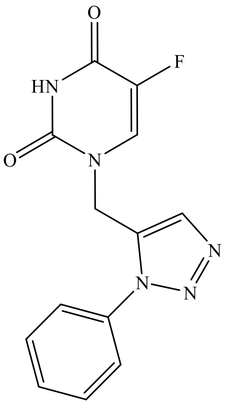

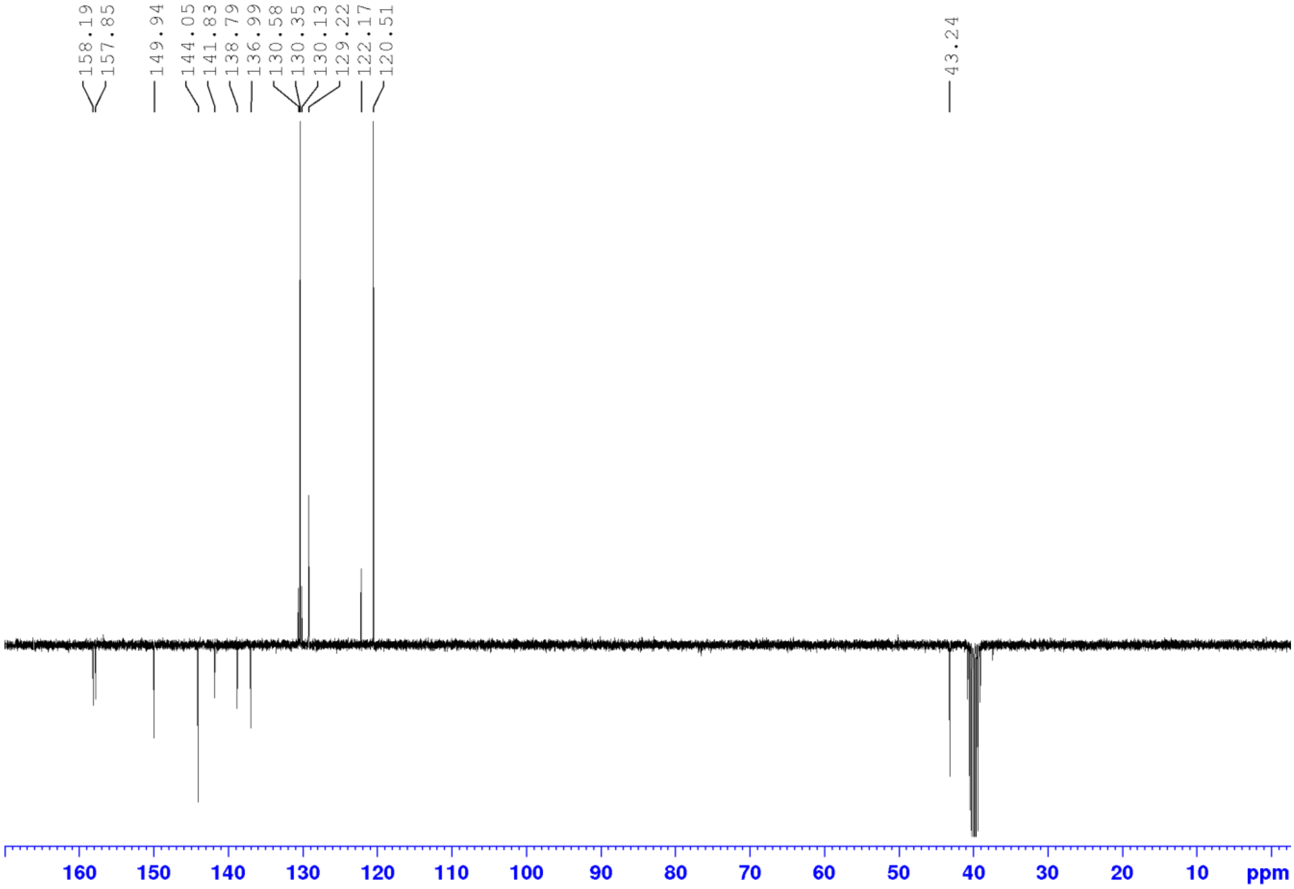
158.19
157.85
149.94
144.05
141.83
138.79
136.99
130.58
130.35
130.13
129.22
122.17
120.51
43.24
160
150
140
130
120
110
100
90
80
70
60
50
40
30
20
10
ppm

COSY NMR

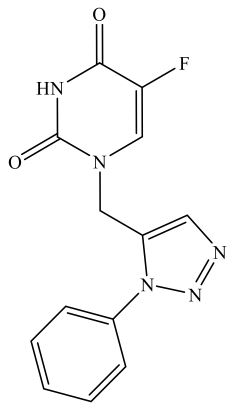

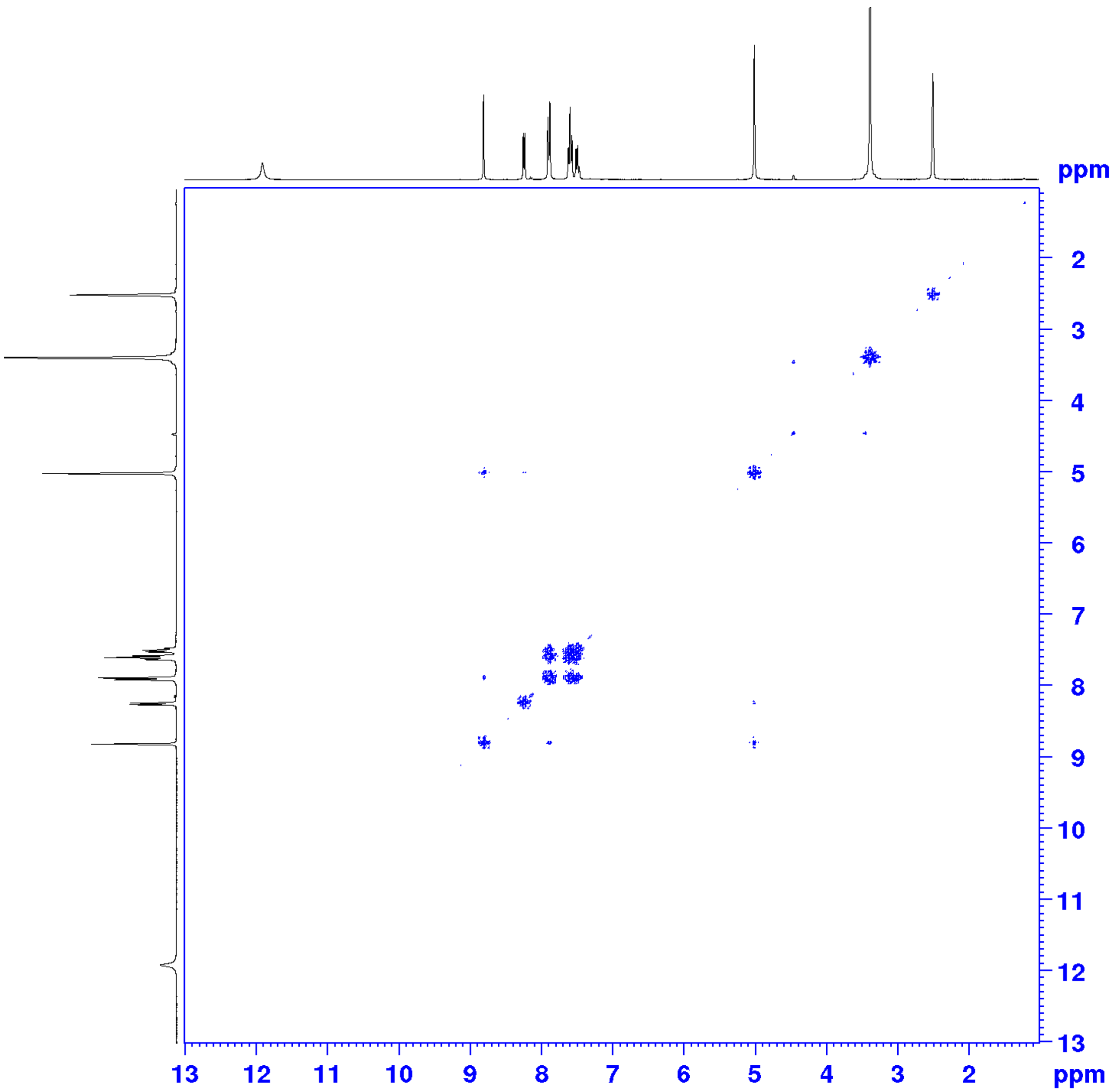

HRMS

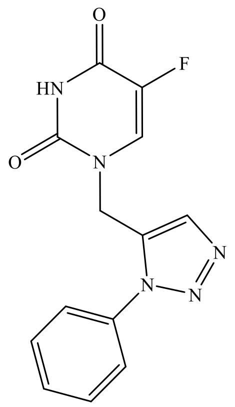
O
F
HN
O
N
N
N
N

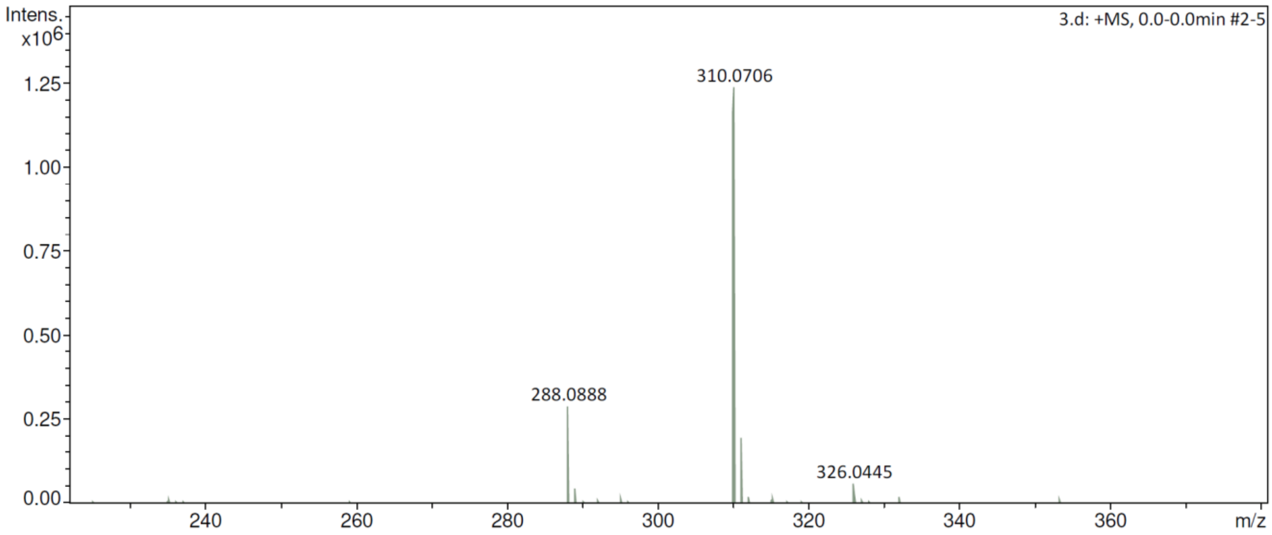
Intens.
x10^6
3.d: +MS, 0.0-0.0min #2-5
310.0706
288.0888
326.0445
1.25
1.00
0.75
0.50
0.25
0.00
240
260
280
300
320
340
360
m/z

1-[1-benzyl-1,2,3-triazol-5-yl-methyl]-thymine (17a)

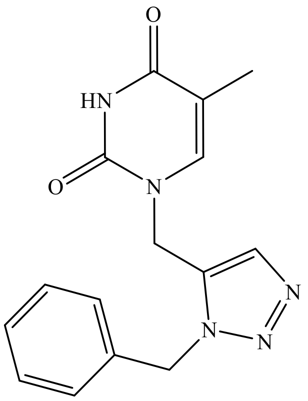

¹H NMR

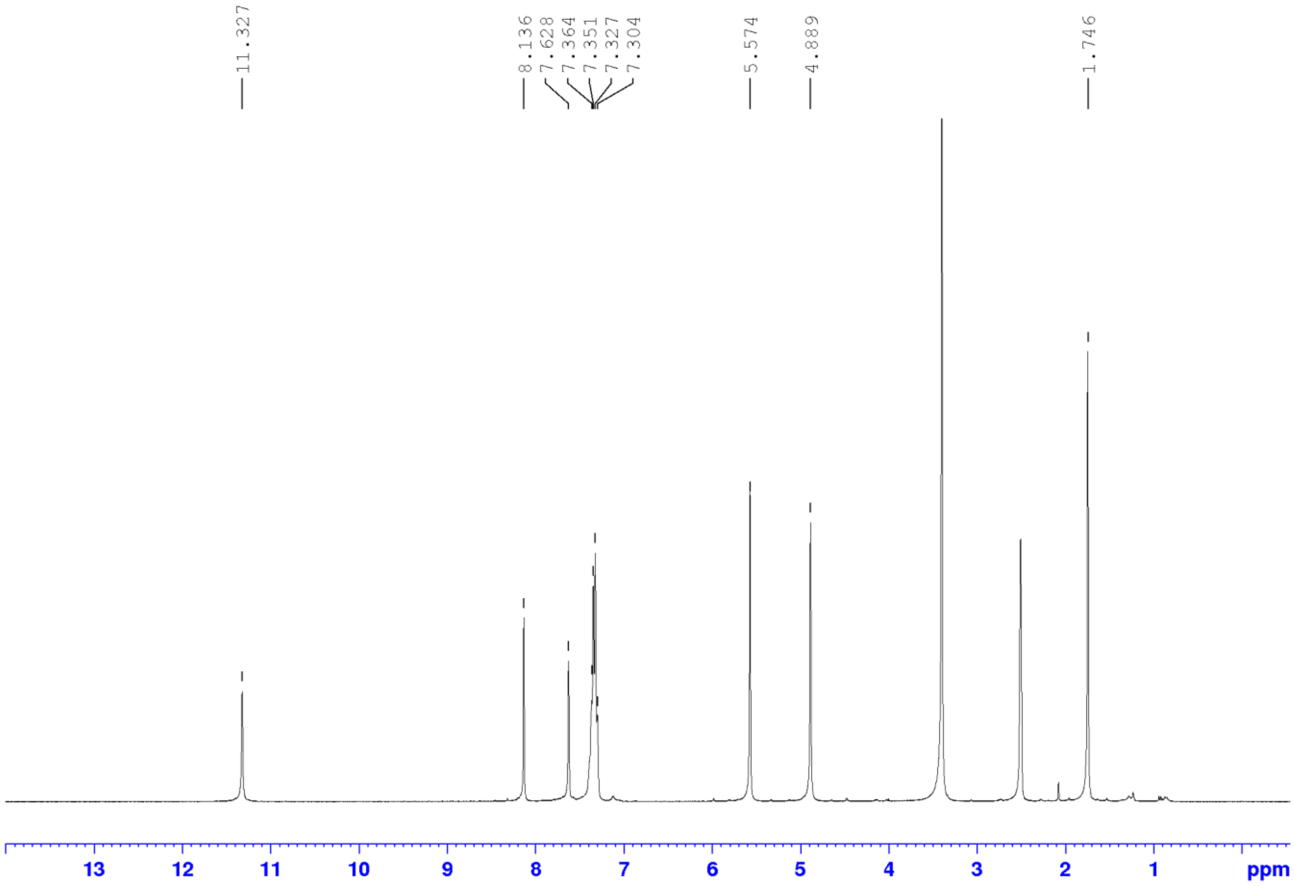

^{13}C-APT NMR

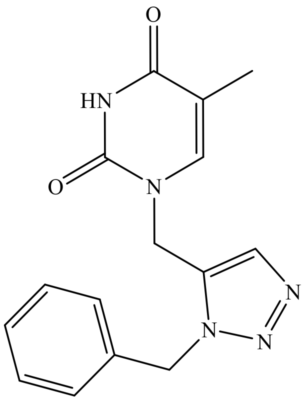

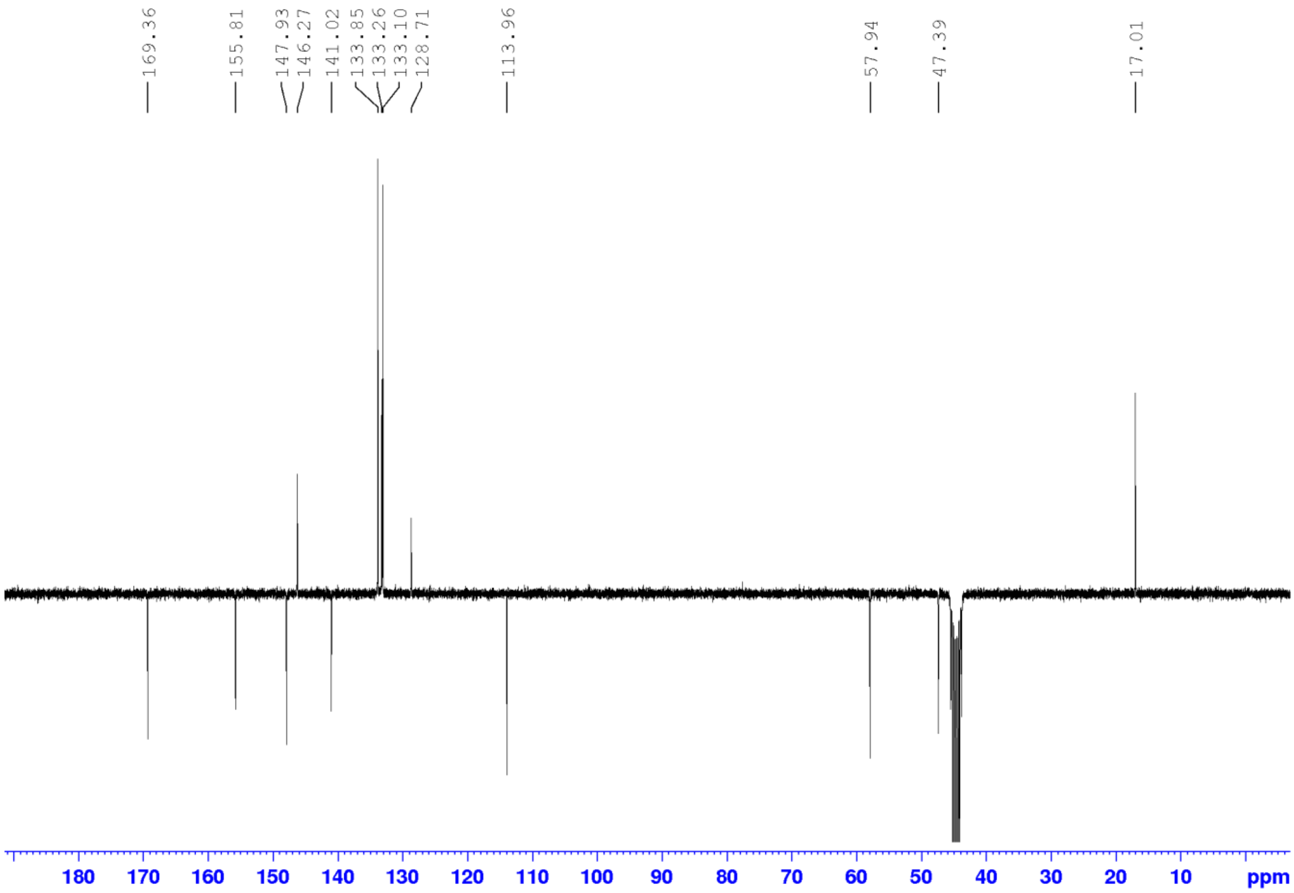
169.36
155.81
147.93
146.27
141.02
133.85
133.26
133.10
128.71
113.96
57.94
47.39
17.01
180 170 160 150 140 130 120 110 100 90 80 70 60 50 40 30 20 10 ppm

COSY NMR

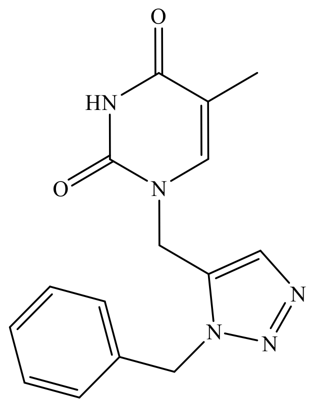

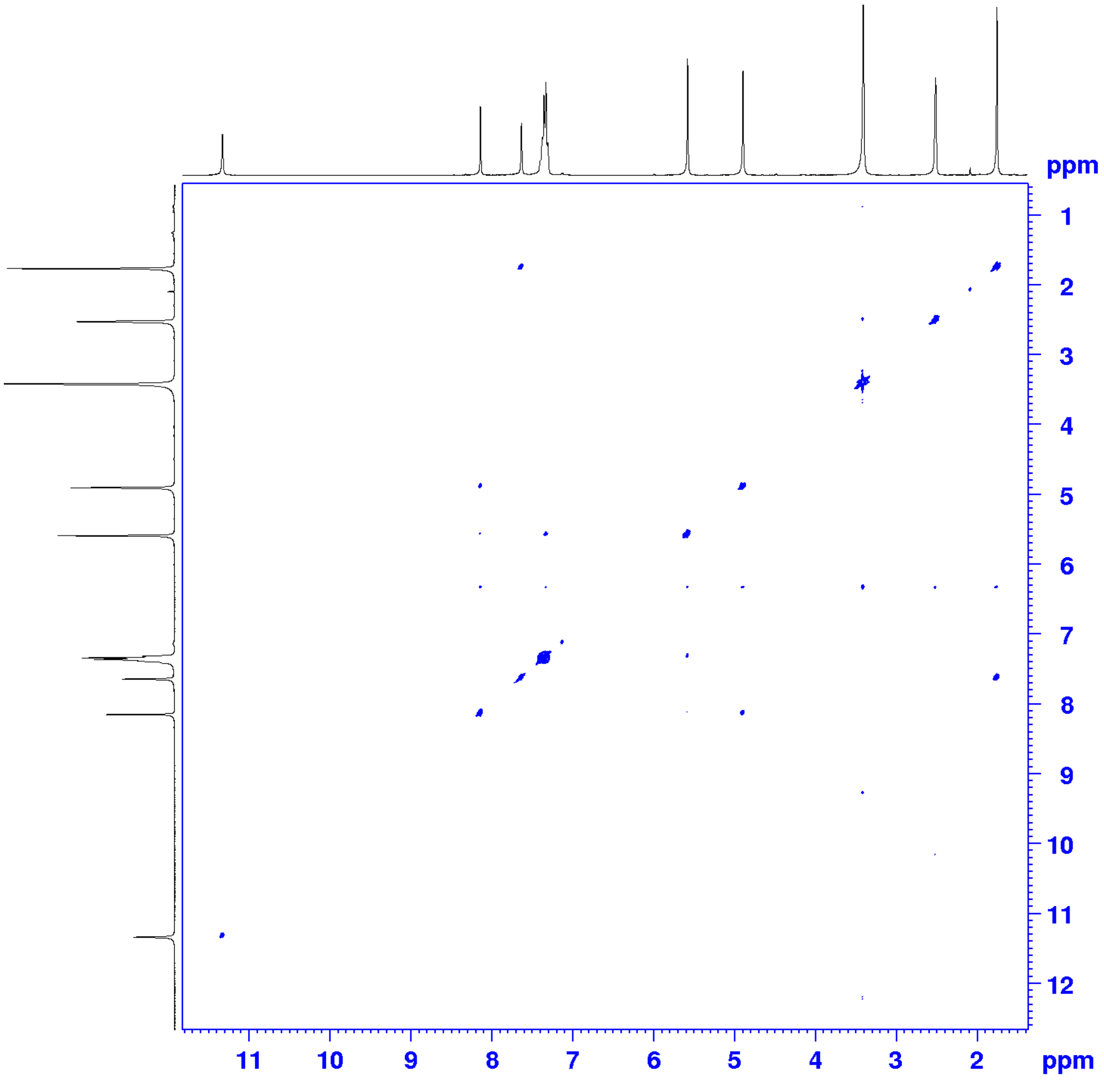

HRMS

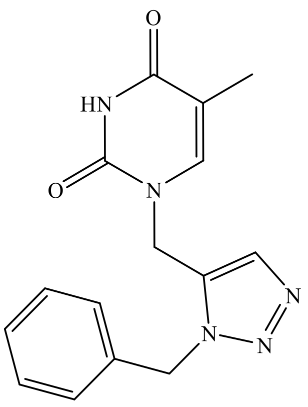

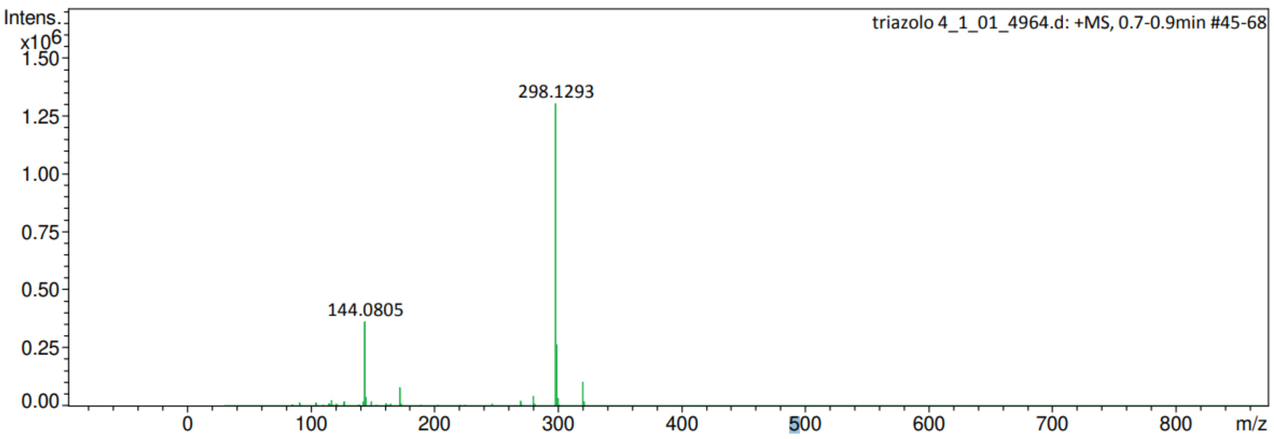
triazolo 4_1_01_4964.d: +MS, 0.7-0.9min #45-68
Intens.
x10^6
1.50
1.25
1.00
0.75
0.50
0.25
0.00
298.1293
144.0805
0
100
200
300
400
500
600
700
800
m/z

1-[1-benzyl-1,2,3-triazol-5-yl-methyl]-uracil (18a).

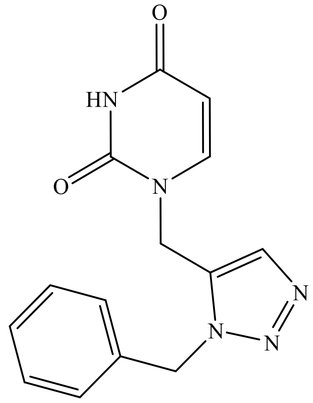

^{1}H-NMR

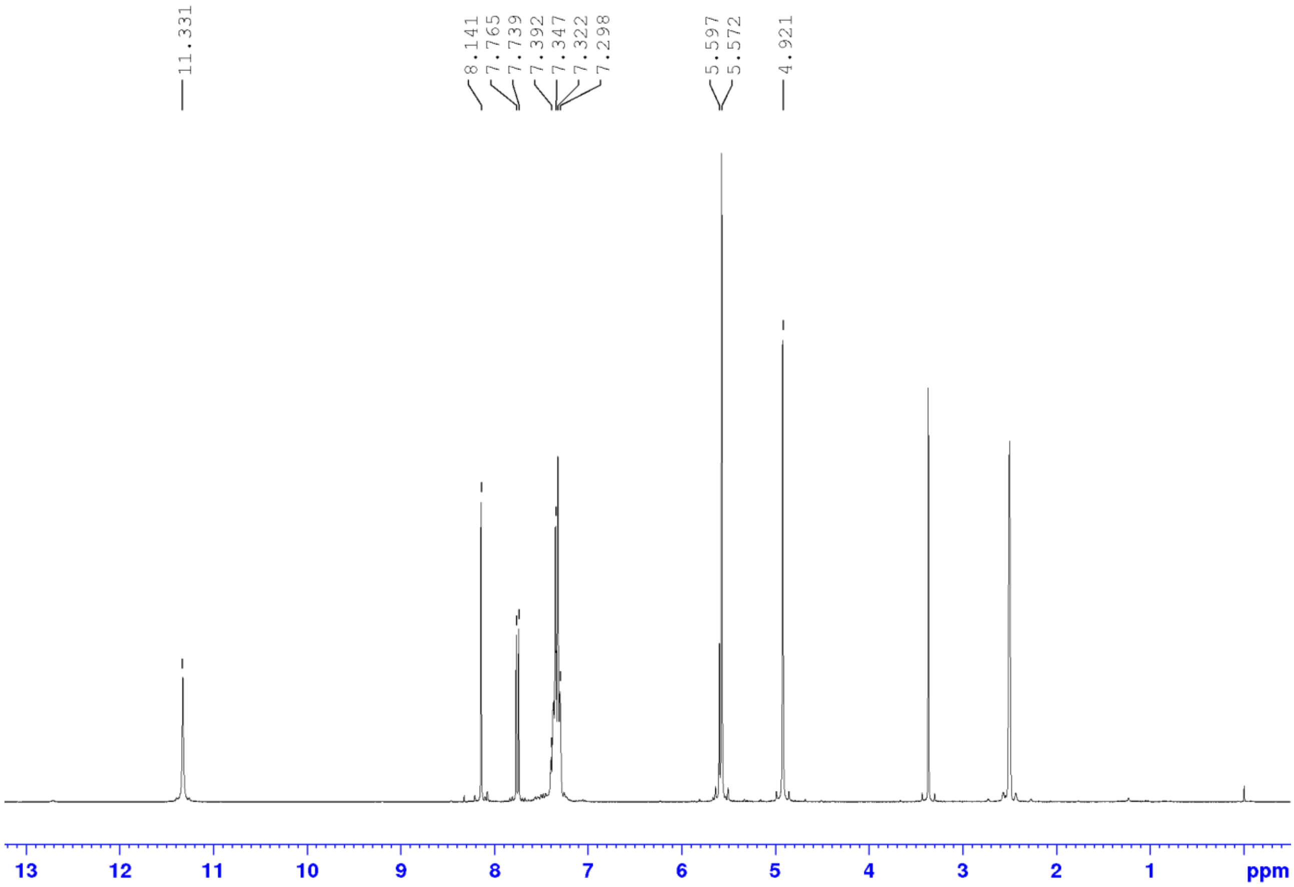

^{13}C-APT NMR

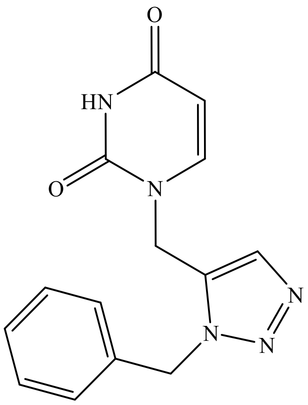

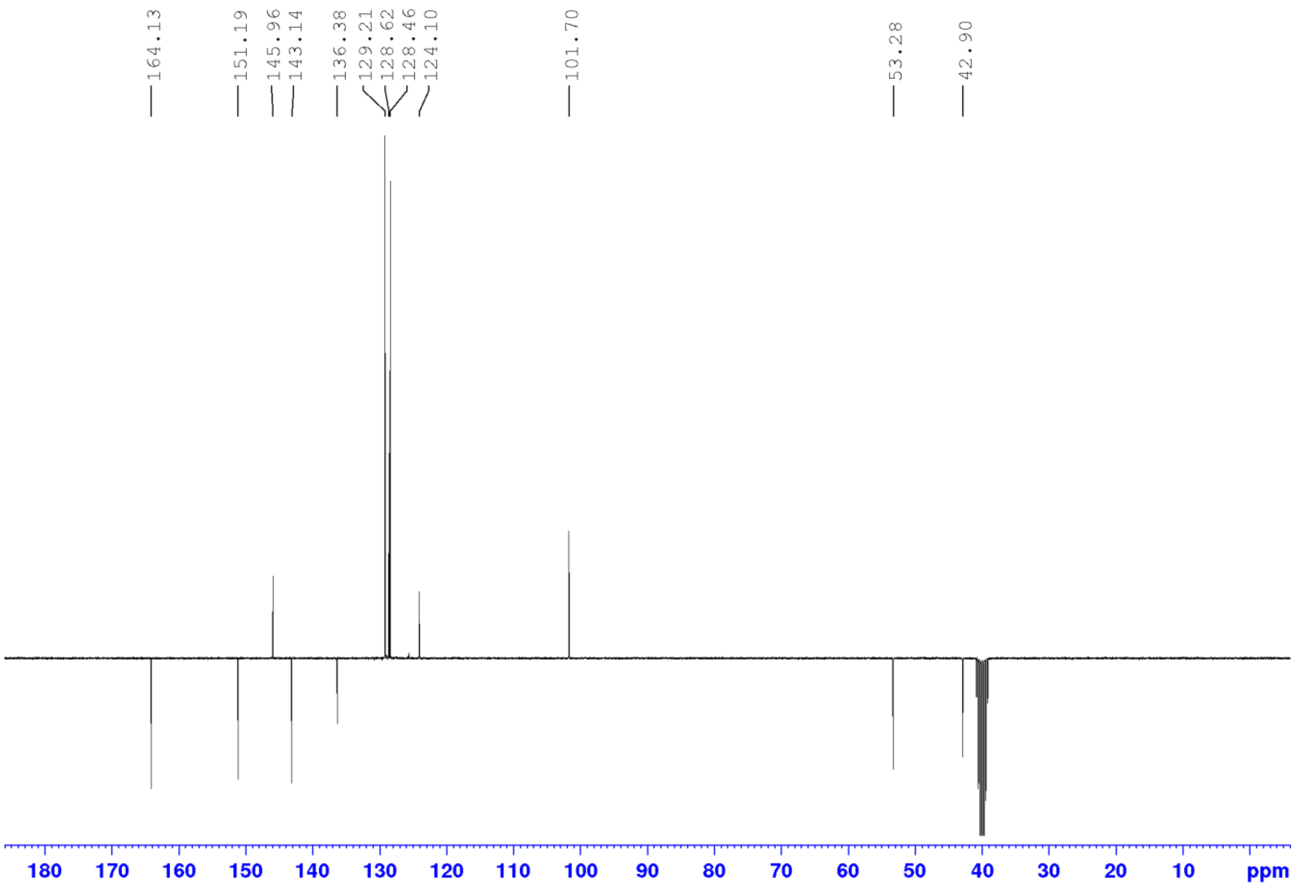
164.13
151.19
145.96
143.14
136.38
129.21
128.62
128.46
124.10
101.70
53.28
42.90
180
170
160
150
140
130
120
110
100
90
80
70
60
50
40
30
20
10
ppm

COSY NMR

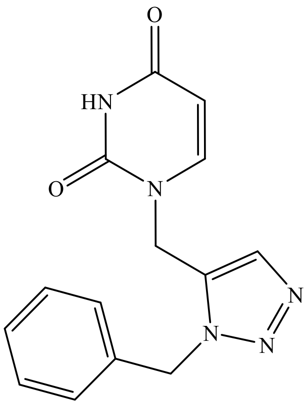

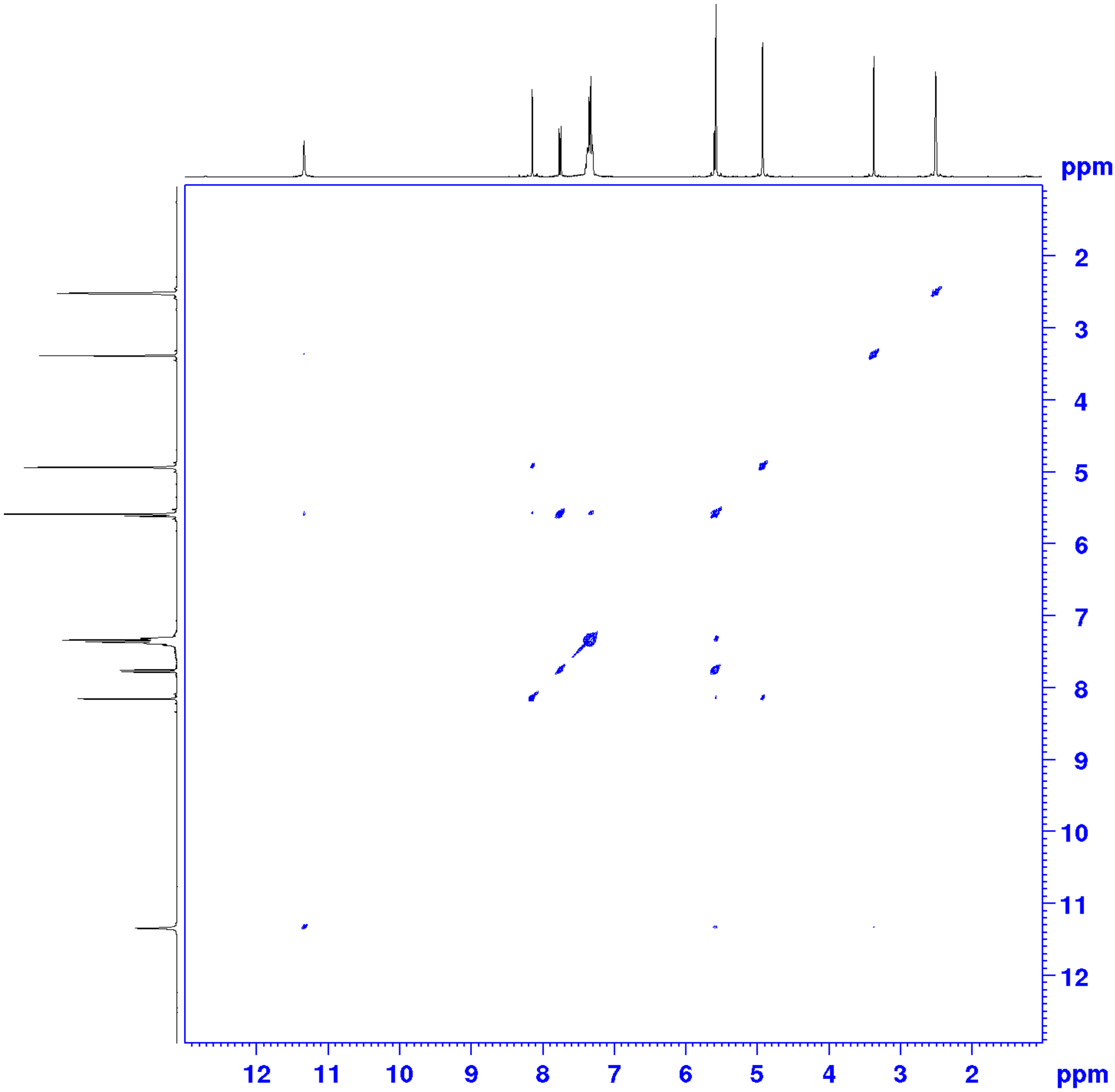

HRMS

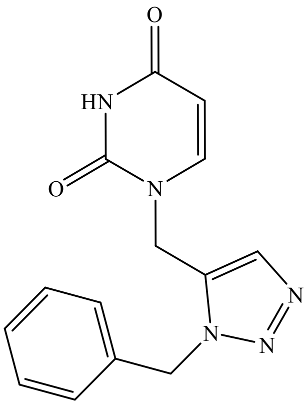
O
HN
O
N
N
N
N

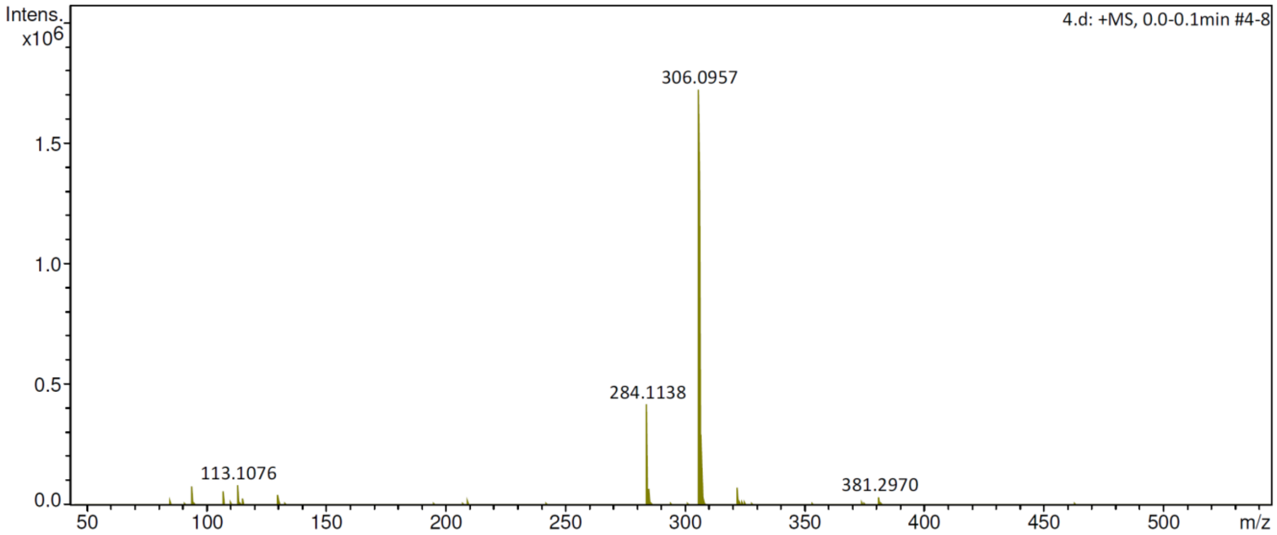
Intens.
x10^6
4.d: +MS, 0.0-0.1min #4-8
306.0957
284.1138
113.1076
381.2970
1.5
1.0
0.5
0.0
50
100
150
200
250
300
350
400
450
500
m/z

5-fluoro-1-[1-benzyl-1,2,3-triazol-5-yl-methyl]-uracil (19a).

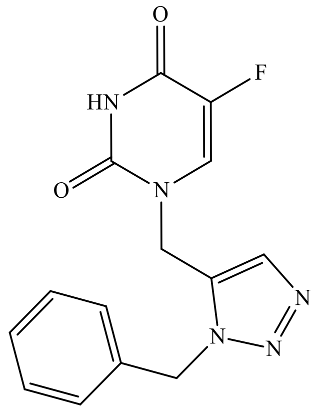

^{1}H NMR

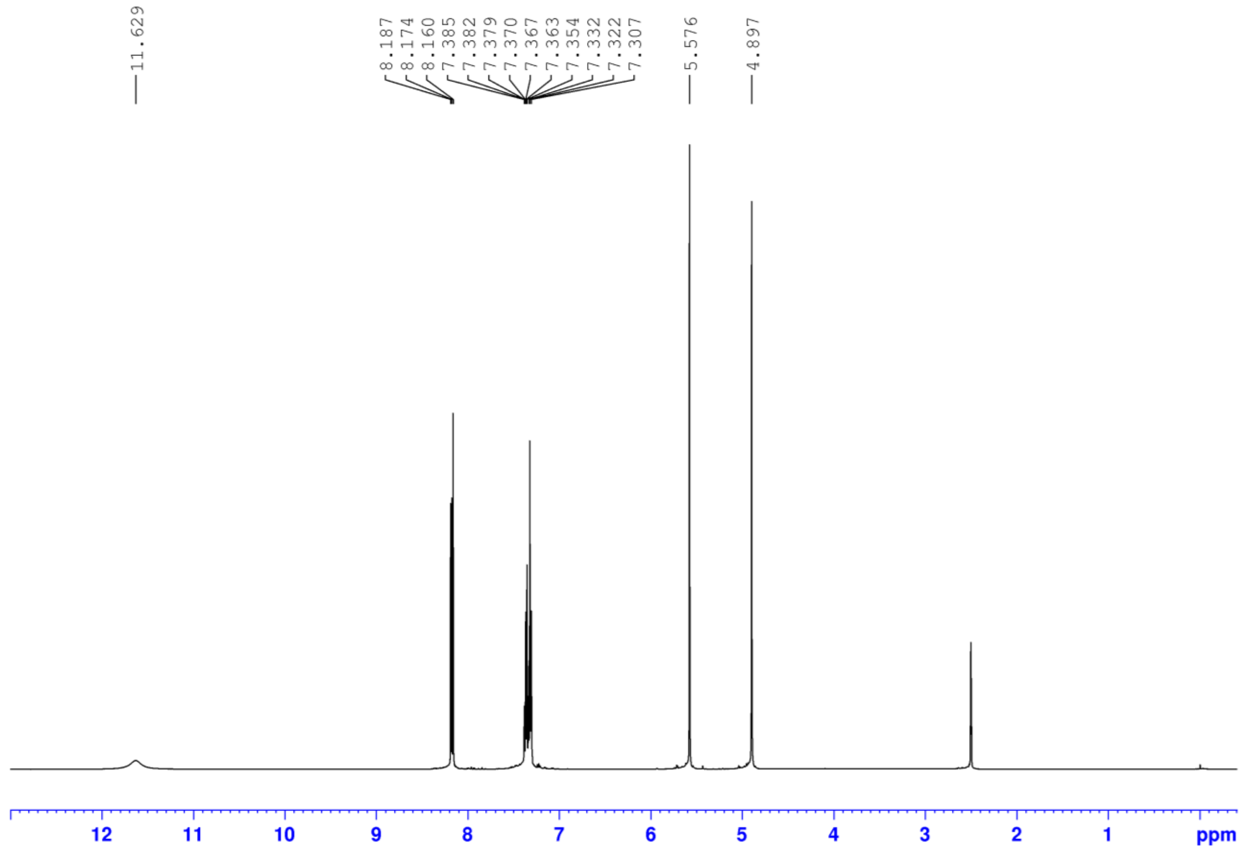

^{13}C NMR

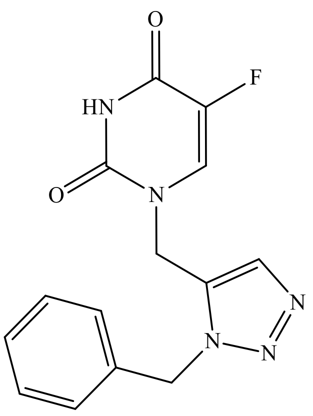
O
F
HN
O
N
N
N
N

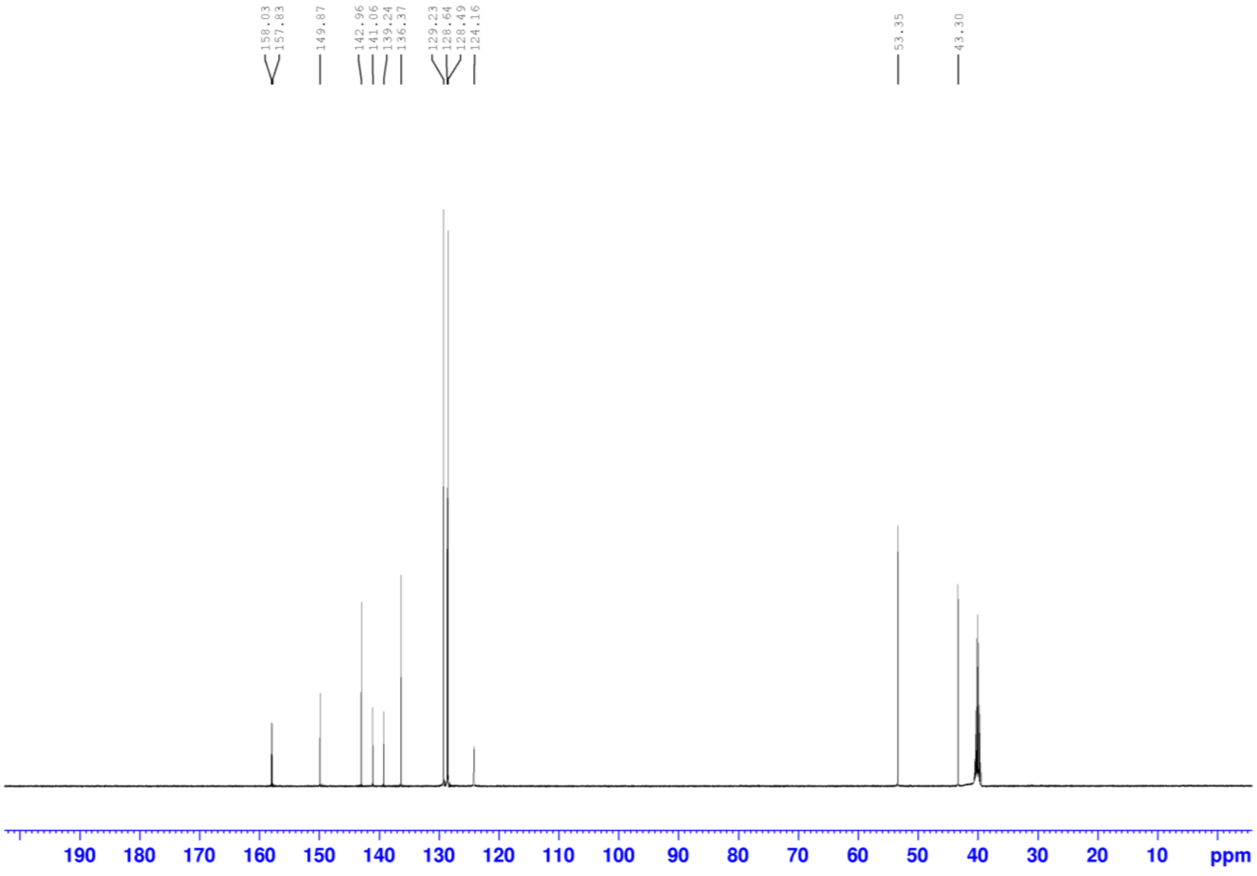
158.03
157.83
149.87
142.96
141.06
139.24
136.37
129.23
128.64
128.49
124.16
53.35
43.30
190
180
170
160
150
140
130
120
110
100
90
80
70
60
50
40
30
20
10
ppm

COSY NMR

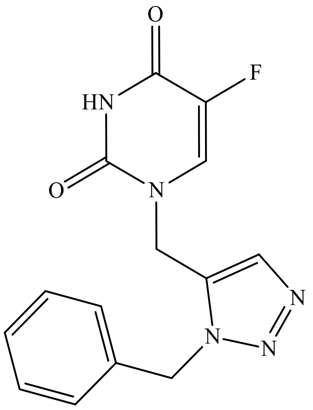

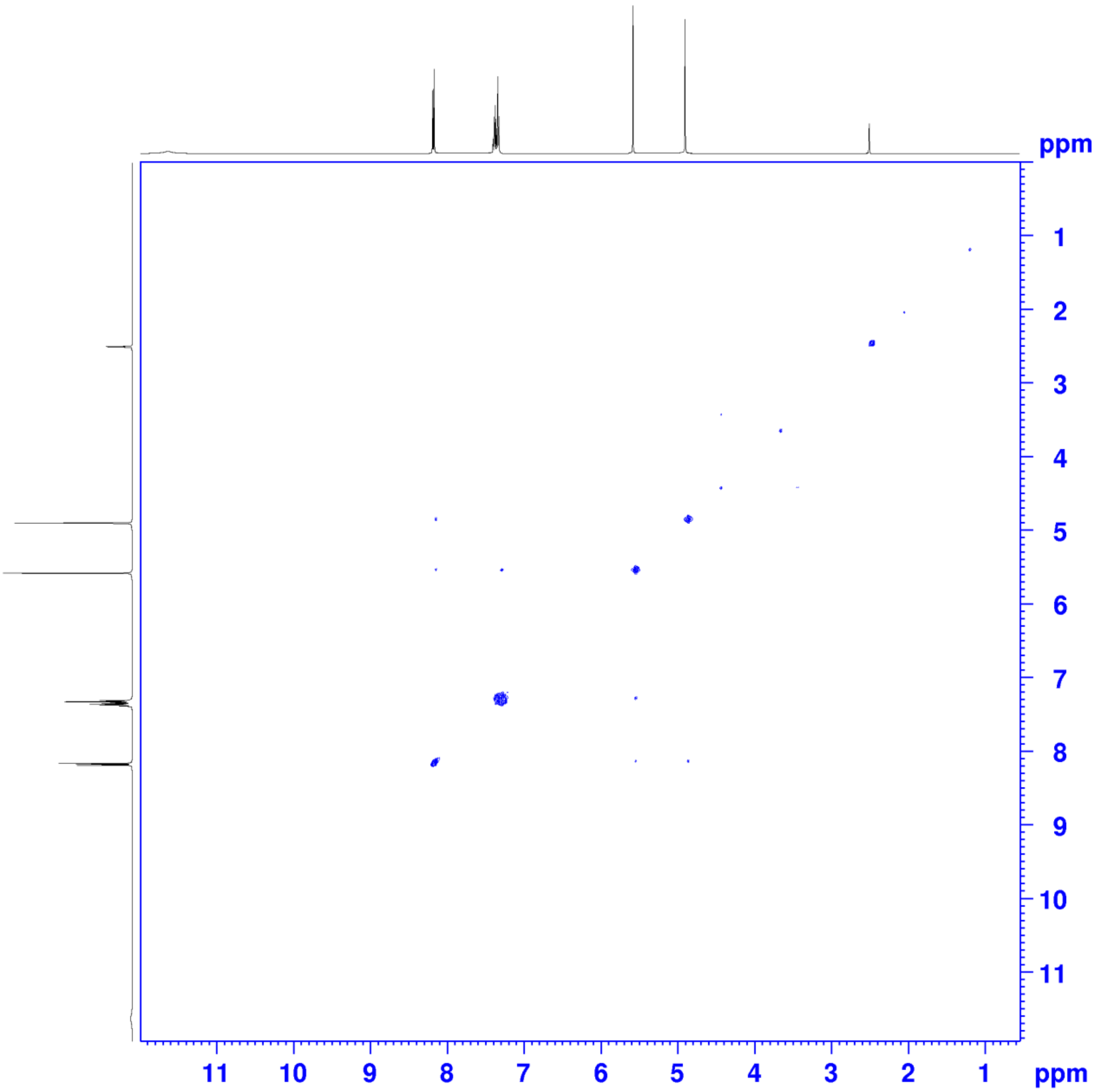

HRMS

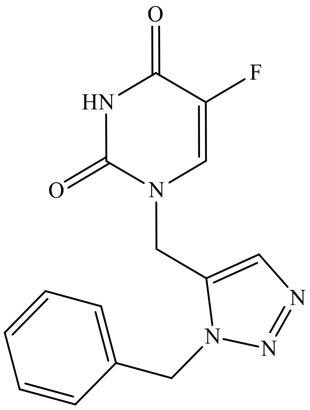

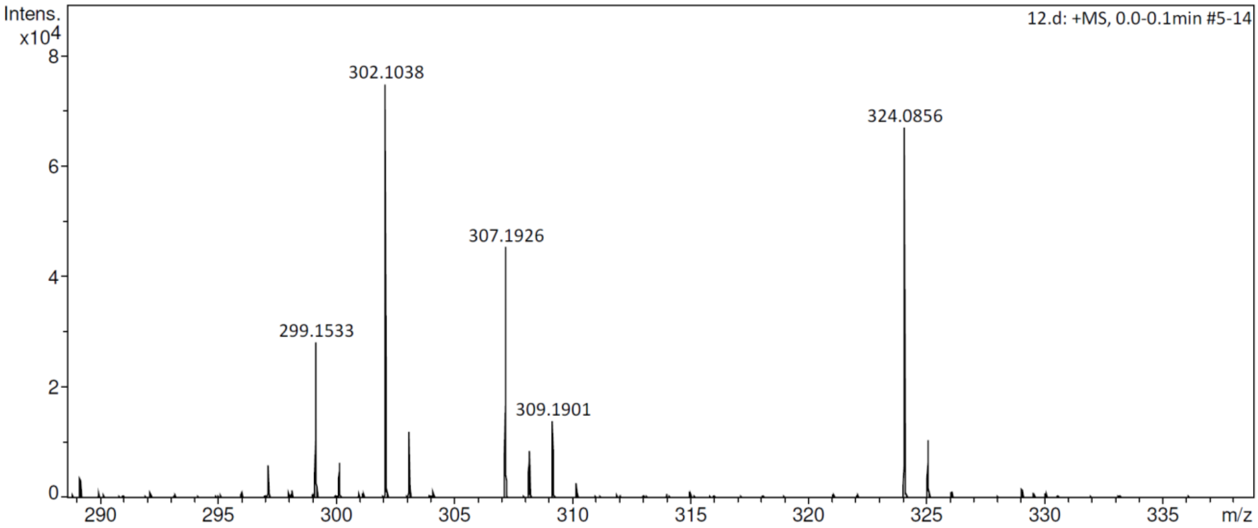
Intens.
x10^4
8
6
4
2
0
12.d: +MS, 0.0-0.1min #5-14
302.1038
324.0856
307.1926
299.1533
309.1901
290
295
300
305
310
315
320
325
330
335
m/z

1-[1-(4-nitrophenyl)-1,2,3-triazol-5-yl-methyl]-thymine (20a).

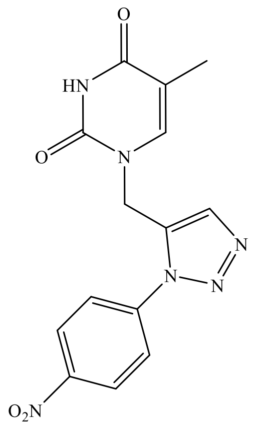

^{1}H NMR

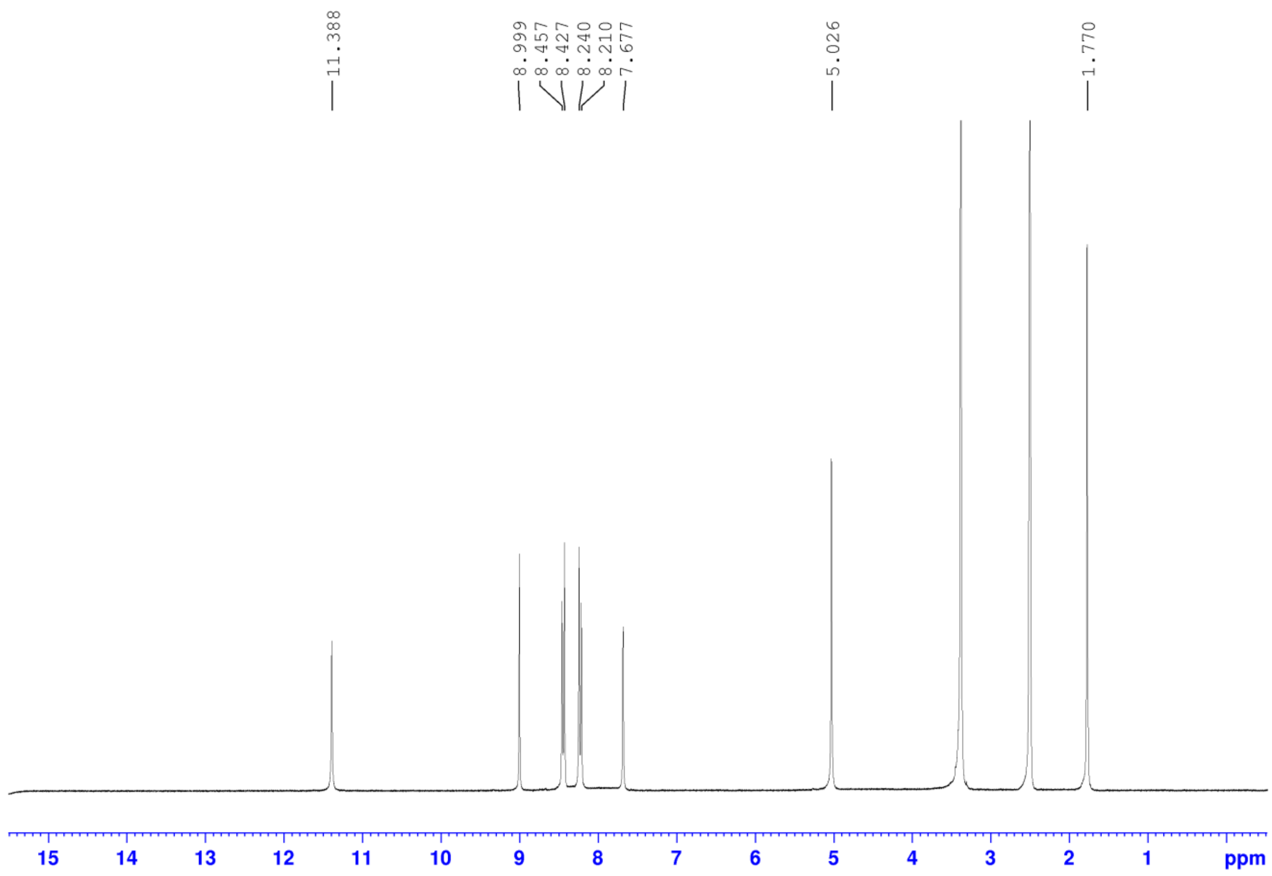

^{13}C NMR

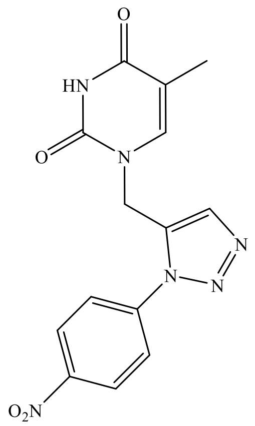
O
HN
O
N
N
N
N
O2N

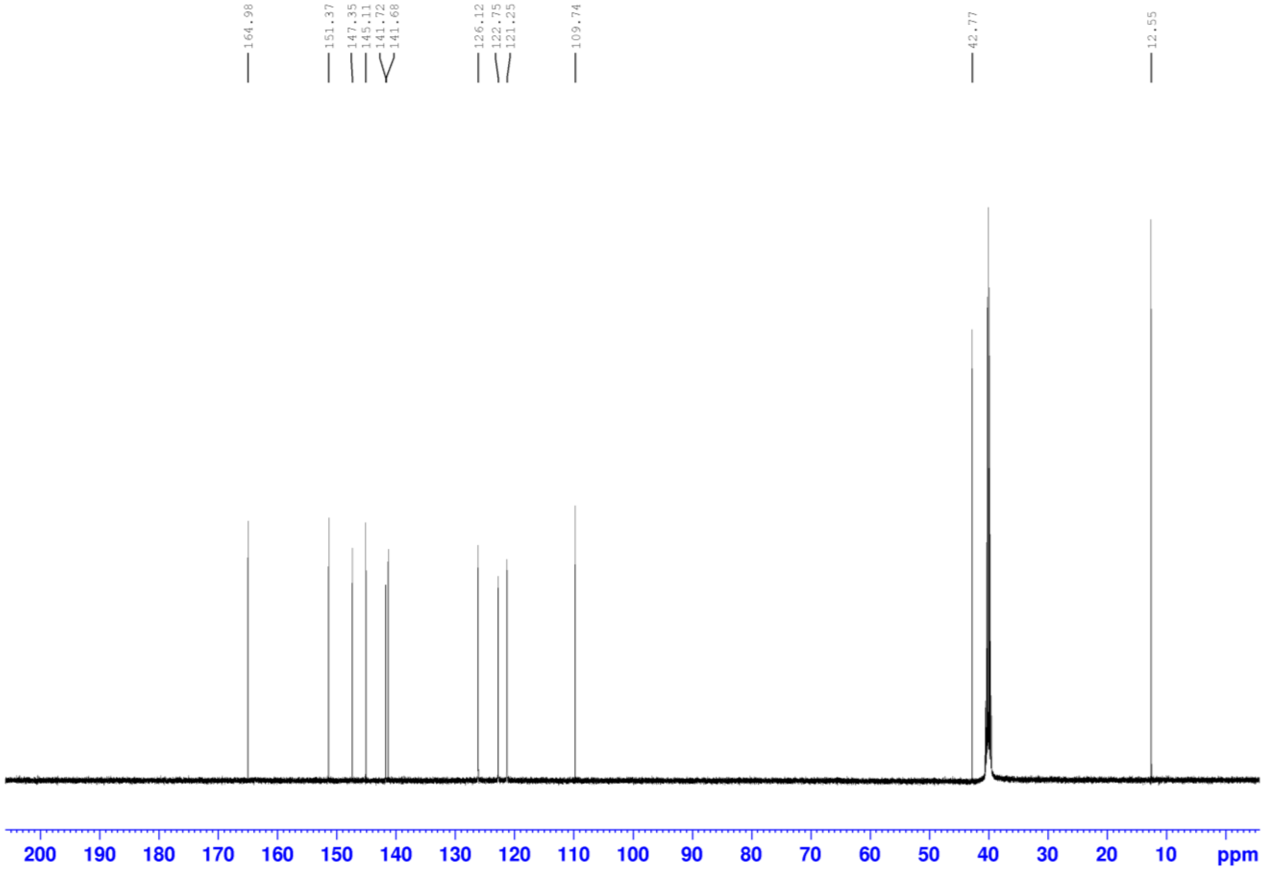
164.98
151.37
147.35
145.11
141.72
141.68
126.12
122.75
121.25
109.74
42.77
12.55
200
190
180
170
160
150
140
130
120
110
100
90
80
70
60
50
40
30
20
10
ppm

COSY NMR

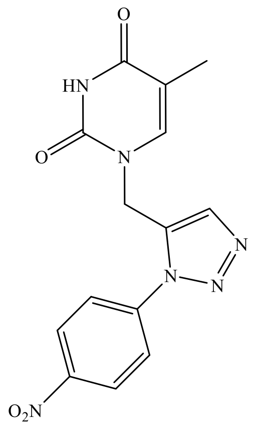

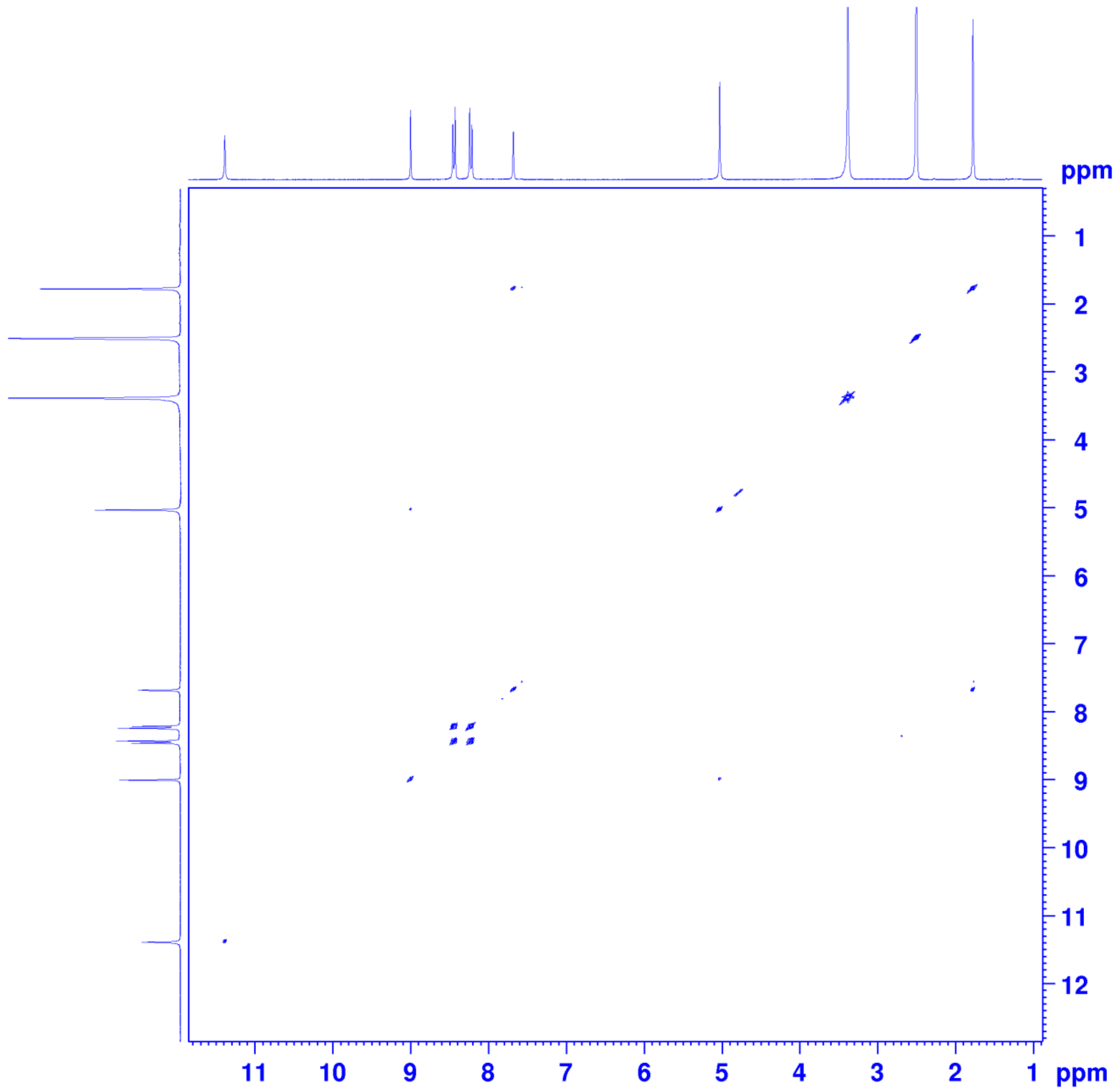

HRMS

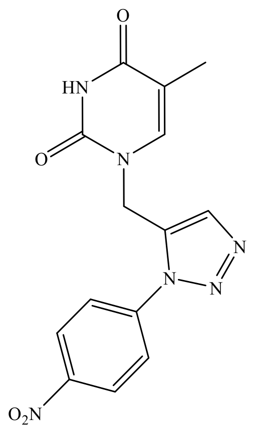
O
HN
O
N
N
N
N
O2N

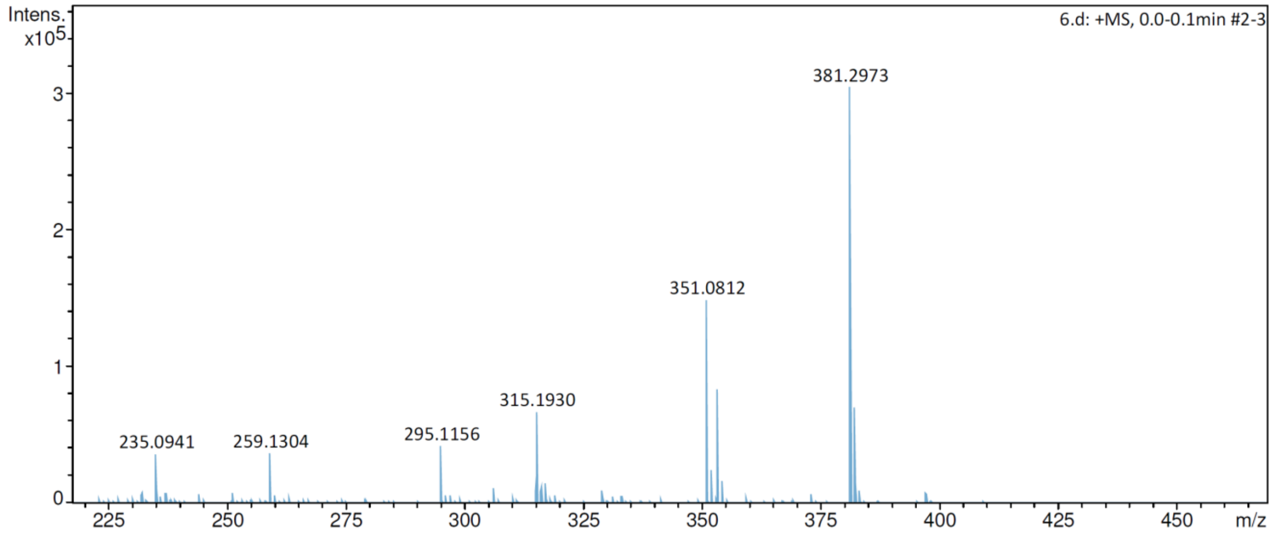
Intens.
x10^5
6.d: +MS, 0.0-0.1min #2-3
381.2973
351.0812
315.1930
295.1156
259.1304
235.0941
0
1
2
3
225
250
275
300
325
350
375
400
425
450
m/z

1-[1-(4-nitrophenyl)-1,2,3-triazol-5-yl-methyl]-uracil (21a).

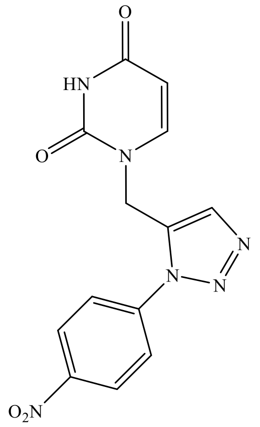

^{1}H NMR

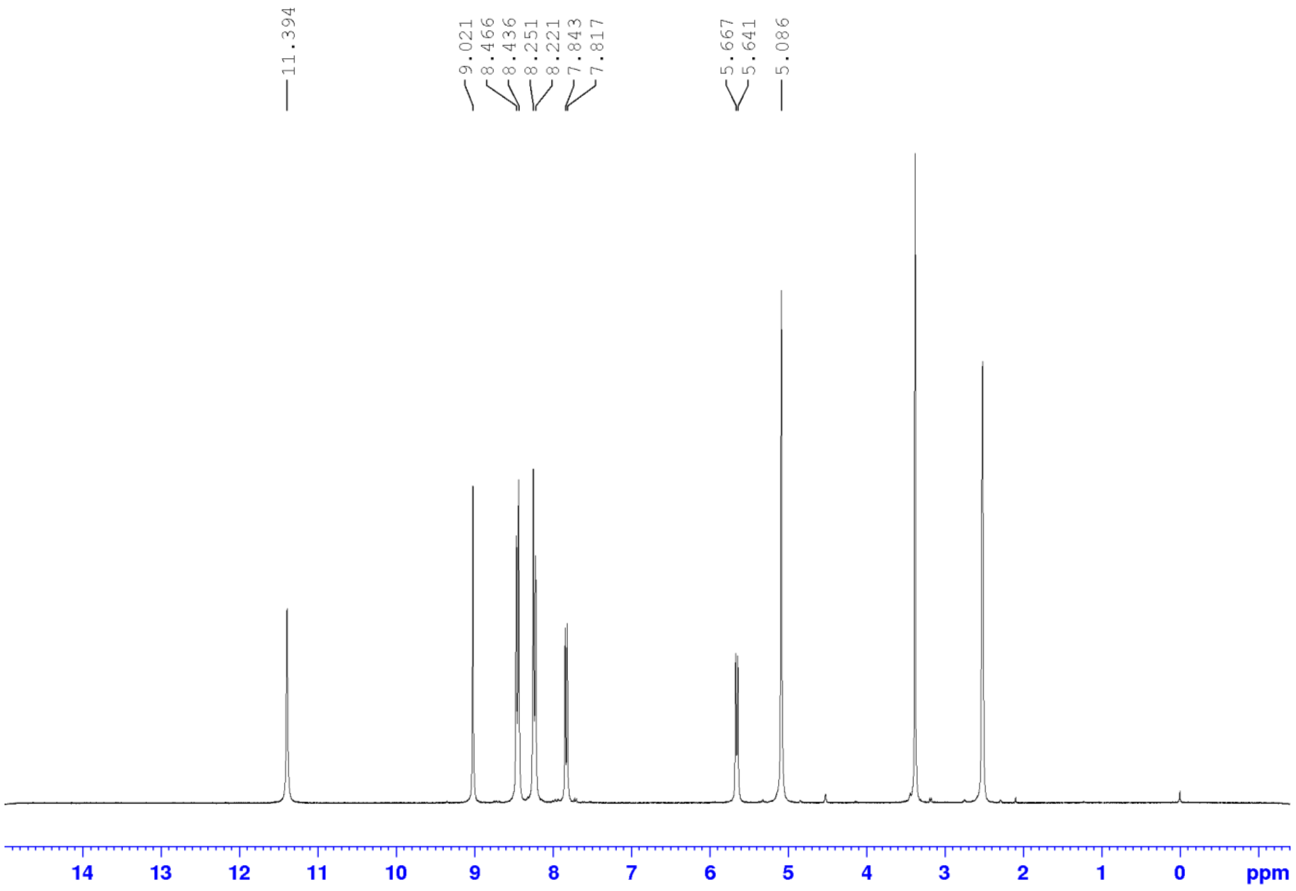

^{13}C NMR

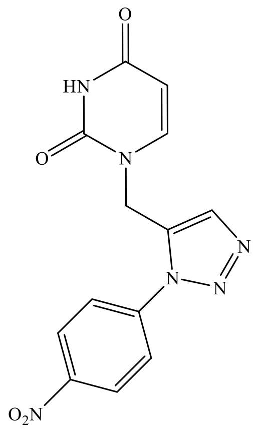

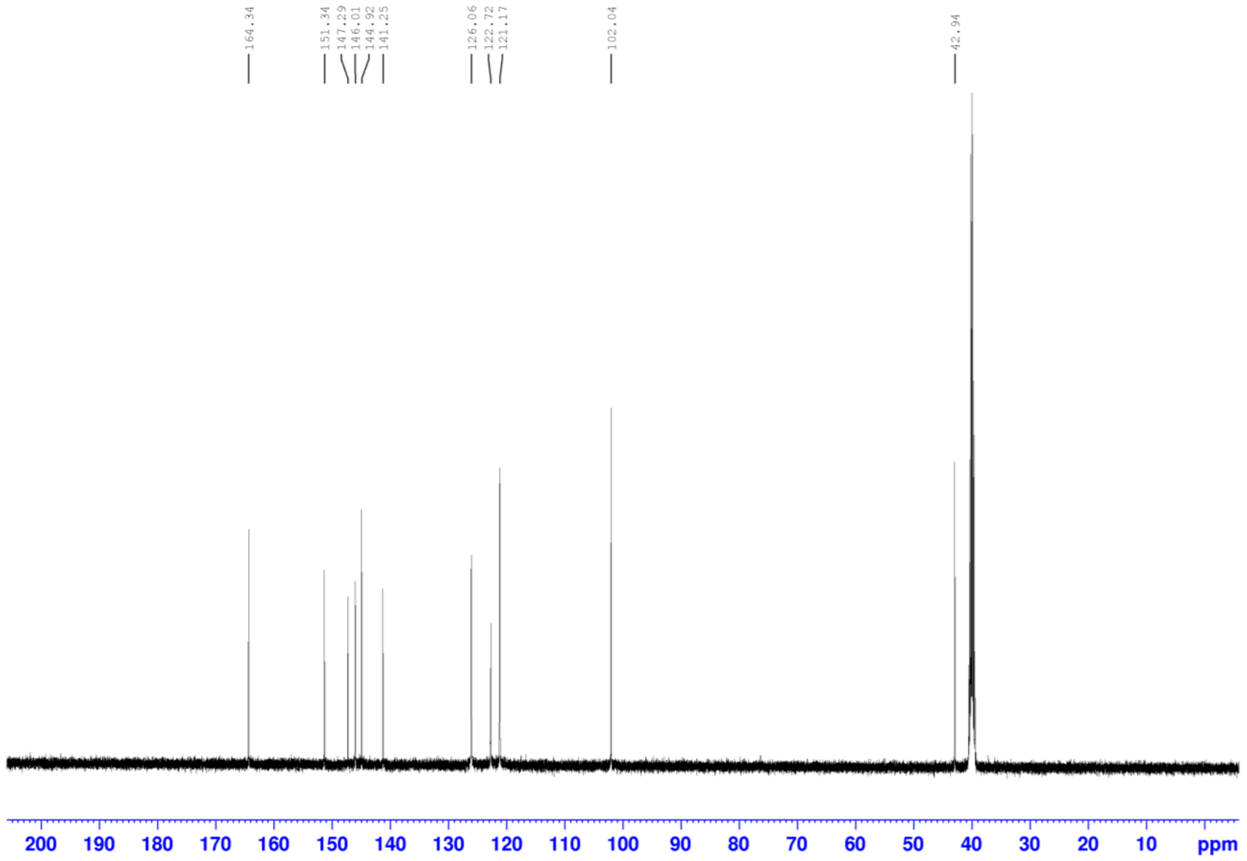

164.34
151.34
147.29
146.01
144.92
141.25
126.06
122.72
121.17
102.04
42.94
200 190 180 170 160 150 140 130 120 110 100 90 80 70 60 50 40 30 20 10 ppm

COSY NMR

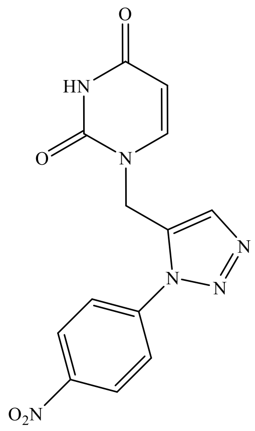

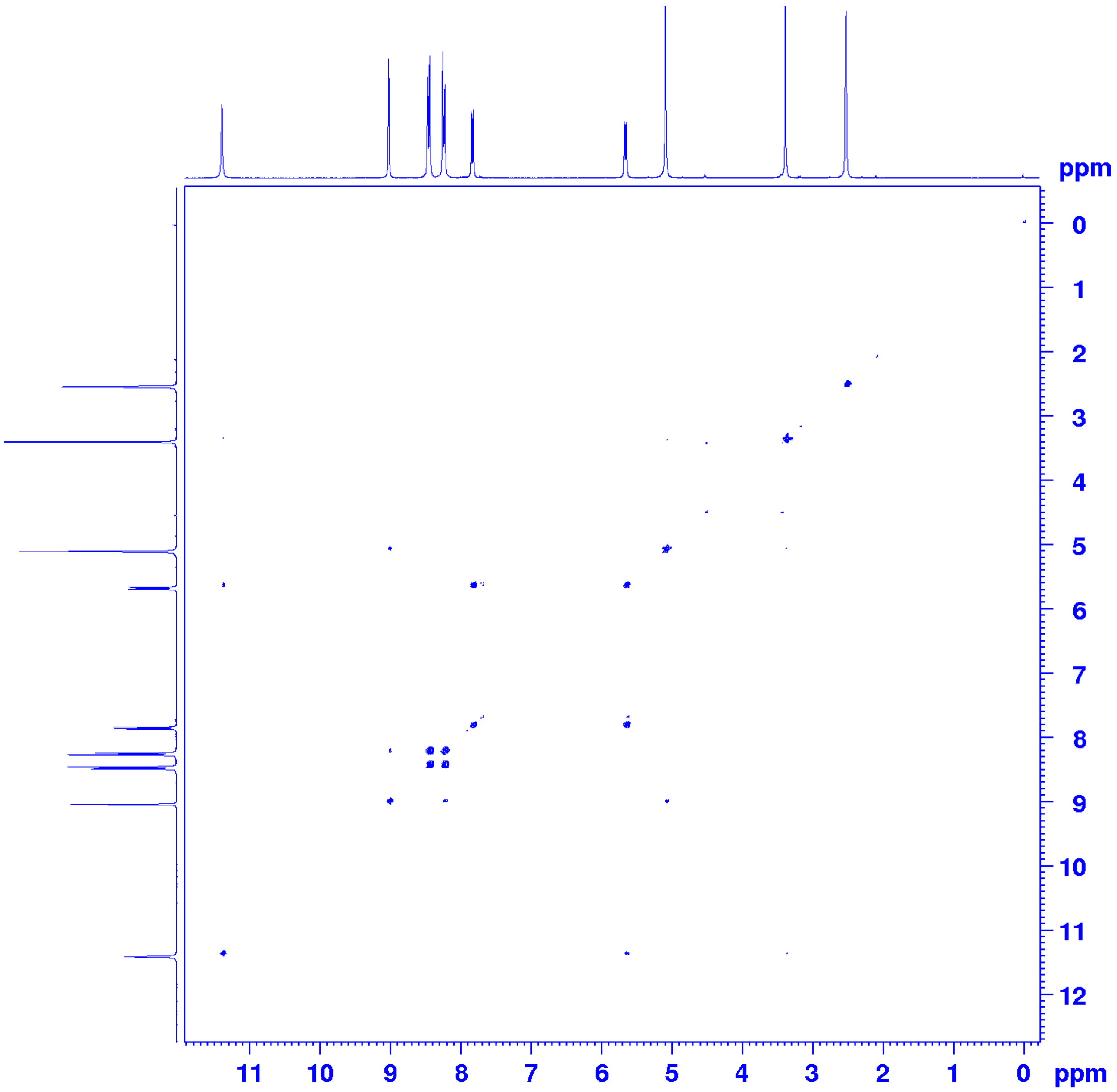

HMBC NMR

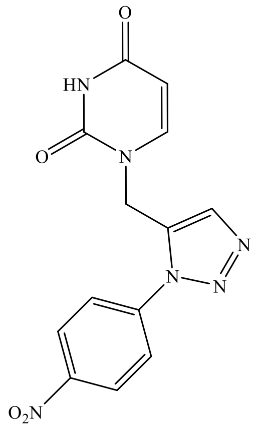
O
HN
O
N
N
N
N
O2N

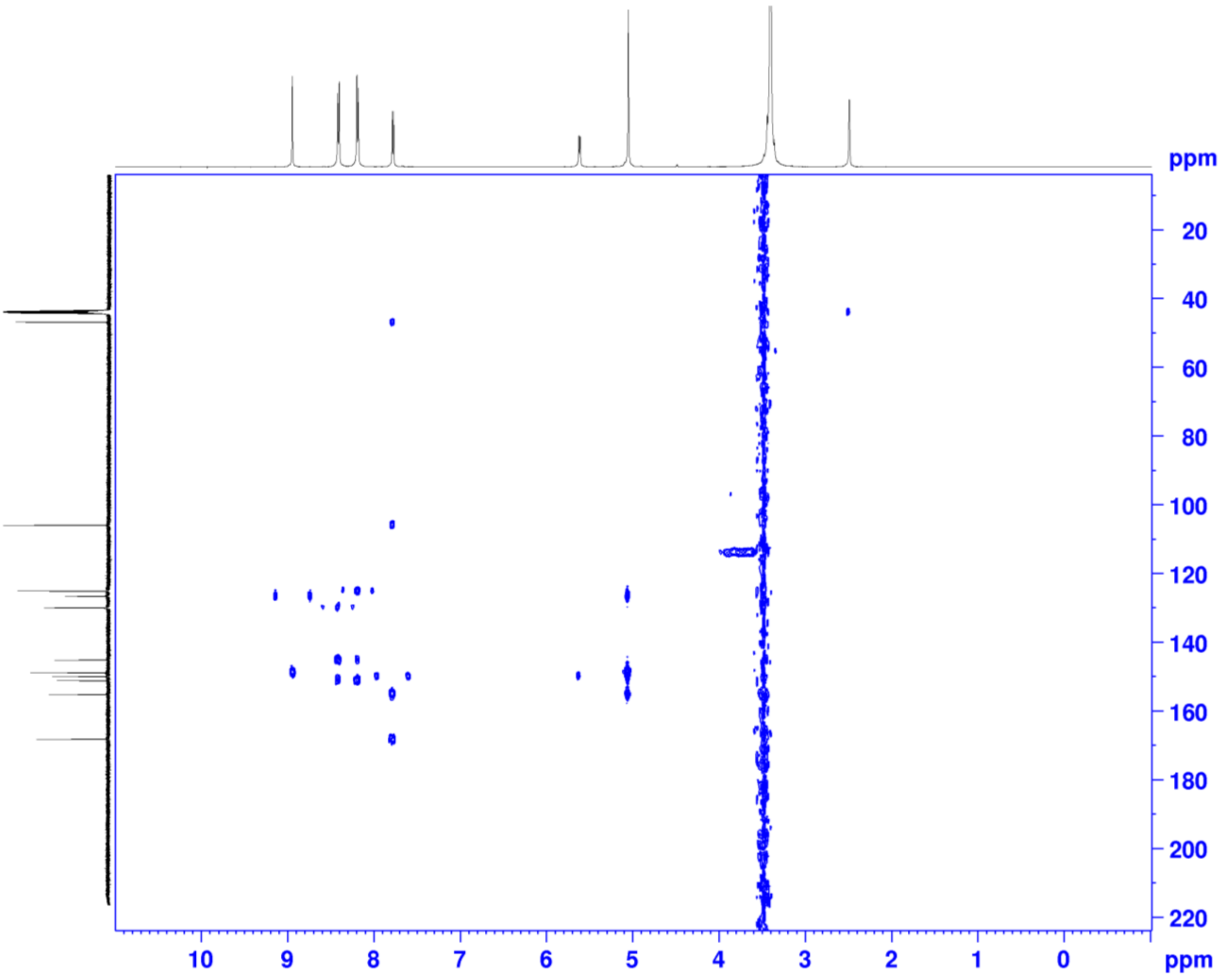
ppm
20
40
60
80
100
120
140
160
180
200
220
10
9
8
7
6
5
4
3
2
1
0
ppm

Magnification of the HMBC NMR spectrum

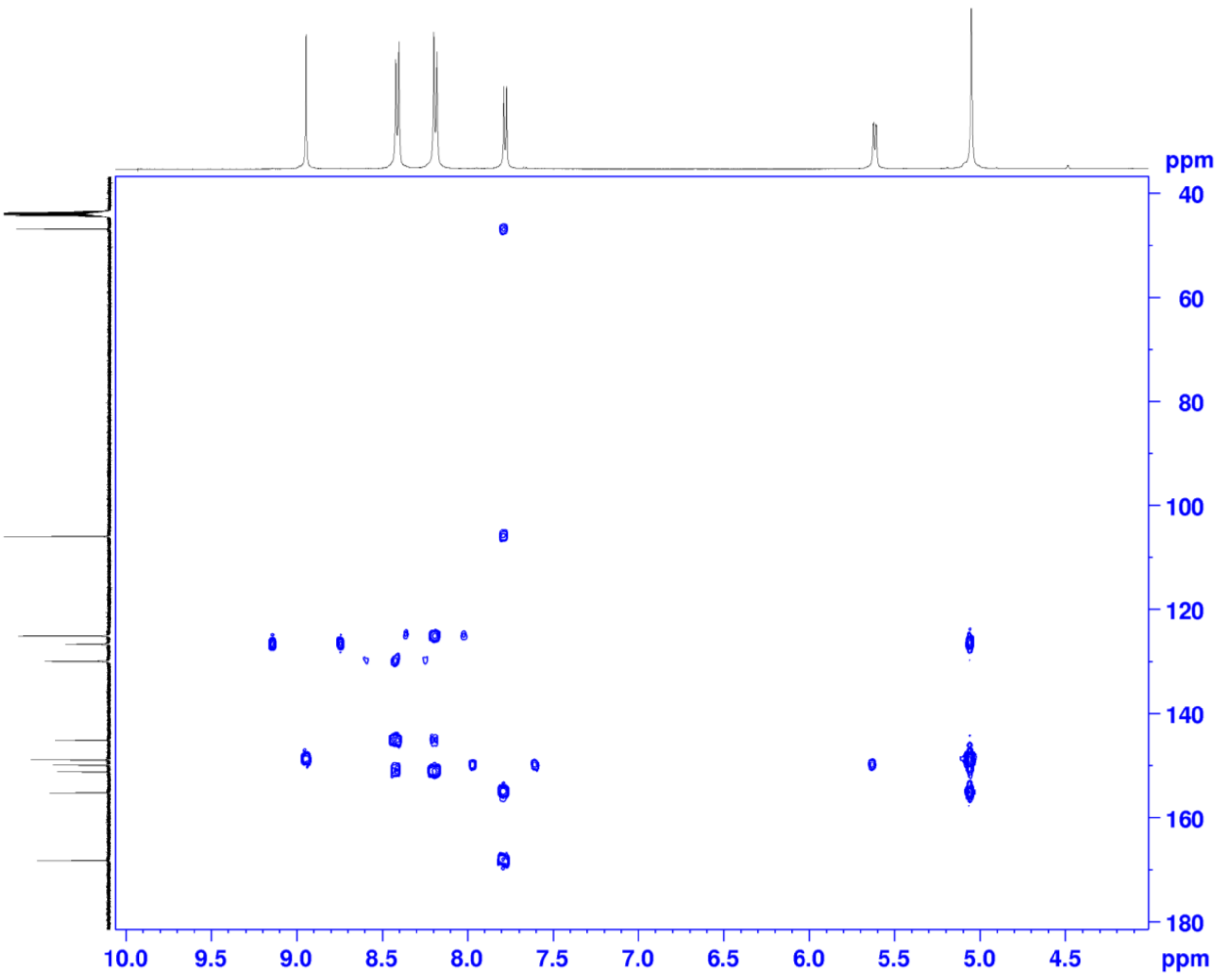

HSQC NMR

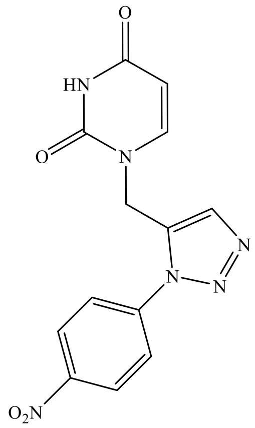
O
HN
O
N
N
N
N
O2N

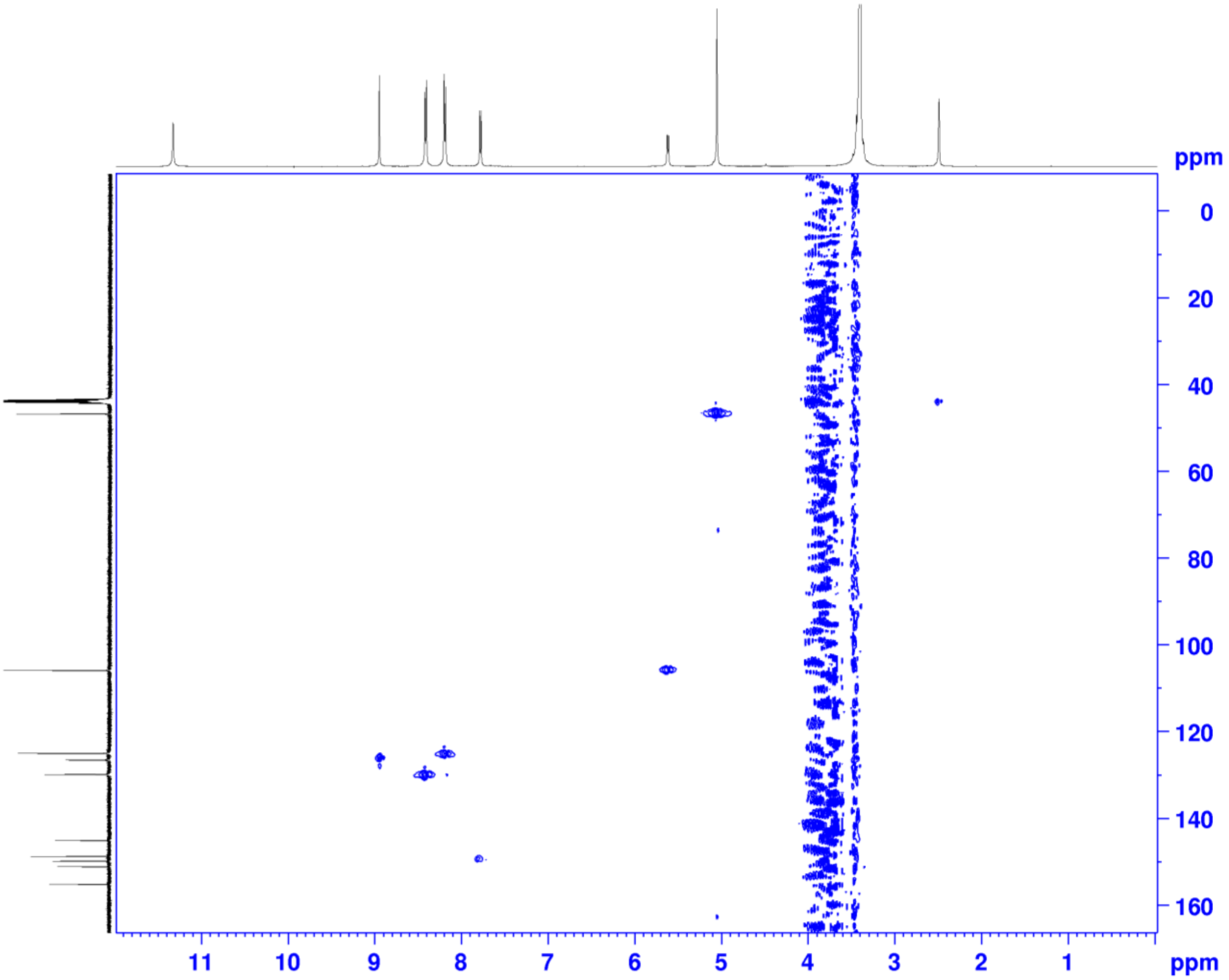
ppm
0
20
40
60
80
100
120
140
160
11
10
9
8
7
6
5
4
3
2
1
ppm

Magnification of the HSQC NMR spectrum

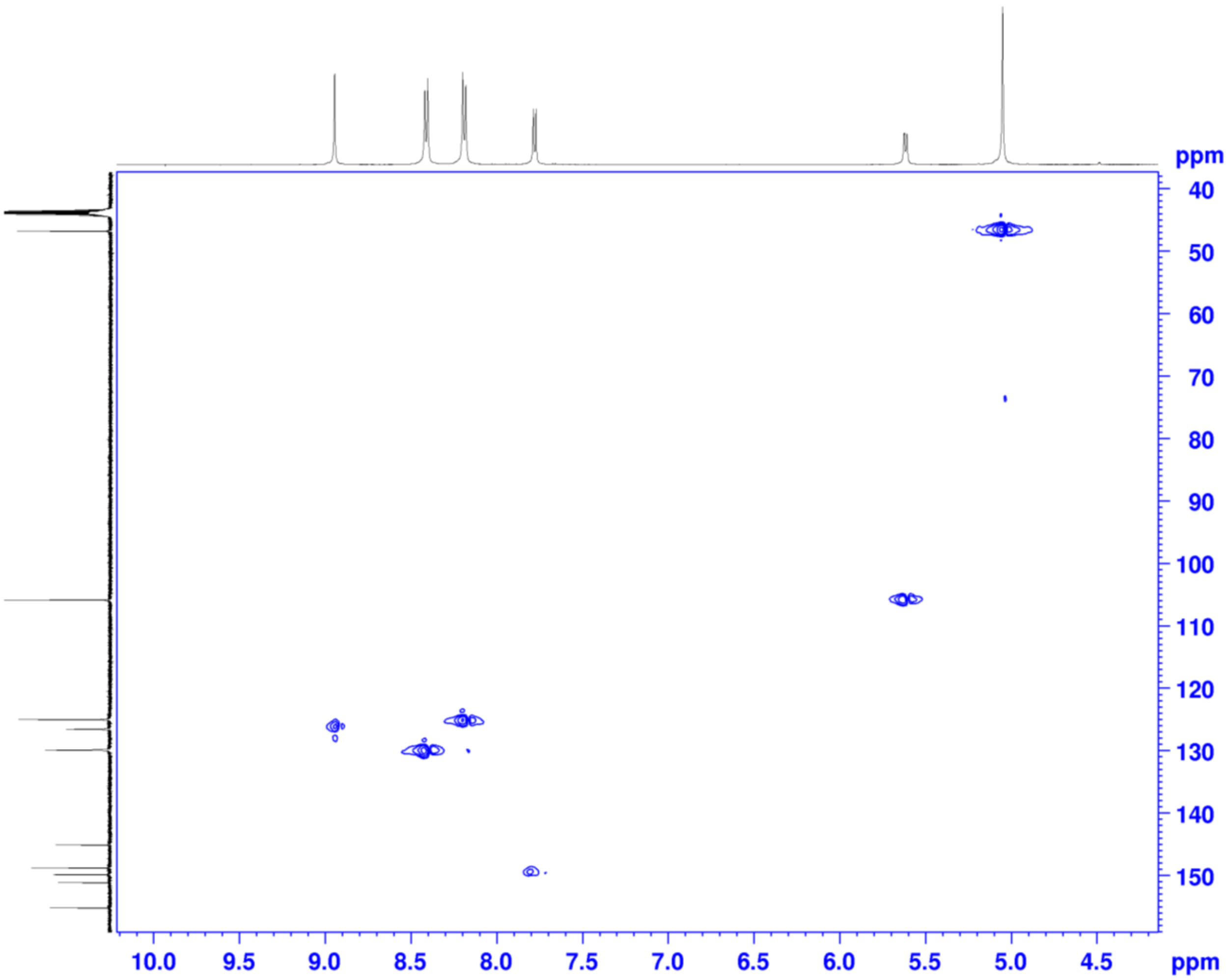

HRMS

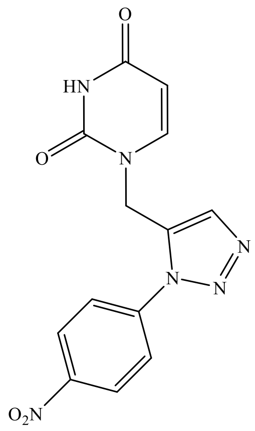
O
HN
O
N
N
N
N
O2N

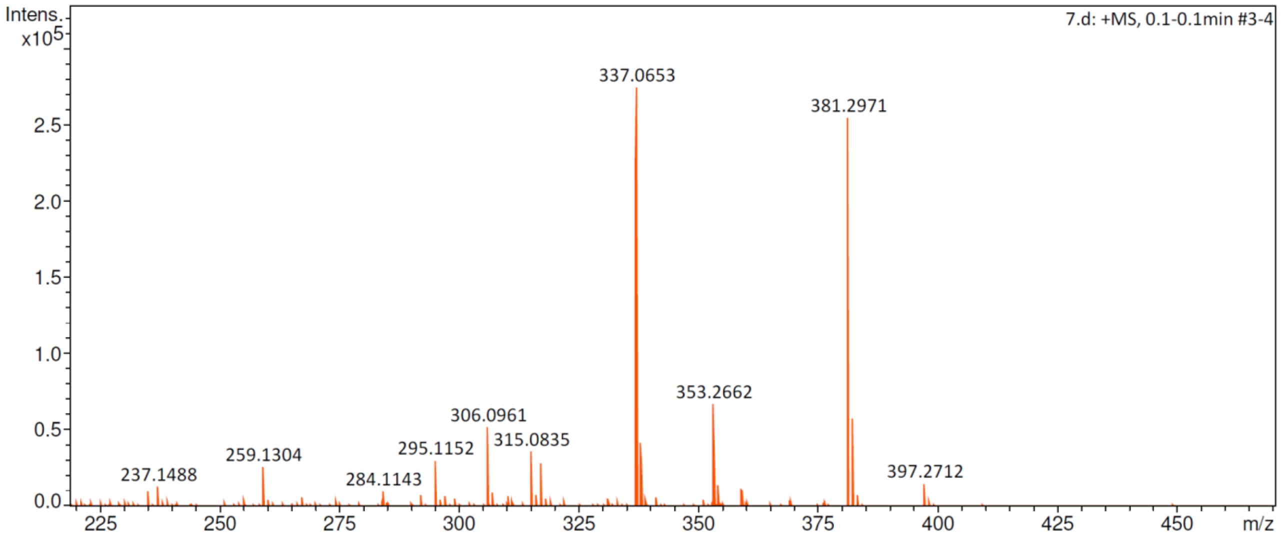
Intens.
x10^5
7.d: +MS, 0.1-0.1min #3-4
337.0653
381.2971
353.2662
306.0961
315.0835
295.1152
259.1304
237.1488
284.1143
397.2712
2.5
2.0
1.5
1.0
0.5
0.0
225
250
275
300
325
350
375
400
425
450
m/z

5-fluoro-1-[1-(4-nitrophenyl)-1,2,3-triazol-5-yl-methyl]-uracil (22a).

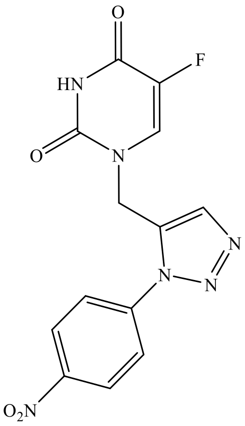

^{1}H NMR

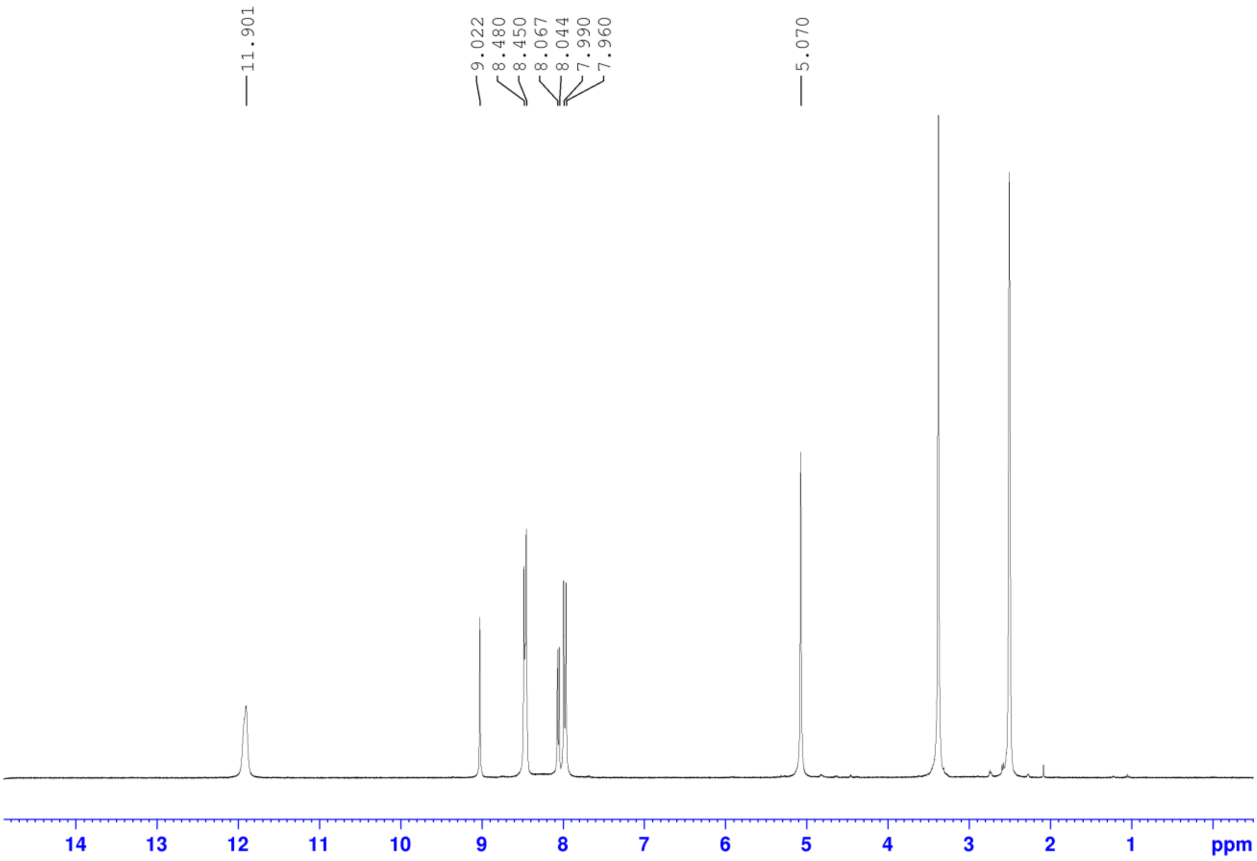

^{13}C NMR

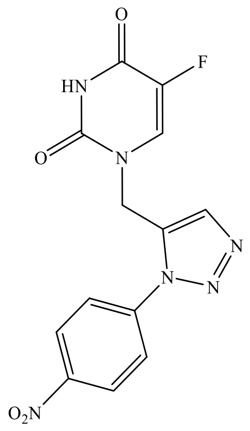

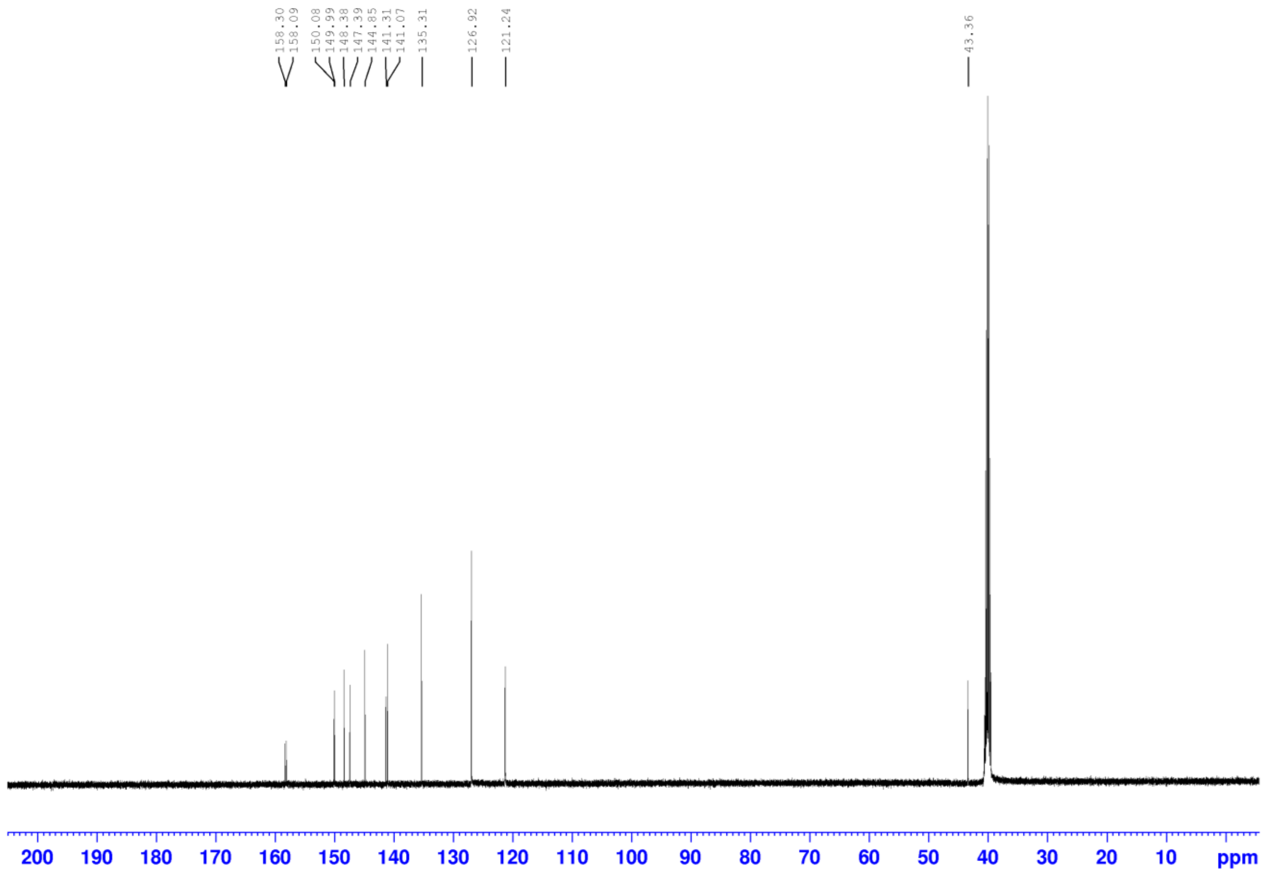
158.30
158.09
150.08
149.99
148.38
147.39
144.85
141.31
141.07
135.31
126.92
121.24
43.36
200 190 180 170 160 150 140 130 120 110 100 90 80 70 60 50 40 30 20 10 ppm

COSY NMR

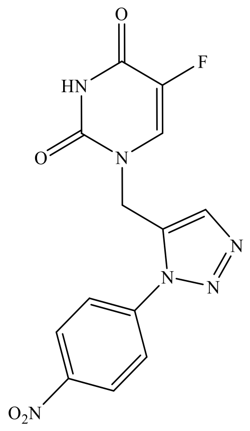

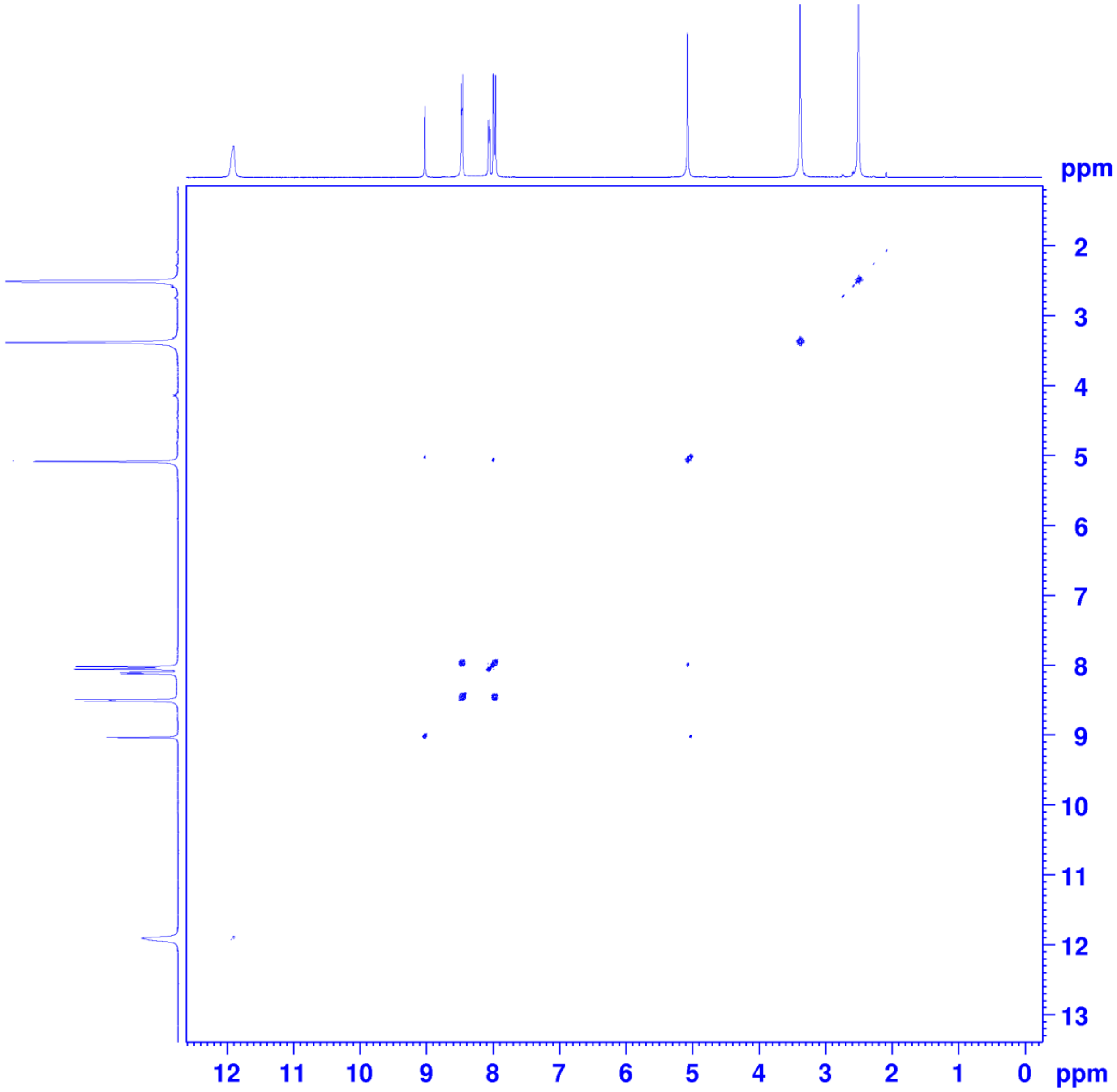

HRMS

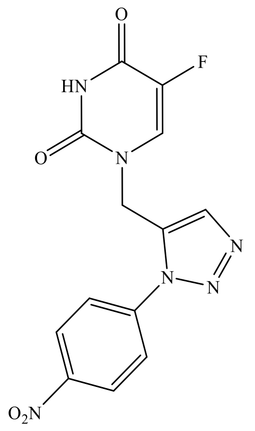

O
F
HN
O
N
N
N
N
O_2N

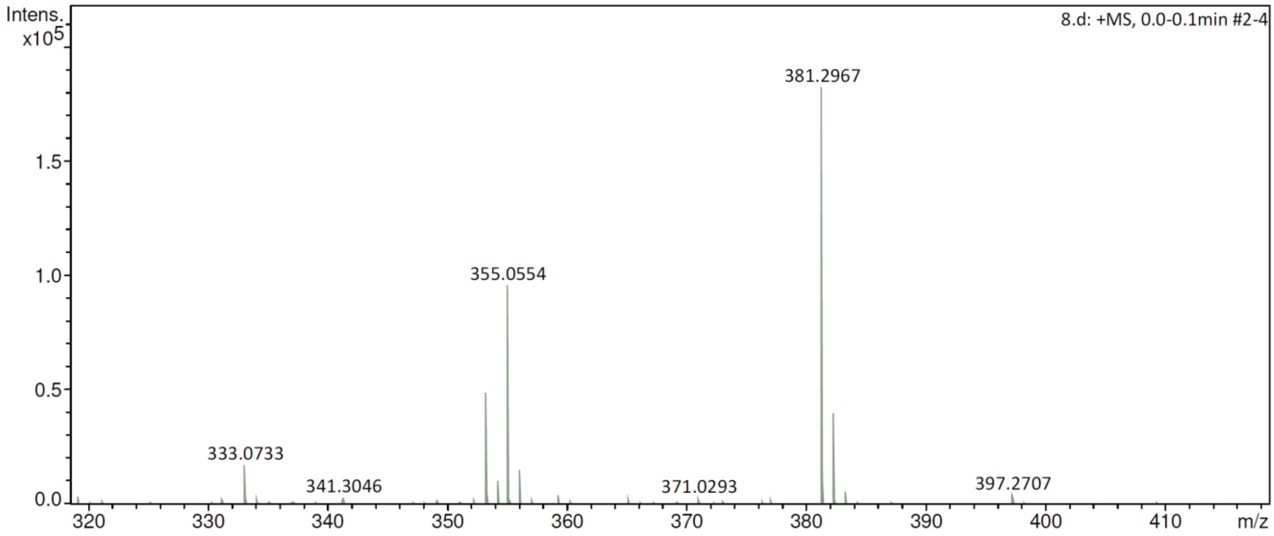

Intens.
x10^5
8.d: +MS, 0.0-0.1min #2-4
381.2967
355.0554
333.0733
341.3046
371.0293
397.2707
1.5
1.0
0.5
0.0
320
330
340
350
360
370
380
390
400
410
m/z

1-[1-(4-methoxyphenyl)-1,2,3-triazol-5-yl-methyl]-thymine (23a).

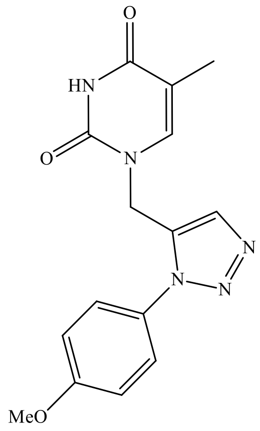

^{1}H NMR

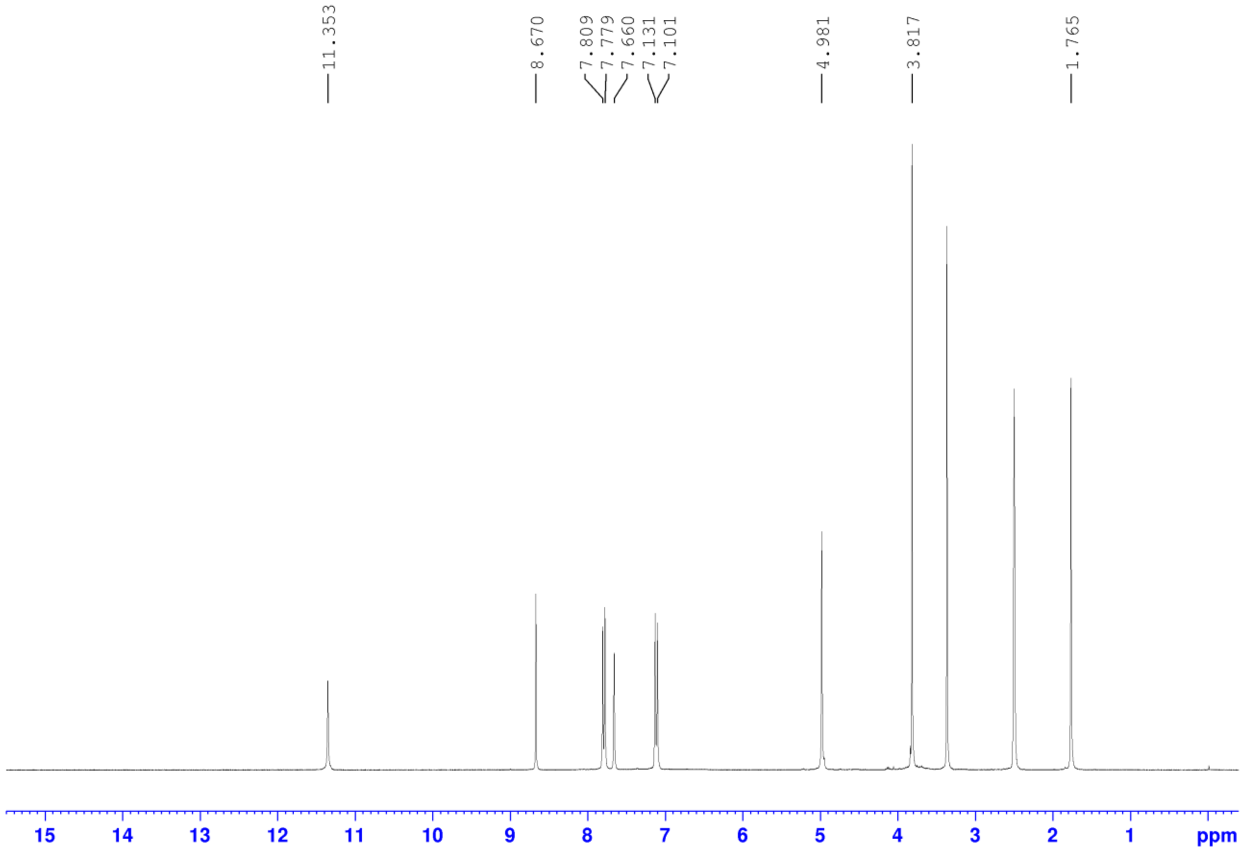

13C NMR

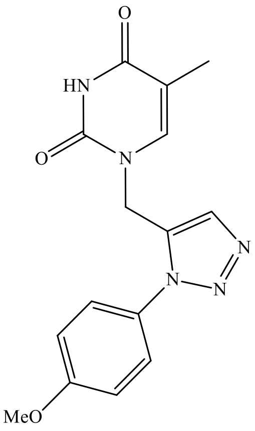
O
HN
O
N
N
N
N
MeO

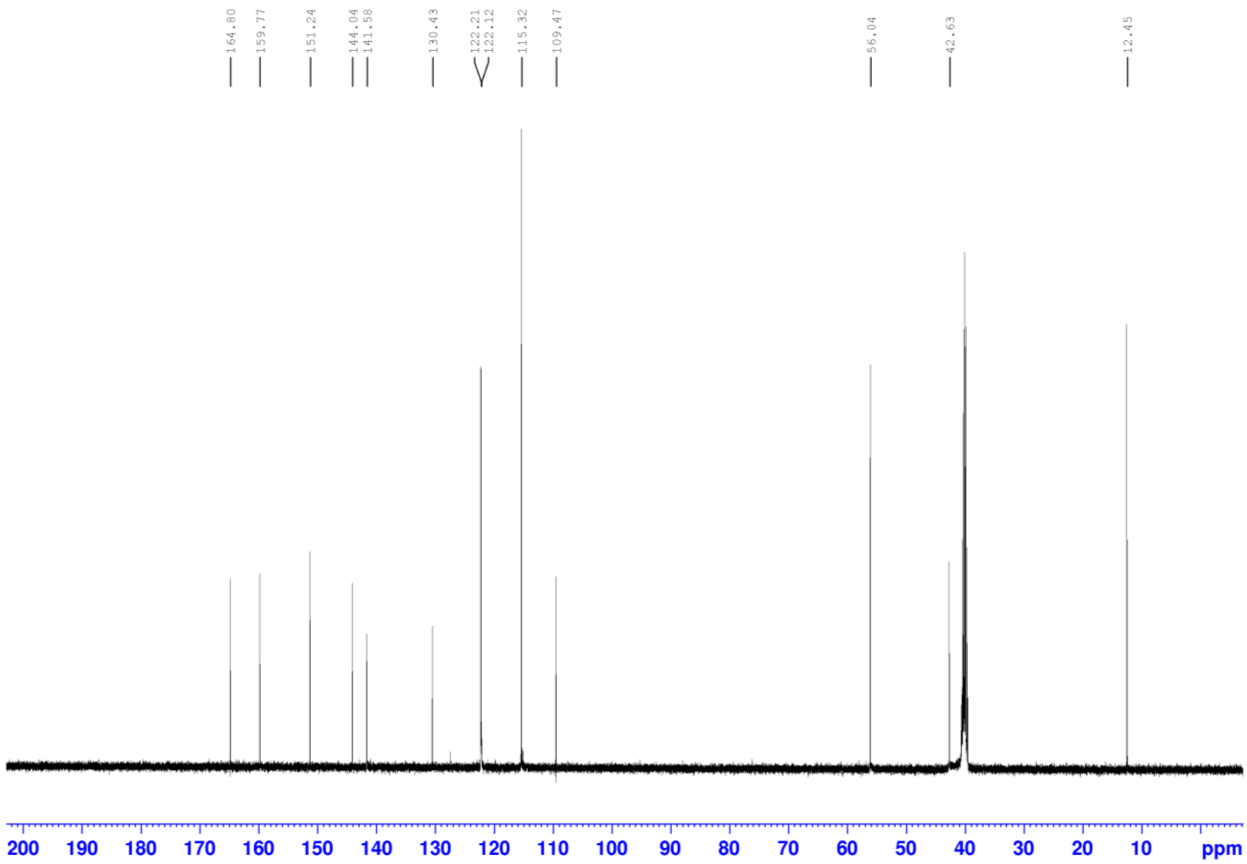
164.80
159.77
151.24
144.04
141.58
130.43
122.21
122.12
115.32
109.47
56.04
42.63
12.45
200
190
180
170
160
150
140
130
120
110
100
90
80
70
60
50
40
30
20
10
ppm

COSY NMR

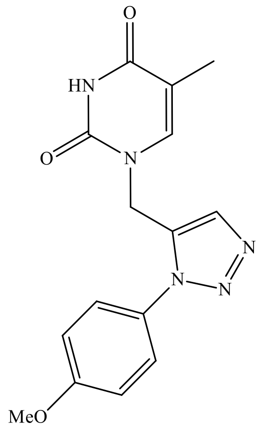

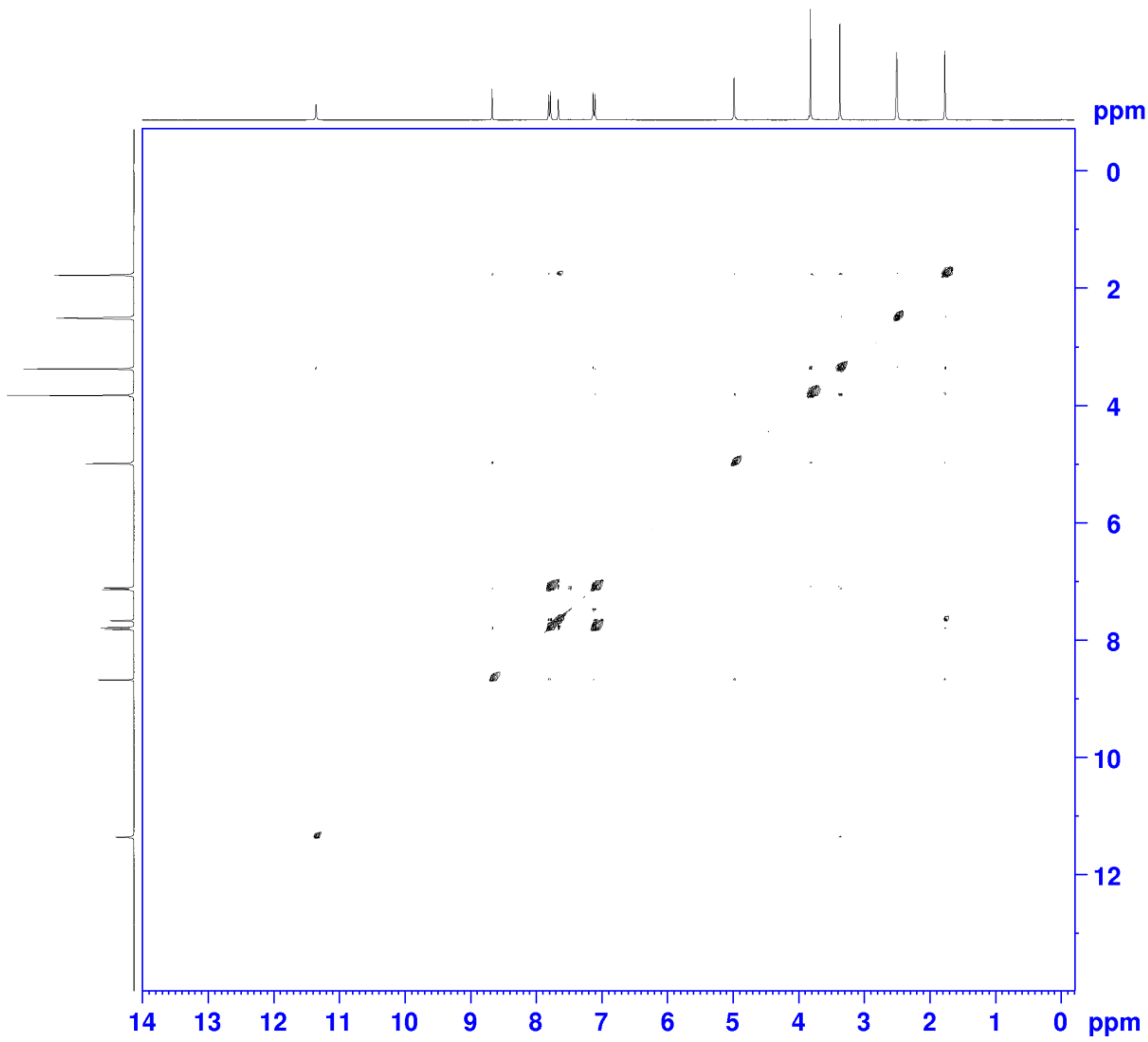

HRMS

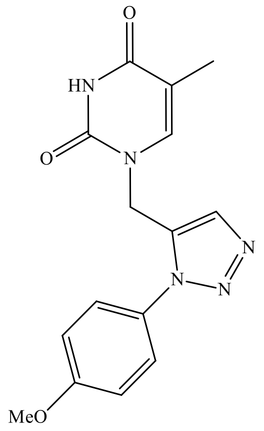
O
HN
O
N
N
N
N
MeO

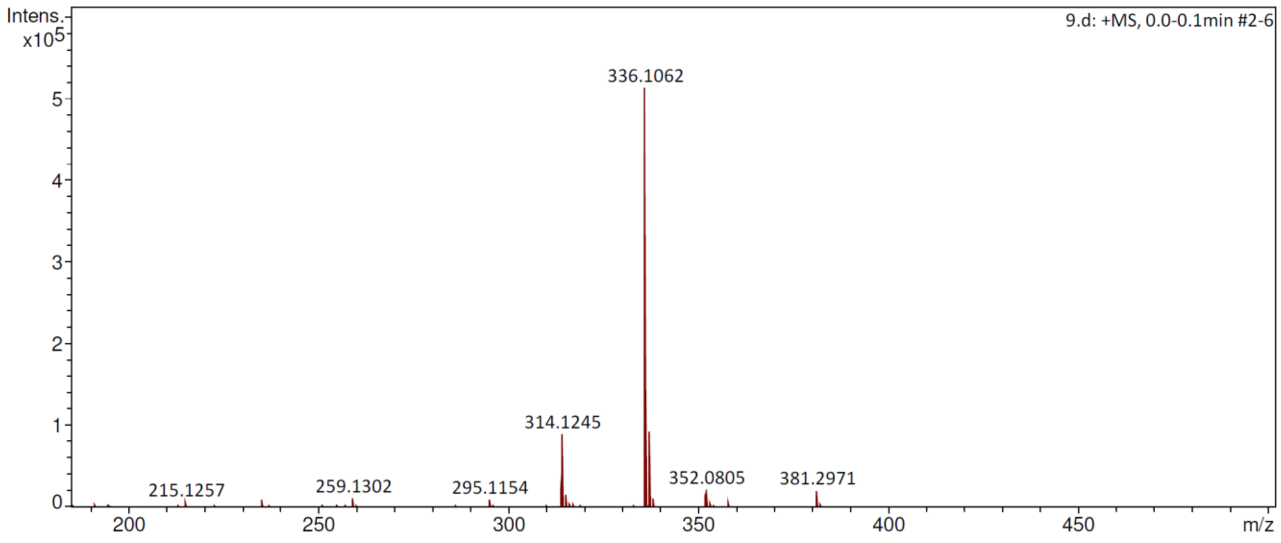
Intens.
x10^5
9.d: +MS, 0.0-0.1min #2-6
336.1062
314.1245
215.1257
259.1302
295.1154
352.0805
381.2971
0
1
2
3
4
5
200
250
300
350
400
450
m/z

1-[1-(4-methoxyphenyl)-1,2,3-triazol-5-yl-methyl]-uracil (24a).

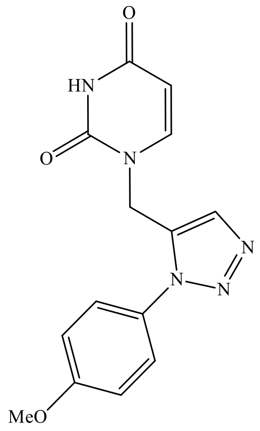

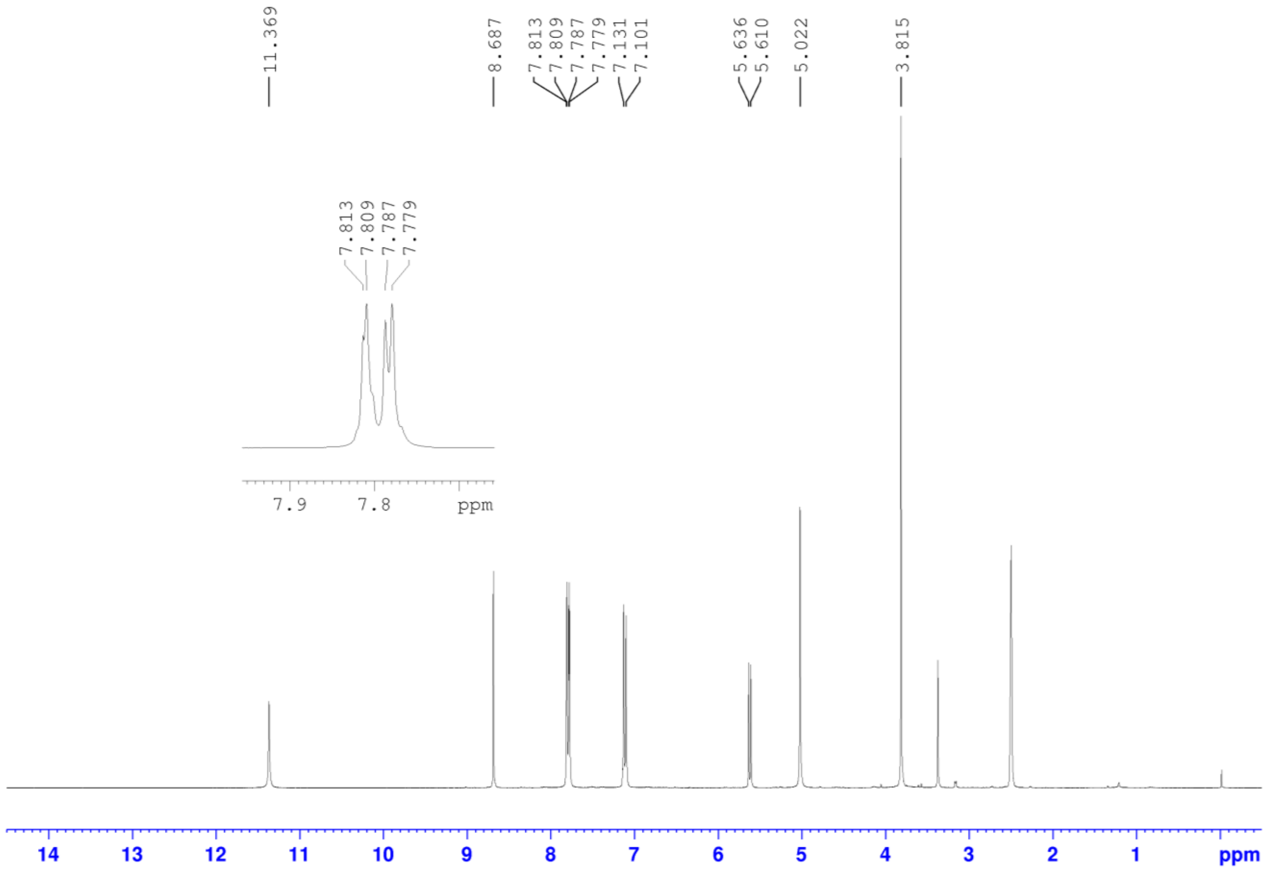

^{13}C NMR

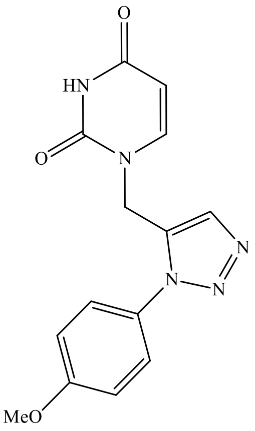

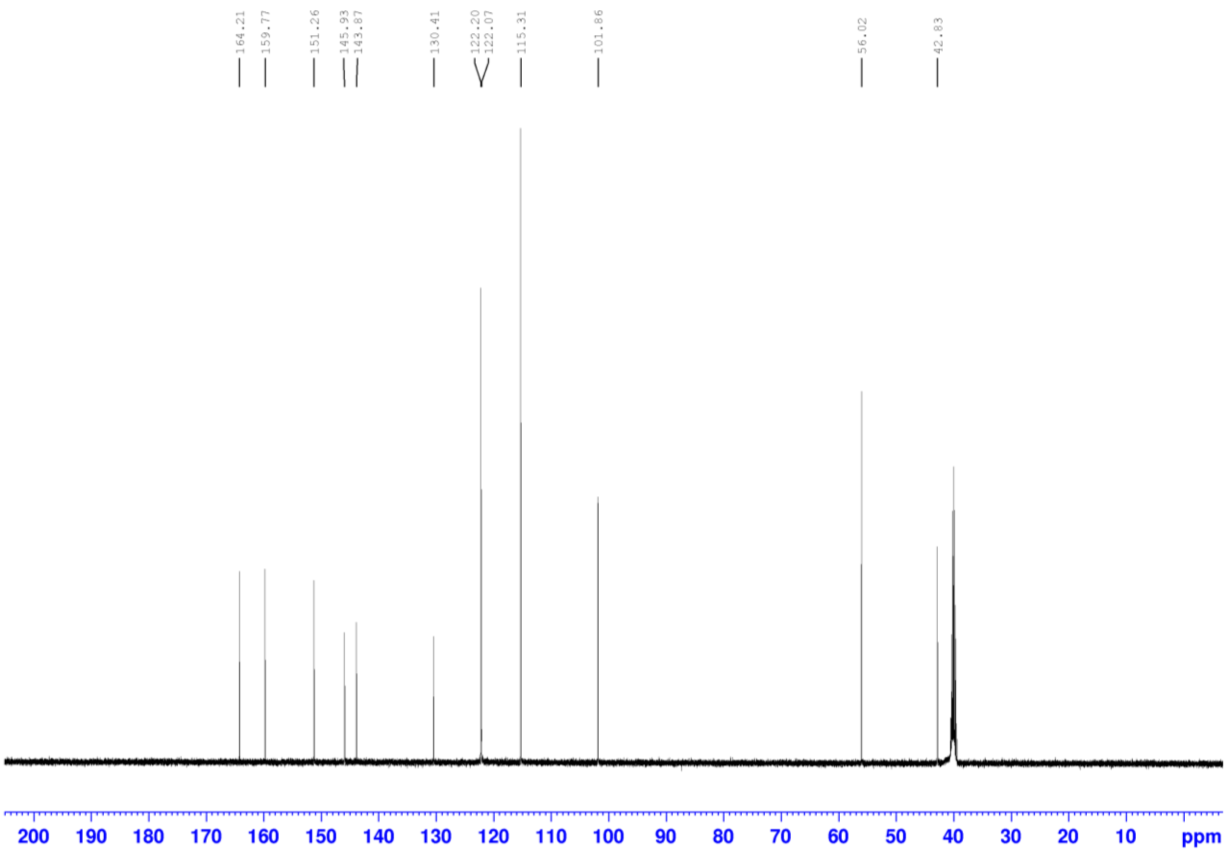
164.21
159.77
151.26
145.93
143.87
130.41
122.20
122.07
115.31
101.86
56.02
42.83
200 190 180 170 160 150 140 130 120 110 100 90 80 70 60 50 40 30 20 10 ppm

COSY NMR

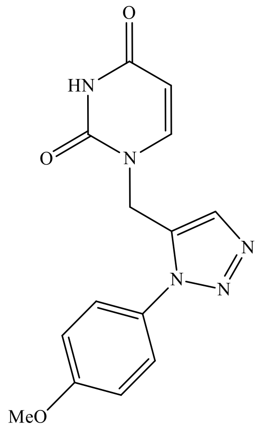
O
HN
O
N
N
N
N
MeO

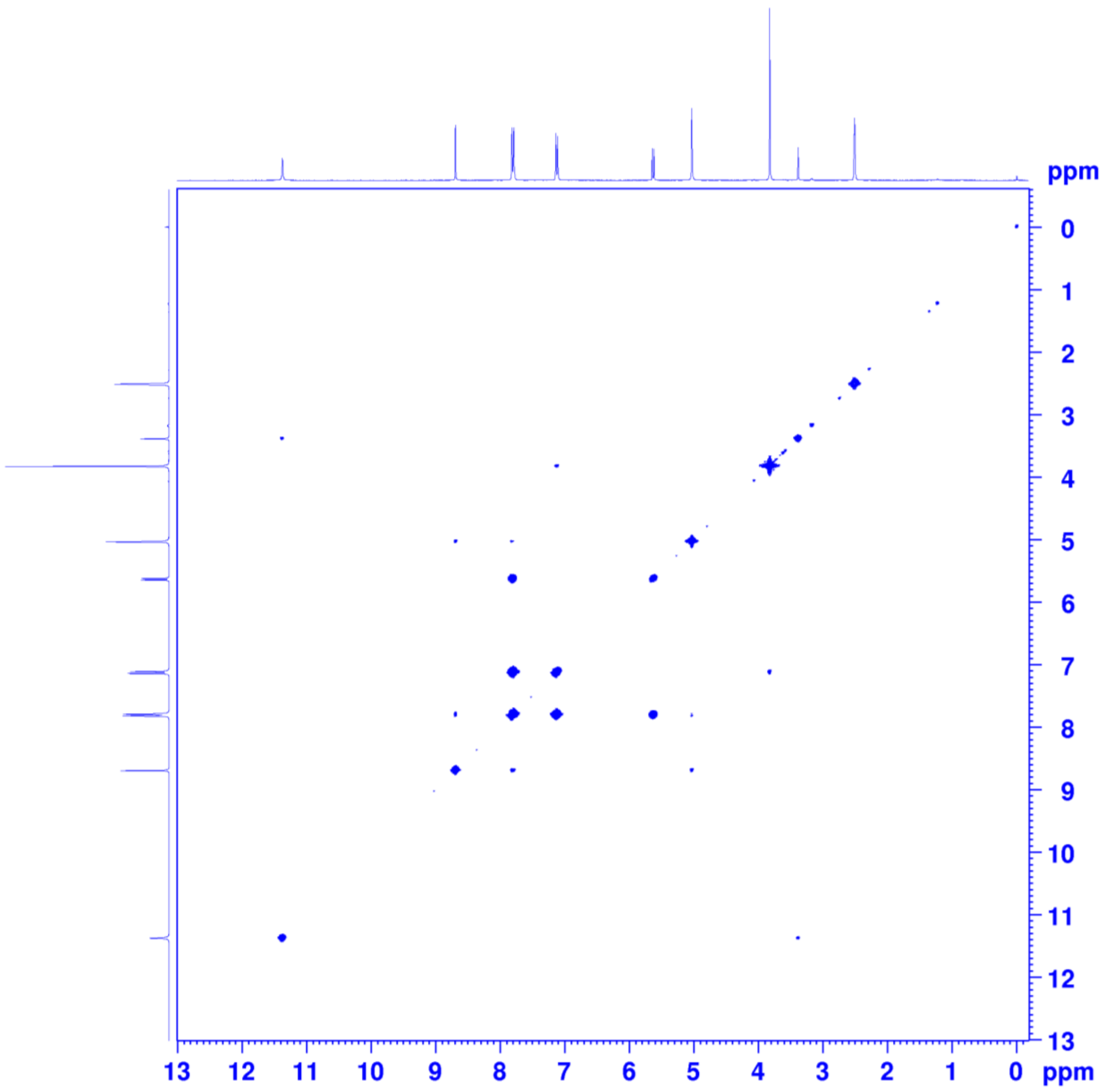
ppm
0
1
2
3
4
5
6
7
8
9
10
11
12
13
13 12 11 10 9 8 7 6 5 4 3 2 1 0 ppm

HRMS

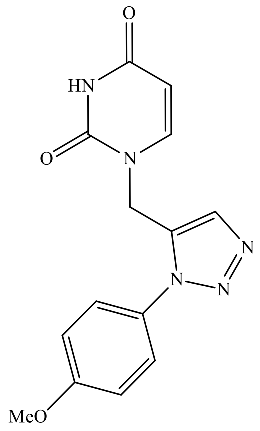
O
HN
O
N
N
N
N
MeO

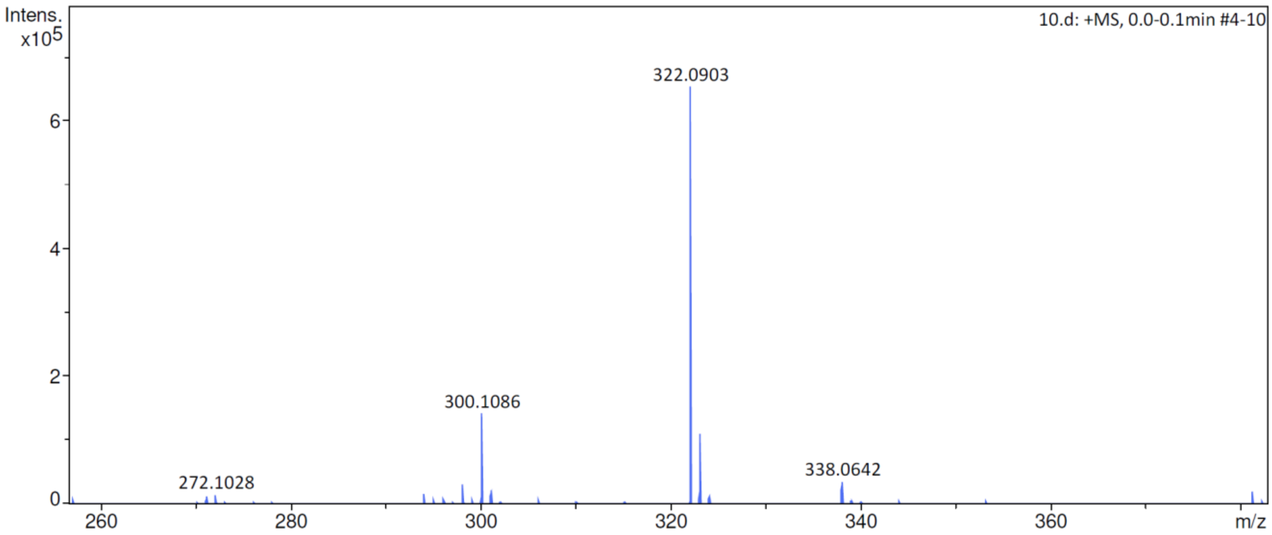
Intens.
x10^5
10.d: +MS, 0.0-0.1min #4-10
322.0903
300.1086
272.1028
338.0642
6
4
2
0
260
280
300
320
340
360
m/z

5-fluoro-1-[1-(4-methoxyphenyl)-1,2,3-triazol-5-yl-methyl]-uracil (25a)

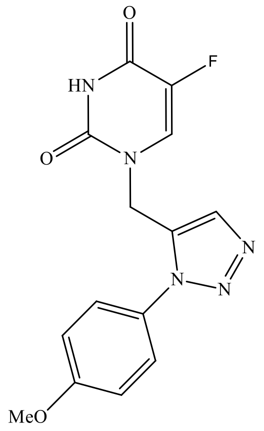

^{1}H NMR

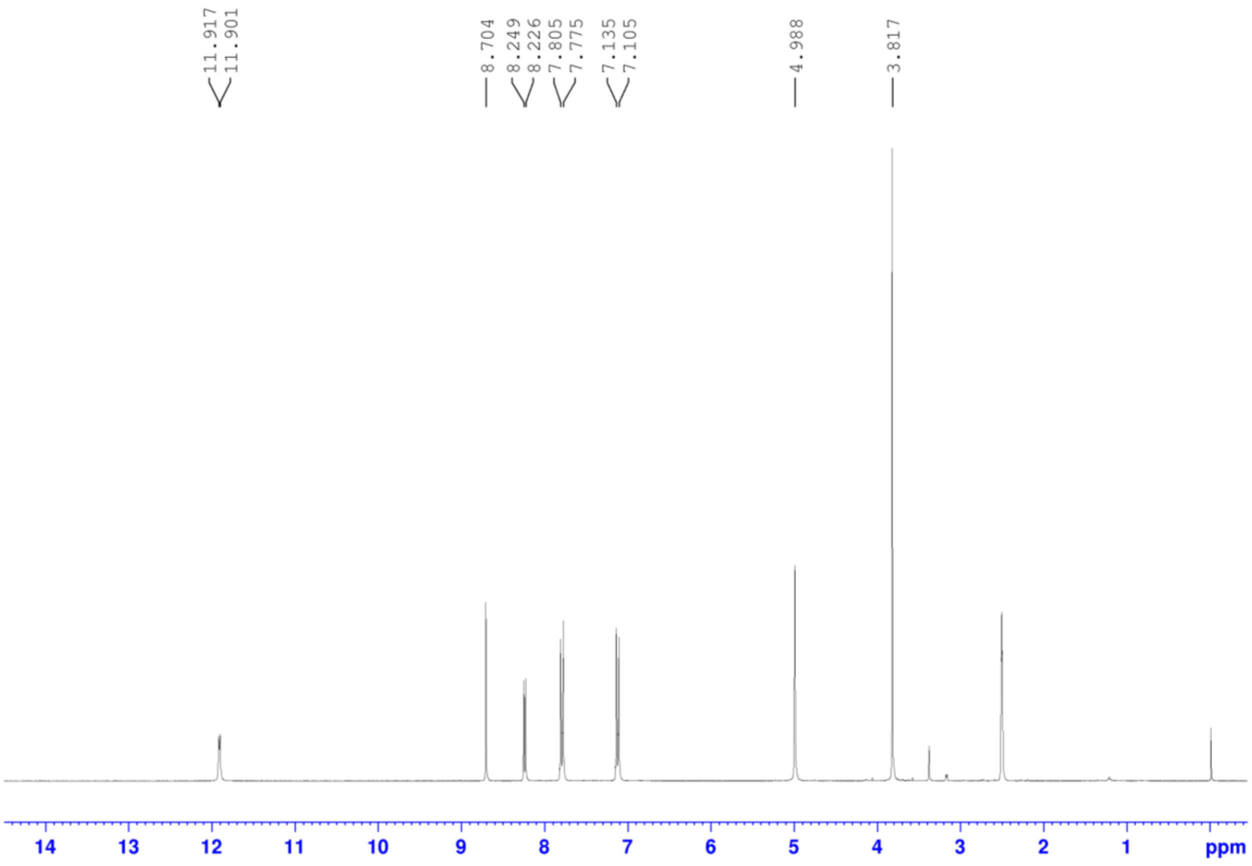

^{13}C NMR

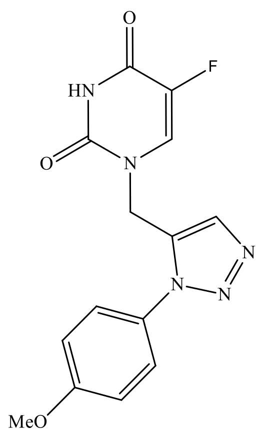
O
F
HN
O
N
N
N
N
MeO

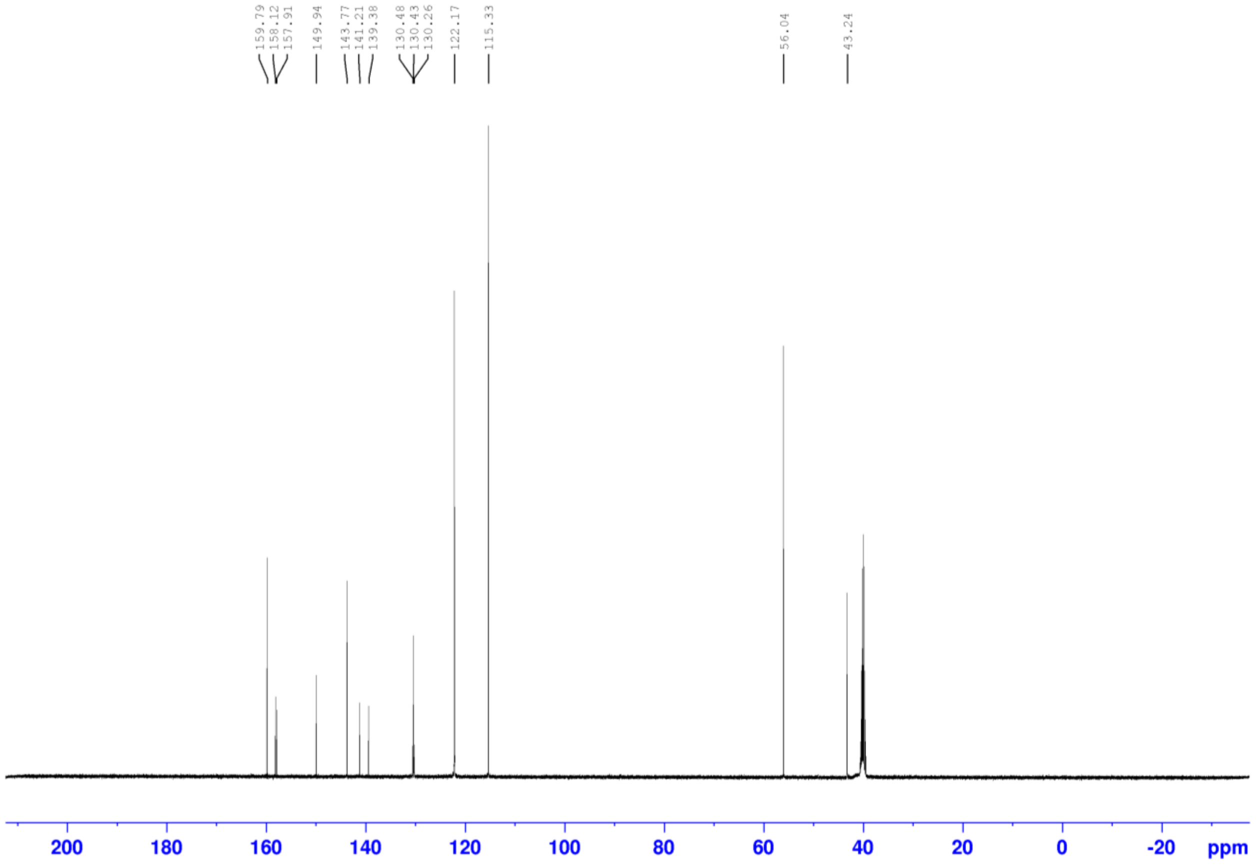
159.79
158.12
157.91
149.94
143.77
141.21
139.38
130.48
130.43
130.26
122.17
115.33
56.04
43.24
200
180
160
140
120
100
80
60
40
20
0
-20
ppm

COSY NMR

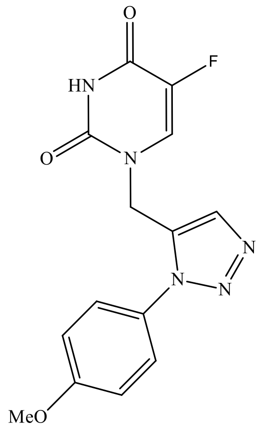
O
F
HN
O
N
N
N
N
MeO

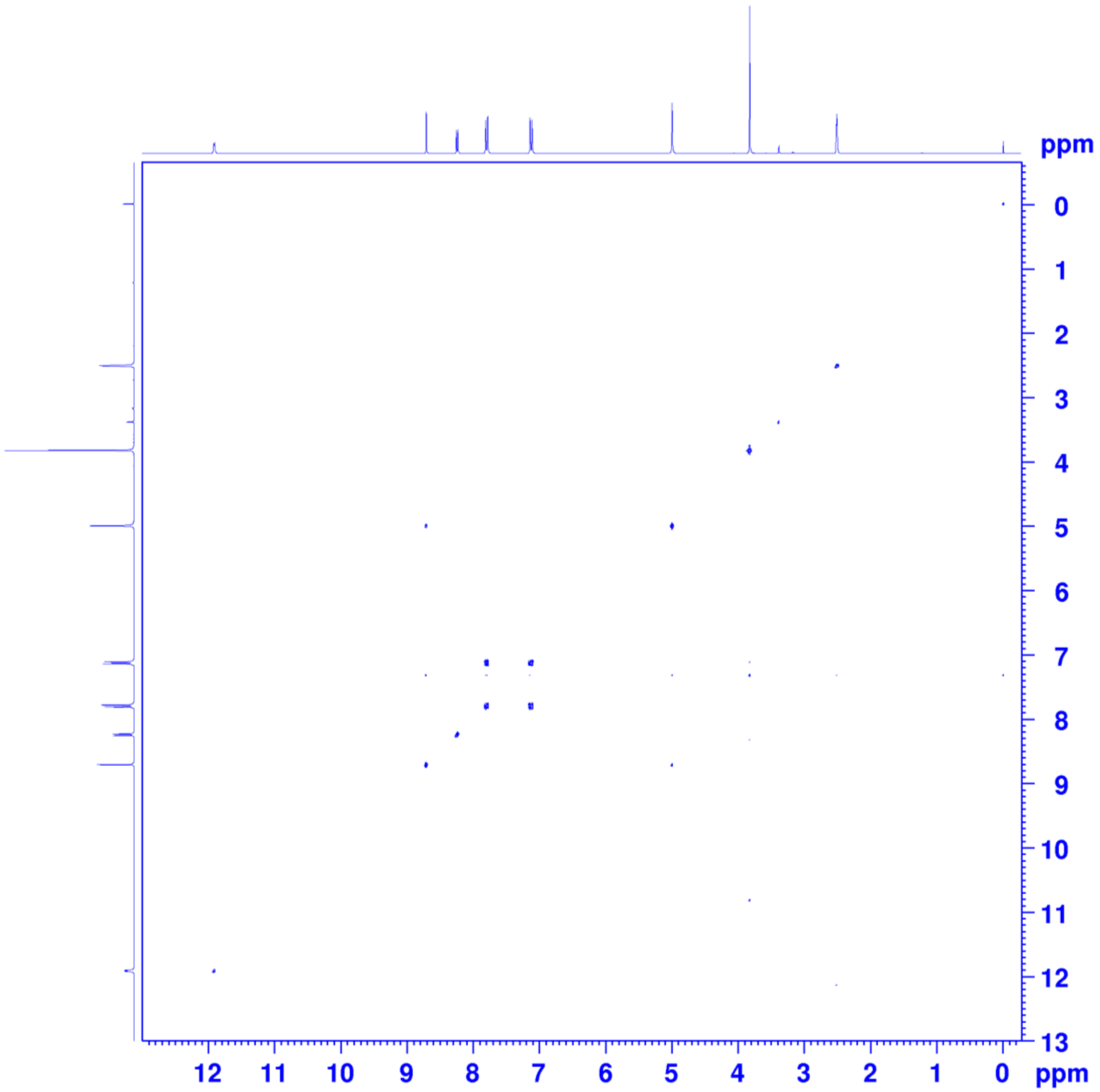
ppm
0
1
2
3
4
5
6
7
8
9
10
11
12
13
12 11 10 9 8 7 6 5 4 3 2 1 0 ppm

HRMS

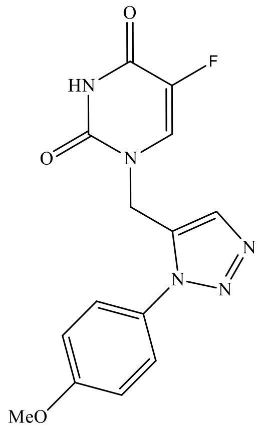

O
F
HN
O
N
N
N
N
MeO

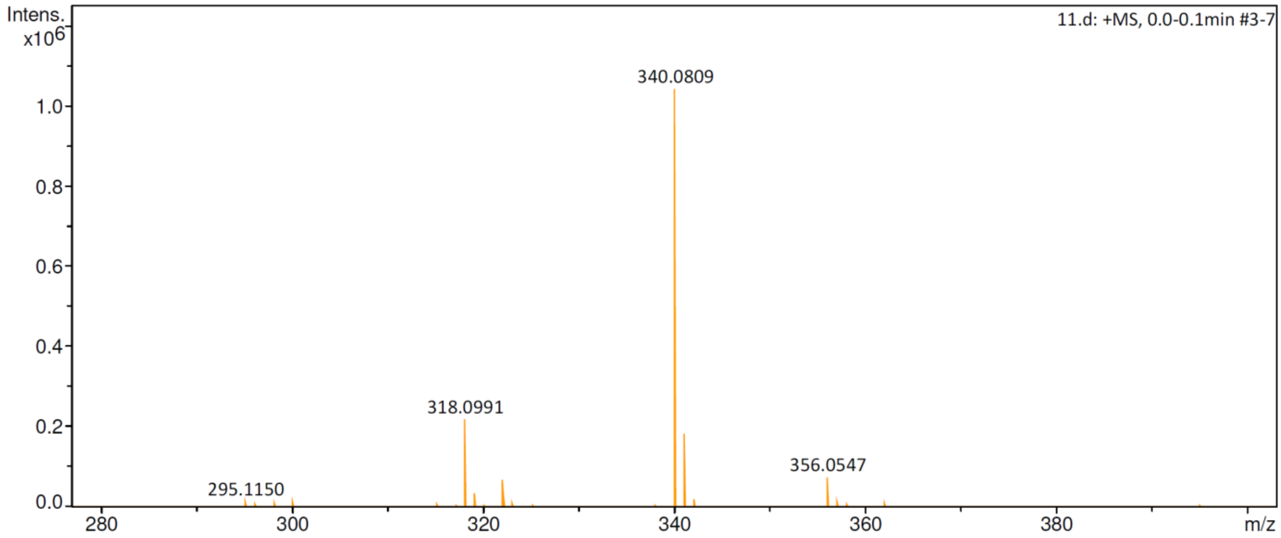

Intens.
x10^6
11.d: +MS, 0.0-0.1min #3-7
340.0809
318.0991
356.0547
295.1150
1.0
0.8
0.6
0.4
0.2
0.0
280
300
320
340
360
380
m/z
